# Phylodynamic Analysis of Ebola Virus Disease Transmission in Sierra Leone

**DOI:** 10.3390/v11010071

**Published:** 2019-01-16

**Authors:** Petrus Jansen van Vuren, Jason T. Ladner, Antoinette A. Grobbelaar, Michael R. Wiley, Sean Lovett, Mushal Allam, Arshad Ismail, Chantel le Roux, Jacqueline Weyer, Naazneen Moolla, Nadia Storm, Joe Kgaladi, Mariano Sanchez-Lockhart, Ousman Conteh, Gustavo Palacios, Janusz T. Paweska

**Affiliations:** 1 Centre for Emerging Zoonotic and Parasitic Diseases, National Institute for Communicable Diseases of the National Health Laboratory Service, Sandringham 2131, South Africa; petrusv@nicd.ac.za (P.J.v.V); antoinetteg@nicd.ac.za (A.A.G.); chantell@nicd.ac.za (C.l.R.); jacquelinew@nicd.ac.za (J.W.); naazneenm@nicd.ac.za (N.M.); nadias@nicd.ac.za (N.S.); joek@nicd.ac.za (J.K.); 2 Center for Genome Sciences, United States Army Medical Research Institute for Infectious Diseases, Fort Detrick, MD 21702, USA; jtladner@gmail.com (J.T.L.) michael.r.wiley19.ctr@mail.mil (M.R.W.); Lovett05@gmail.com (S.L.); mariano.sanchez-lockhart.ctr@mail.mil (M.S.-L.); gustavo.f.palacios.ctr@mail.mil (G.P.); 3 The Pathogen and Microbiome Institute, Northern Arizona University, Flagstaff, AZ 86001, USA; 4 Department of Environmental, Agricultural & Occupational Health, College of Public Health, University of Nebraska Medical Center, Omaha, NE 68198, USA; 5 Sequencing Core Facility, National Institute for Communicable Diseases of the National Health Laboratory Service, Sandringham 2131, South Africa; mushala@nicd.ac.za (M.A.); arshadi@nicd.ac.za (A.I.); 6 Ministry of Health and Sanitation, Freetown 47235, Sierra Leone; osconteh@gmail.com; 7 Faculty of Health Sciences, University of the Witwatersrand, Johannesburg 2050, South Africa

**Keywords:** Ebola virus, filovirus, *Filoviridae*, phylodynamics, Ebola virus disease, Sierra Leone, West Africa

## Abstract

We generated genome sequences from 218 cases of Ebola virus disease (EVD) in Sierra Leone (SLE) during 2014–2015 to complement available datasets, particularly by including cases from a period of low sequence coverage during peak transmission of Ebola virus (EBOV) in the highly-affected Western Area division of SLE. The combined dataset was utilized to produce phylogenetic and phylodynamic inferences, to study sink–source dynamics and virus dispersal from highly-populated transmission hotspots. We identified four districts in SLE where EBOV was introduced and transmission occurred without onward exportation to other districts. We also identified six districts that substantially contributed to the dispersal of the virus and prolonged the EVD outbreak: five of these served as major hubs, with lots of movement in and out, and one acted primarily as a source, exporting the virus to other areas of the country. Positive correlations between case numbers, inter-district transition events, and district population sizes reaffirm that population size was a driver of EBOV transmission dynamics in SLE. The data presented here confirm the role of urban hubs in virus dispersal and of a delayed laboratory response in the expansion and perpetuation of the EVD outbreak in SLE.

## 1. Introduction

Between 2013 and 2016, the West African countries of Guinea, Liberia, Sierra Leone, Nigeria, and Mali reported the largest outbreak of Ebola virus disease (EVD) in recorded history, with 28,646 documented cases, of which 11,323 were fatal [1,2]. Sierra Leone (SLE) was one of the worst affected, with 14,124 cases and 3956 fatalities [1]. Poor case management and ineffective outbreak responses in a country previously devastated by civil war, with an inadequate healthcare system, under-resourced health facilities, unsafe burial practices, and poor infection control likely fueled the epidemic.

Molecular studies revealed that the SLE portion of the outbreak, which occurred largely in isolation with concurrent outbreaks in Liberia and Guinea, originated from the introduction of two distinct lineages (SL1 and SL2) of the Makona variant of Ebola virus (EBOV) from Guinea into eastern SLE [3,4,5]. The SL3 lineage responsible for the majority of cases in SLE, evolved later in the background of SL2, and was initially characterized by a single mutation at genome position 10,218 (G>A in the untranslated region of VP24). By July 2014, the virus had reached the capital city Freetown, although sequence data is only available from this region starting in August 2014 [6]. The Western Area (WA) division of SLE is composed of two districts: the capital Freetown (henceforth referred to as WA Urban District—WAU) and WA Rural District (WAR) (Figure S1). By November 2014, the virus had diversified considerably and was co-circulating across multiple districts of SLE, with Waterloo (a transport hub located in the district of WAR) harboring the highest phylogenetic diversity [5,7].

A phylodynamic study of the entire West African outbreak, based on 1610 sequences, found that EBOV tended to disperse between areas in close geographical proximity, rather than spreading over large distances, and international transmission did not occur frequently into SLE [6]. Apart from the initial introductions, late back-and-forth movements between Kambia (SLE) and Forécariah (Guinea) helped prolong the outbreak in these areas, but these transmission chains did not spread further within SLE [6]. Repeated introductions into different geographic locations and widespread transmission in densely populated urban environments were the major contributors to the scale of the epidemic, with onward transmission to other administrative regions playing a role in the longevity of the outbreak [6]. This study; however, focused primarily on broad, regional patterns, and a comprehensive analysis of country-level dynamics has not yet been published for SLE. Furthermore, there are some important temporal and spatial gaps in the previous dataset analyzed for SLE, which we aimed to address in this study.

By August 2014, the WA had reported 84 probable or confirmed cases [1], at which time laboratory support was focused in the eastern part of the country, where the outbreak began, resulting in delayed reporting of results and possible underreporting of cases in western SLE. The WA eventually became the worst affected administrative district in SLE. The National Institute for Communicable Diseases Field Ebola Diagnostic Laboratory (NICD-FEDL), based in WAU, provided EVD diagnostic support to the severely stricken western part of SLE from August 2014 to June 2016 [8]. The NICD-FEDL operational period encompassed December 2014, a period for which there are only two published sequences (0.17% out of 1203 cases) from the WA of SLE, despite a peak in transmission during this time [1,6].

Here, we utilize additional genomes generated from samples collected at NICD-FEDL, along with all publicly available sequences from SLE, to conduct a complementary analysis of the dynamics of EBOV transmission between districts within SLE, and to test the hypothesis that administrative regions served either as sinks, sources, or hubs for virus dispersal relative to their population size and density.

## 2. Materials and Methods 

### 2.1. Samples

Clinical samples in this study were derived from 7267 diagnostic submissions, consisting of buccal swabs and blood/serum, to the NICD-FEDL between August 2014 and March 2015, operating from Lakka Hospital, Freetown [8]. Sample information and metadata are summarized in Table S1.

### 2.2. Sample Processing, Sequencing, and Genome Assembly

Samples were stored at −20 °C or −70 °C shortly after diagnostic testing. Samples were subsequently transferred on dry ice to the NICD Biosafety Level 4 facility in Johannesburg, South Africa and stored at −70 °C.

A total of 532 samples were re-tested in South Africa by an in-house qRT-PCR, targeting the polymerase L-gene, to confirm suitability for sequencing (suitability criteria established as Ct value < 25). Viral nucleic acid was extracted from serum, or swabs immersed in Eagle’s Minimum Essential Media (EMEM) culture medium (Lonza, Basel, Switzerland), using the QIAmp viral RNA kit (Qiagen, Hilden, Germany) in accordance with the manufacturer’s instructions. Host ribosomal RNA was depleted as described before [9]. Double-stranded cDNA was prepared using unbiased amplification, and sequenced on the Illumina MiSeq platform [10,11]. A subset of samples with low viral loads were enriched for EBOV nucleic acids using the TruSeq RNA Access kit (Illumina) modified with probes specific for EBOV, prior to sequencing [12].

Sequencing reads were aligned to a reference sequence (Ebola virus/H.sapiens-wt/SLE/2014/Makona-G3864.1—GenBank: KR013754) to assemble EBOV genomes. Random hexamer and adapter sequences were removed from the reads using Cutadapt v1.21 [13]. Quality filtering was performed using Prinseq-lite v0.20.4 (-lc_method dust -lc_threshold 3 -derep 14 -trim_qual_right 30 -trim_qual_type min -trim_qual_window 5 -min_qual_mean 20 -min_len 70) [14]. Reads were aligned to the reference sequence using Bowtie2 [15], duplicates were removed using Picard, and a new consensus was generated using custom scripts (https://github.com/jtladner/Scripts/tree/master/reference-based_assembly) [11]. A minimum of 10× read depth coverage, in support of the consensus, was required to make a call; positions lacking this depth were treated as missing (assigned “N”).

For 33 samples with insufficient volume for direct sequencing, virus isolates (one passage in VeroE6 cells) obtained in an earlier study were used for sequencing [16]. No evidence of cell culture-induced sequence changes, compared to clinical samples, were found in a short pilot experiment (results not shown). RNA was extracted from culture supernatant and processed as described above. Only assembled sequences with 95% or more coverage of the complete EBOV genome were included for further analyses in this study (*n* = 218).

### 2.3. Phylogenetic Analysis

A total of 1246 EBOV genomes from SLE were aligned using MAFFT v7.222 [17]. The full dataset consisted of 218 unique sequences generated in this study, and 1028 sequences available in the public domain (Table S2) [6]. Of the 1031 SLE sequences available from the Dudas et al. dataset [6], three were excluded for the following reasons: EBOV|DML13828||SLE|WesternUrban|2015-06-22—missing Genbank accession number; EBOV|KT7106||SLE|?|2015-07-19—missing Genbank accession number; and EBOV|DML25411|KT357857|SLE|Kono|2015-03-10—lack of genome coverage.

BEAST v1.8.4 [18] was used to generate a time-structured Bayesian phylogeny. This analysis included only 1062 of the EBOV genomes from SLE; 168 genomes were excluded because they lacked district-level metadata and 16 sequences were excluded because they are known to have resulted from a re-introduction from Guinea. We used an uncorrelated relaxed molecular clock with lognormally distributed rate categories [19], along with the nonparametric Bayesian SkyGrid tree prior with 50 parameters [20]. Sites were partitioned into non-coding intergenic regions and codon positions 1, 2, and 3. The evolution of all four partitions was modelled by independent HKY substitution models with Γ4-distributed rate heterogeneity. To infer viral migration between districts (within SLE), an asymmetric continuous time Markov chain (CTMC) approach was chosen [21]. Bayesian stochastic search variable selection (BSSVS) was employed to identify strongly supported (Bayes factor > 3) migrations. An uninformative reference prior was used on the migration rate. The analysis was run five independent times, each with 100 million Markov chain Monte Carlo (MCMC) steps, sampling parameters, and trees every 10,000 generations. Between 20 and 60 million MCMC steps were discarded from each run as “burn-in”, and the sampled trees were combined using Logcombiner v1.8.4.

The BEAST phylogenetic tree was visualized using Figtree v1.4.3 (http://tree.bio.ed.ac.uk/software/figtree/). Baltic (https://github.com/evogytis/baltic) was used for parsing and visualizing maps and results from BEAST trees (code available at https://github.com/jtladner/Manuscripts/tree/master/vanVuren_SLE-EBOV). For sub-lineage specific analyses, data was parsed from the trees generated from the BEAST analysis with the full data set. We defined a set of required members for each sub-lineage (Table S3), but we allowed for up to two additional, unspecified sub-lineage members per sampled tree in order to integrate over uncertainty in sub-lineage membership.

### 2.4. Statistical Analyses

Statistical analyses were performed in R and Microsoft Excel. We fit a linear model by robust regression in R v.3.4.3 using the rlm function contained within the MASS package v.7.3-47. The predict function was then used to construct 95% prediction intervals. Correlation factors (R^2^) and p-values (one-tailed distribution *t*-test with two-sample unequal variance) were determined in Excel.

### 2.5. Ethical Statement

Permission for this study was provided by the Government of Sierra Leone, Office of the Sierra Leone Ethics and Scientific Review Committee, Directorate of Training and Research, Ministry of Health and Sanitation (version 12/10/2015, approved 30/10/2015). Clearance for the export of samples from Sierra Leone to South Africa was granted under export permit numbers PBSL/061/02/2015 and PBSL/063/02/2015 by the Pharmacy Board of Sierra Leone.

## 3. Results

### 3.1. Correlation Between Cases and Population Size

We observed a strong positive correlation between population size and the number of laboratory-confirmed EVD cases per district in SLE (Table S4; Figure S2) [1,22]. Based on the best-fit slope, there was an increase of one case per 333 people. We observed one outlier district, WAR, where the actual number of confirmed cases (*n* = 1381) exceeded the expected number of cases within a 95% prediction interval. This finding supports previous observations that viral transmission within the transport hub and capital of WAR, Waterloo, played an important role in the amplification of the outbreak in SLE [5].

### 3.2. Sequencing of EBOV Genomes from SLE

A total of 218 full or near full-length EBOV genomes from individual patients from the western half of SLE were generated. The sequences were primarily derived from cases reported from WAU (*n* = 109), WAR (*n* = 34), Port Loko (*n* = 33), and Bombali (*n* = 29), but also included cases from Kambia (*n* = 2), Moyamba (*n* = 1), and Tonkolili (*n* = 10). Previously available sequence data from the densely populated WAU covered only 6.03% of laboratory confirmed cases, with our additional data increasing this coverage to 10.36% (Table S4).

Sequence data generated in this study included samples collected during August 2014 to March 2015. The number of sequences generated in this study from WA per month of the outbreak is summarized in Table S5. We provide additional sequence data from WAR and WAU from August 2014 (*n* = 7), September 2014 (*n* = 9), October 2014 (*n* = 15), November 2014 (*n* = 38), December 2014 (*n* = 45), January 2015 (*n* = 18), February 2015 (*n* = 9), and March 2015 (*n* = 2). Our additional sequence data increases the percentage of confirmed cases sequenced from WAU as follows: 1.64% to 8.2% in August 2014; 1.72% to 4.6% in September 2014; 11.1% to 14.04% in October 2014; 2.8% to 6.29% in November 2014; 0.16% to 6.45% in December 2014; 10.78% to 15.27% in January 2015; 22.39% to 29.1% in February 2015; 16.9% to 19.72% in March 2015. November to December 2014 was the peak period of virus transmission in the Western Area [1,6], particularly in WAU (Figure S3). However, previously available sequence data for December 2014 from this part of SLE amounted to a single sequence from WAU, and one specified only as being from WA. Our data have increased the available number of sequences for this period 23-fold (Table S5). 

### 3.3. Districts as Sources, Sinks, or Hubs

**Figure 1:**
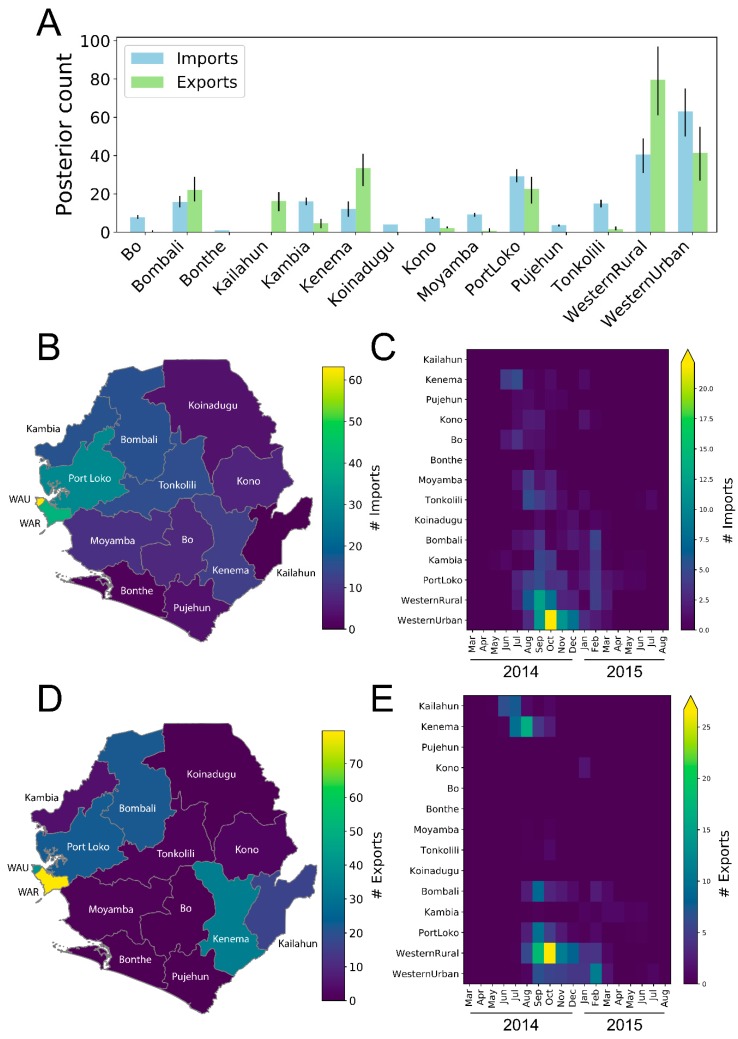
Sink/source dynamics of Ebola virus disease (EVD) in Sierra Leone based on 5 BEAST runs and >30,000 sampled trees. (**A**) Import/export events per district (means with error bars indicating 95 highest posterior density (HPD); mean of 225 total between district moves per tree, 95 HPD: 212–238); (**B**) heat map showing mean import events per district; (**C**) heat map showing mean import events over time per district; (**D**) heat map showing mean export events per district; and (**E**) heat map showing mean export events over time per district.

Using BEAST, we inferred the most likely district within SLE for each ancestral node in the phylogeny, and these reconstructions were used to infer the number of import and export events for each of the 14 districts (Table 1 and Figure 1). Overall, we detected widespread movement of EBOV within SLE, with an average of 225 movement events per sampled tree [95% highest posterior density interval (HPD95): 212–238]. This equates to approximately one inter-district movement event for every 4.7 sampled genomes. We also observed strong positive correlations between the number of imports and exports with the total number of laboratory-confirmed cases per district (Figure S4a,b). Based on the slopes from regression analyses, there was, on average, at least one importation event per 43.5 confirmed cases, and at least one exportation event per 54.3 confirmed cases. Bombali, Kono, Port Loko, Bo, and Kenema districts all exhibited higher than average number of cases per import event, consistent with the maintenance of longer than average transmission chains. Whereas, Kambia, Moyamba, and Pujehun exhibited lower than average number of cases per import event. Kailahun stood out as a statistically significant outlier with 658 EVD cases, but zero import events from within Sierra Leone. As expected, based on the positive correlation between district population size and number of EVD cases, there was also a positive correlation between the number of import events and district population size or density (R^2^ = 0.53 and 0.64 respectively) and a weak correlation between number of export events and district population size or density (R^2^ = 0.18 and 0.13 respectively).

Source-sink dynamics have been used before to describe maintenance of human influenza A virus between tropic and temperate regions [23]. The source-sink model infers that there is continuous gene flow between two populations or locations, where export/import ratios can be used to characterize these as sources or sinks, or neutral where that ratio is close to one. In the context of our analysis, we defined sources as those districts from which only export events occurred; sinks as those districts where mostly import events occurred (export to import ratio of < 0.5); and hubs as those districts where significant import and export events occurred. Four SLE districts acted only as sinks, with no exportation events (Bo, Bonthe, Koinadugu, Pujehun); four districts acted mainly as sinks but some exportation events were detected (Kambia, Kono, Moyamba, Tonkolili); five districts acted as “hubs”, with a substantial number of both imports and exports (Bombali, Kenema, Port Loko, WAR, WAU; and one district, Kailahun, acted only as a source with no importation events (from within Sierra Leone) detected. Geospatially, the districts in the southeast and northwest were the main sources or hubs for spread (Figure 1D), early and late in the outbreak, respectively (Figure 1E). EBOV was moved throughout SLE, but the high-density districts in the northwest of the country were the primary destinations for introductions. (Figure 1B, Figure S1).

**Figure 2:**
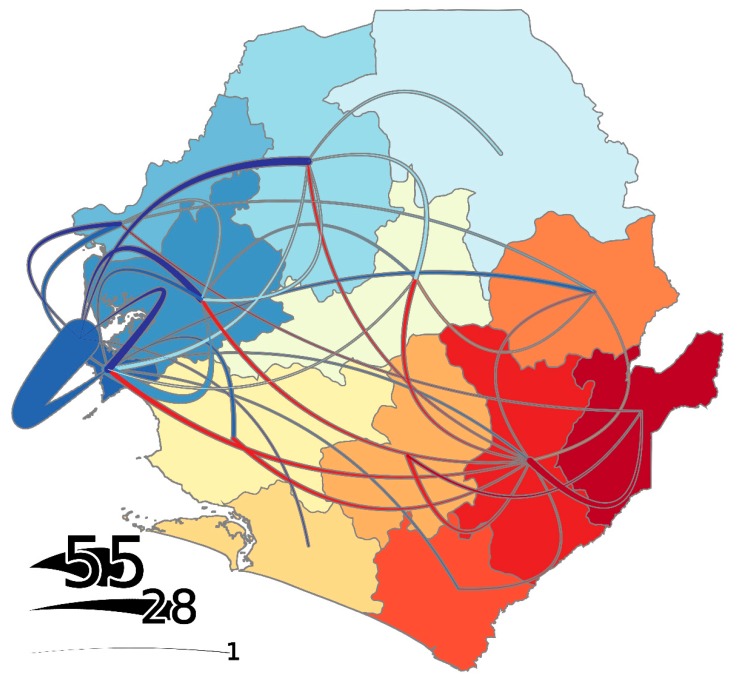
Phylogeographic BEAST reconstruction of transmission of Ebola virus in Sierra Leone. Colors of the lines correspond to the destination of the transmission event, while upward curving lines indicate eastward transmission and downward curving lines indicate westward transmission. The numbers represent the scale relative to the size of the lines.

The southeastern districts of Kailahun and Kenema acted as the major sources of dispersal during the early stages of the outbreak (Figure 1E). In fact, more than 75% of the inter-district movement events prior to September 2014 can be traced back to one of these two districts. Kailahun is where EBOV first entered from Guinea [3] and, based on regression analysis, this district stood out as a low import outlier relative to the number of EVD cases (Figure S4a). This suggests that the relatively large number of cases in Kailahun was driven primarily by initial importations from outside of SLE, followed by extensive transmission within the district, as opposed to within-country movement from other districts. As expected, based on previous studies [3], Kailahun’s exportations to other SLE districts occurred the earliest, starting in June, with the last exportation occurring in October 2014 (Figure 1E). From Kailahun, the virus dispersed mostly to neighboring Kenema, but also to more distant districts, such as Bo and Kambia, to a lesser extent (Figure 2). 

Kenema also served as an important source for viral spread early in the outbreak, resulting in introductions of the virus into at least eight SLE districts (Kono, Pujehun, Bo, Bombali, Port Loko, Tonkolili, Moyamba, and WAR) between July and October 2014 (Figure 1 and Figure 2). In fact, our regression analysis suggests that viral transmission within Kenema resulted in a significantly larger number of export events than expected based on the number of reported EVD cases (Figure S4b). Therefore, Kenema can be regarded as an important early hub for EBOV movement in SLE, facilitating transmission between Kailahun and the rest of SLE early in the outbreak. Although most importations into Kenema occurred early in the outbreak from Kailahun, there were two late re-introduction events from WAR (October 2014) and Kono (January 2015) as the virus moved back east (Figure 2). 

Later in the outbreak, as the incidence rate in the eastern portion of the country decreased (Figure S3), viral transmission within four high density northwestern districts, Bombali, Port Loko, WAR, and WAU, was primarily responsible for amplification and spread of the outbreak within SLE (Figure 1). In fact, from September 2014 to March 2015, more than 90% of all inter-district movement events originated in one of these four districts. Approximately 70% of all inter-district movement events involved the Western Area Division (WAR + WAU); however, 29% of all movement events occurred between WAU and WAR. Discounting the within-WA moves, ~59% of all other inter-district movement events involved WA as either the source or the sink. 

During the peak period of EBOV transmission in SLE, WAR acted as the main hub for the spread of the virus throughout the country (Figure 1D,E). In fact, regression analysis revealed that significantly more export events occurred from WAR than expected based on the number of confirmed EVD cases (Figure S4b). Notably; however, nearly 70% of the exportations from WAR were into WAU (Figure 2). Waterloo city in WAR is the country’s major transport hub, with major highways converging from other districts of SLE, thus serving as a gateway to and from the capital [5]. 

The role of WAU as an important hub for virus dispersal is not surprising, considering the city’s capital status, high population density, and better access to healthcare facilities. Importations into WAU occurred as early as July 2014, with a peak in October, and these importations continued at least until March/April 2015 (Figure 1C). The timing of the earliest peak of introductions into WAU (October 2014) suggests that high case numbers during the peak period of transmission (November/December 2014) was the outcome of extensive intra-district virus transmission following earlier introductions, rather than a direct effect of repeated introductions at that time. Late in the outbreak, in early 2015, the WAU was the main source for virus dispersal in SLE despite a decreased number of introductions; therefore, this region appears to have played an important role in prolonging the SLE portion of the outbreak (Figure 1E). Excluding within-WA movements, the WAU served as a source for cases in four districts: Kambia, Port Loko, Bombali, and Bonthe.

Bombali and Port Loko both served as hubs for virus dispersal (Figure 1). Both experienced importation events over extended periods during the outbreak (Bombali: July 2014 to February 2015; Port Loko: July 2014 to June 2015). Interestingly, very little transmission seemingly occurred between these two neighboring districts (Figure 2). Although the first laboratory confirmed case of EVD in Bombali was detected in July 2014, a dedicated laboratory and Ebola Treatment Unit was only established in the district in December 2014 [24]. Prior to this, patient samples were being tested by laboratories in other districts of SLE. Time from sample collection to laboratory result decreased from 2.5 to 1.2 days before and after establishment of the ETU and laboratory, leading to a decrease in time from symptom onset to laboratory result from 7.3 to 5.2 days, and a subsequent sharp decrease in EVD incidence from December onwards [24]. Analysis of a database of samples submitted to NICD FEDL for EVD testing (including but not limited to the samples for which sequences were generated in this study), for which the relevant metadata was available, revealed that the average time from patient sample collection in Bombali to laboratory testing at the NICD laboratory in Freetown was 3.0 days (*n* = 288 submissions), compared to the overall average for all districts of 1.44 days (*n* = 2984 submissions). This difference was statistically significant (*p* < 0.001). The observations by Gleason et al. [24] and our data suggest that a delayed public health response in Bombali might have contributed to within-district transmission and, hence, its establishment as a hub for virus dispersal. 

Although Kambia primarily served as a sink for EBOV transmission chains, this district played an important role during the tail end of the outbreak in SLE. More than 50% of the inter-district movement events after March 2015 were inferred to have originated in Kambia District. This included multiple re-introductions to the neighboring Port Loko District (Figure 2).

### 3.4. Temporal and Geographical Distribution of Lineages in SLE

**Figure 3:**
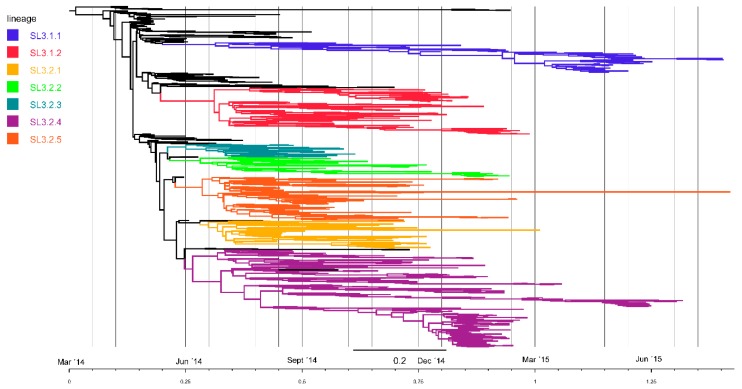
Phylogeny based on BEAST analysis of 1062 sequences from Sierra Leone (SLE). Lineages SL3.1.1, SL3.1.2, and SL3.2.1–3.2.5 are highlighted, as indicated in the legend. A time scale below the tree shows the time from the root of the tree, in years, and corresponding calendar dates.

The SLE EVD outbreak was dominated by seven primary sub-lineages (i.e., distinct transmission chains), all of which originated relativey early in the outbreak (between mid-July and early August 2014), and all of which circulated for at least four months (Figure 3; Figures S5–S8; Table S6). Each of these sub-lineages are defined by one or more basal substitution events (Table S7), though in rare cases sub-lineage members were found to lack these basal substitutions, either as the result of homoplasy or genome assembly errors. Lineage numbers were assigned according to the previously identified lineage nomenclature: SL1, SL2, SL3.1 (SL3.1.1 and 3.1.2), and SL3.2 (SL3.2.1 to 3.2.5) [5]. Although there is, as of yet, no proof of phenotypic effects of these nucleotide substitutions, grouping in clades or lineages, which indicates genetic relatedness, is useful for inferring transmission chains.

**Figure 4:**
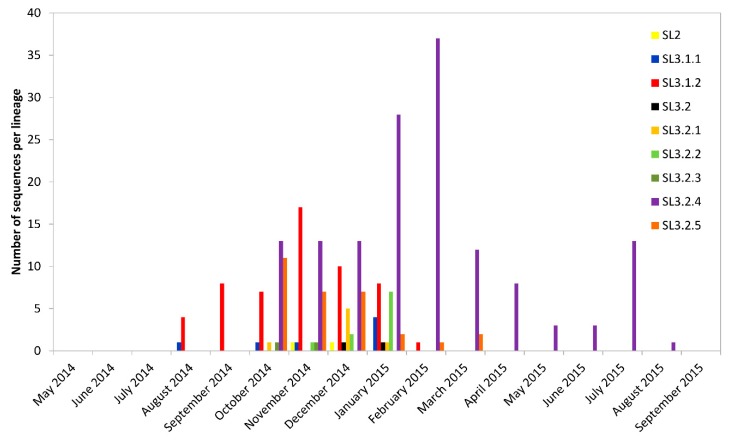
Distribution of lineages in Western Area Urban (WAU) over time (259 sequences).

The most recent common ancestor (TMRCA) for the most abundant SLE sub-lineage (SL3.2.4; TMRCA date median 16 July 2014), which accounted for 373 (29.94%) of the total number of characterized virus genomes, most likely occurred in Kenema district, and transmission was sustained over a long period (median length 383.27 days) (Table S6). Likewise, the most common recent ancestor for SL3.2.5 (TMRCA date median 23 July 2014; median length 412.96 days), SL3.2.2 (TMRCA date median 23 July 2014; median length 240.83 days), SL3.2.3 (TMRCA date median 12 July 2014; median length 132.47 days), and SL3.1.1 (TMRCA date median 6 August 2014; median length 396.26 days) likely occurred in Kenema. A low probability (20.7%) was found for SL3.1.1 to have originated in the WAR. Although SL3.1.1 accounted for only 8.11% of sequenced genomes, it was sustained for the second longest period. Transmission was sustained until September 2015, with all lineage SL3.1.1 sequences in August and September 2015 detected in Kambia. Viruses from this lineage were likely responsible for back-and-forth transmission between Kambia (SLE) and Forécariah (Guinea) [6]. Only SL3.2.5 was sustained over a longer period than SL3.1.1, but this was due to detection of a single SL3.2.5 genome in September 2015, despite the last genome from this lineage, prior to that, being detected in March of the same year. This outlier genome from September 2015 (KU296454–Bombali) was closest related to a genome last detected in November 2014 (KR65322–Bombali), and might represent a case of sexual transmission. This statement warrants not only a deeper investigation into the evolution rate of this particular genome compared to expected rate, but also into the accuracy of the metadata available for this patient. The most recent common ancestor for SL3.1.2 (TMRCA date median 3 August 2014; median length 246.34 days); however, likely occurred in the Western Area, while SL3.2.1 (TMRCA date median 30 July 2014; median length 258.21 days) likely originated in Bombali (Table S6). All seven sub-lineages were detected in WAU, but were not equally abundant (Figure 4). The highest diversity of sub-lineages in the capital occurred between October 2014 and January 2015, which corresponds to the most intense transmission period based on the number of laboratory-confirmed cases [1].

## 4. Discussion

EVD outbreaks before the 2013–2016 West Africa outbreak were limited to rural areas, and spread beyond affected villages was rarely reported. One of the unique features of the West Africa outbreak was the spread of EVD to densely populated urban centers, resulting in a major challenge to public health [25]. This outbreak was also characterized by extended transmission over a large geographical area, including all 14 administrative districts within SLE. We show a strong positive correlation between population size per district and number of laboratory-confirmed cases, consistent with high levels of mixing within SLE, with local population size being a primary driver of case numbers. This observation is to be expected for a well-distributed outbreak [6]. 

The wealth of publicly available viral genome sequences and associated metadata, along with the new sequences we generated, offered us the opportunity to perform a comprehensive, descriptive analysis of the SLE portion of the outbreak, which is complimentary to previous analyses that have either focused only on portions of SLE or on West Africa as a whole. In particular, we provide additional sequences from a peak transmission period in the Western Area, for which very little sequence data were available previously. We evaluated the role of different administrative districts as sources, sinks, or hubs for virus dispersal. 

Six districts were major sources or hubs for virus dispersal within SLE. Dispersal from districts in the southeast of the country occurred early in the outbreak, after which the hubs in the northwest were responsible for further maintenance and perpetuation of the outbreak. Understanding the reasons why these particular districts acted as hubs or sources of dispersal could be valuable for the management of future outbreaks. Kailahun clearly served as a source because this is the district where the virus was initially introduced from Guinea, and subsequently transmitted mostly to the neighboring Kenema. In turn, Kenema served as an intermediate hub between Kailahun and the rest of SLE, likely due to its geographical location but also the presence of the Kenema Government Hospital. This hospital is well known for its work on another hemorrhagic fever, Lassa, and this status likely contributed to an influx of patients from Kailahun when the outbreak started. 

The major role of the Western Area division, including WAU and WAR, is highlighted by the extensive within-WA transmission, and subsequent role of WAU in the dispersal of the virus late in the outbreak. Desire for better healthcare, attendance of burials/ceremonies, visiting family, and fleeing from stigmatization, combined with high population density may have contributed to the intense and protracted transmission of the virus into and within WAU, and its role as a hub. An influx of patients to the capital for better healthcare likely overwhelmed the health system and may have been one of the drivers of its role as a transmission hub. 

A poor and delayed public health response likely contributed to Bombali becoming a major hub for virus transmission and dispersal. During a peak of transmission of four months between July and November 2014 in Bombali, there was no local laboratory or Ebola Treatment Unit capacity [24], necessitating the testing of samples from suspected EVD cases in Freetown laboratories, and resulting in delays, on average, just below four days. Although drivers of explosive EVD transmission are certainly complex, a poor laboratory response seems to have been a contributing factor in the timely implementation of outbreak control measures. 

Interestingly, the less densely populated districts located in the northeast, interior, and south-west of SLE were almost exclusively sinks. This suggests that importations, although resulting in local transmission within the particular districts, did not result in extensive onward transmission to other districts. The sink dynamics of these rural interior districts highlights the complexity and importance of understanding human behavior in EVD dispersal. As opposed to desire for better healthcare in urban centers, this observation might be suggestive of a proportion of the rural population actively avoiding urban hospitals or control efforts.

Two recent outbreaks in Democratic Republic of Congo (DRC), in Equateur and North Kivu provinces, in 2018, raised fears of a repeat of the West Africa crisis. The most obvious similarity between these outbreaks was the relatively early introduction of cases into dense populated urban centers with ports and transport links to other major cities and neighboring countries [26]. However, the difference in the public health response was glaring, in terms of speed and approach, mostly due to hard lessons learned in West Africa, and eventually the Equateur Province outbreak was rapidly contained [27,28,29]. In addition to traditional public health measures during EVD outbreaks, the 2018 outbreaks were characterized by the early introduction of the experimental rVSV-ZEBOV vaccine for ring vaccination [26,30,31].

At the time of writing, the North Kivu EVD outbreak was still ongoing. On the 4th of September 2018, less than a month after the declaration of the outbreak, the first EVD death was recorded in the DRC city of Butembo [32]. Butembo, with an estimated population of more than one million, is an important commercial center, with an airport and large markets. Rapid mobilization of traditional public health measures, combined with new technologies, such as point-of-care testing and experimental vaccines, in affected areas that could potentially act as a source for virus dispersal, would likely represent an effective way of preventing rural–urban–rural transmission of EVD. However, even though better control measures are now available, their effective implementation is jeopardized by political unrest in the affected areas of the DRC [33].

The ultimate goal in widespread EVD outbreaks would be to skew the sink–source–hub dynamics of urban centers towards a sink role, by breaking transmission chains through traditional and next generation intervention efforts, and thereby limiting onward transmission. Our data suggest that, among other intervention efforts, reducing the time from identification of suspected cases or potential contacts in transmission chains to diagnosis could contribute to preventing highly populated urban centers from becoming virus dispersal hubs. Our data confirms that highly populated urban centers would naturally serve as sources or hubs for dispersal, but this should be minimized at all costs through prevention and control strategies. Further investigation into the factors that resulted in sink–source–hub dynamics of SLE districts could aid future intervention efforts to curb extensive geographical spread and prolonged maintenance of EVD outbreaks.

## Figures and Tables

**Table 1:** Districts in Sierra Leone as sources, sinks, or hubs.

District	Median Import Events	Median Export Events	Ratio Exports/Imports	Source/Sink/Hub	Total #Cases	Cases/Import	Cases/Export
**Bo**	8	0	0.02	Sink	358	46.31	N/A
**Bombali**	16	22	1.40	Hub	1064	67.56	48
**Bonthe**	1	0	0.00	Sink	1	1.00	N/A
**Kailahun**	0	16	N/A	Source	658	N/A	40
**Kambia**	16	5	0.29	Sink	277	17.11	58
**Kenema**	12	33	2.74	Hub	532	43.57	16
**Koinadugu**	4	0	0.00	Sink	155	38.65	N/A
**Kono**	7	2	0.30	Sink	450	62.59	210
**Moyamba**	9	1	0.08	Sink	276	29.68	394
**PortLoko**	29	23	0.77	Hub	1609	55.27	71
**Pujehun**	4	0	0.01	Sink	54	14.56	N/A
**Tonkolili**	15	2	0.10	Sink	505	33.53	326
**Western Area Rural**	41	80	1.97	Hub	1381	34.09	17
**Western Area Urban**	63	42	0.66	Hub	2520	39.92	61

## Supplementary Materials

The following are available online at http://www.mdpi.com/1999-4915/11/1/71/s1; Table S1: Metadata of genomes generated in this study. Table S2: Metadata for complete dataset, Table S3: Required members for each sub-lineage, Table S4: Geographical distribution of EVD cases and sequences used in this study. Table S5: Temporal distribution of cases and sequences from the Western Area for the period August 2014 to March 2015. Table S6: The most recent common ancestors (TMRCA) for the seven main sub-lineages, and information on duration of circulation. Table S7: Mutations defining the seven sub-lineages of SL3. Figure S1. Map of SLE showing total case counts (laboratory confirmed as per WHO situation report) per administrative district. The districts are colored according to population density, with darker colors representing more densely populated districts. (https://www.citypopulation.de/SierraLeone-Cities.html) (http://apps.who.int/ebola/current-situation/ebola-situation-report-30-march-2016). Figure S2. Correlation plots of number of laboratory-confirmed EVD cases and district population size. The solid line represents the robust regression best-fit line and the dotted lines indicate the 95% prediction intervals. Figure S3. Laboratory-confirmed cases per district over time. Figure S4. Correlation plots of imports per cases (a) and exports per cases (b). The solid line represents the robust regression best-fit line and the dotted lines indicate the 95% prediction intervals. Figure S5. Phylogeographic reconstruction of the movement of SL3.2.4 in SLE over time. Districts are each assigned a unique color and the color of the arch is according to the source district. The circle indicates the probable location of the common recent ancestor. Figure S6. Phylogeographic reconstruction of the movement of SL3.1.2 in SLE over time. Districts are each assigned a unique color and the color of the arch is according to the source district. The circle indicates the probable location of the common recent ancestor. Figure S7. Phylogeographic reconstruction of the movement of SL3.2.5 in SLE over time. Districts are each assigned a unique color and the color of the arch is according to the source district. The circle indicates the probable location of the common recent ancestor. Figure S8. Phylogeographic reconstruction of the movement of SL3.1.1 (A), SL3.2.1 (B), SL3.2.2 (C), and SL3.2.3 (D) in SLE over time. Districts are each assigned a unique color and the color of the arch is according to the source district. The circle indicates the probable location of the common recent ancestor.
