# Supplementary material for: Phylodynamic Analysis of Ebola Virus Disease Transmission in Sierra Leone
Source: Viruses. 2019 Jan 16;11(1):71. doi: 10.3390/v11010071 (PMC6356631; doi:10.3390/v11010071)
Supplement: Supplementary file 1 [file viruses-11-00071-s001.zip › supplementary/Table S1-S7.pdf]

Table S1. Metadata of genomes generated in this study

Sample ID	COLLECTION DAY	COLLECTION MONTH	COLLECTION YEAR	DISTRICT	Town/Village/Suburb	Lineage	Genbank accession number
NICD0013_SLE_WR_2014-08-26	26	8	2014	WAR	Waterloo	SL3.2.3	MH607891
NICD0019_SLE_WR_2014-08-26	26	8	2014	WAR	Waterloo	SL3.2.4	MH607892
NICD0022_SLE_WUR_2014-08-26	26	8	2014	WAU	Lumley	SL3.1.2	MH607893
NICD0023_SLE_TONK_2014-08-26	26	8	2014	Tonkolili	Magburaka	SL3.2	MH607894
NICD0031_SLE_WUR_2014-08-26	26	8	2014	WAU	Lumley	SL3.1.2	MH607895
NICD0032_SLE_MOY_2014-08-27	27	8	2014	Moyamba	Rotifunk	SL3.2.3	MH607896
NICD0034_SLE_WUR_2014-08-26	26	8	2014	WAU	Lumley	SL3.1.2	MH607897
NICD0086_SLE_BOM_2014-08-29	29	8	2014	Bombali	Makeni	SL3.2.5	MH607898
NICD0104_SLE_WR_2014-08-30	30	8	2014	WAR	Waterloo	SL3.2.2	MH607899
NICD0108_SLE_PL_2014-08-30	30	8	2014	Port Loko	Lunsar	SL3.2.2	MH607900
NICD0110_SLE_PL_2014-08-30	30	8	2014	Port Loko	Lunsar	SL3.2.3	MH607901
NICD0129_SLE_BOM_2014-08-31	31	8	2014	Bombali	Makeni	SL3.2.5	MH607902
NICD0143_SLE_WUR_2014-08-31	31	8	2014	WAU	Freetown	SL3.1.1	MH607903
NICD0188_SLE_BOM_2014-09-03	3	9	2014	Bombali	Makeni	SL3.2.5	MH607904
NICD0190_SLE_BOM_2014-09-03	3	9	2014	Bombali	Makeni	SL3.2.5	MH607905
NICD0193_SLE_BOM_2014-09-03	3	9	2014	Bombali	Makeni	SL3.2.5	MH607906

NICD0234_SLE_PL_2014-09-04	4	9	2014	Port Loko	Feredugu	SL3.2.5	MH607907
NICD0235_SLE_PL_2014-09-04	4	9	2014	Port Loko	Lokomasama	SL3.2.5	MH607908
NICD0256_SLE_BOM_2014-09-04	4	9	2014	Bombali	Makeni	SL3.2.5	MH607909
NICD0257_SLE_BOM_2014-09-05	5	9	2014	Bombali	Makeni	SL3.2.5	MH607910
NICD0264_SLE_BOM_2014-09-05	5	9	2014	Bombali	Makeni	SL3.2.5	MH607911
NICD0298_SLE_PL_2014-09-05	5	9	2014	Port Loko	Lunsar	SL3.2.3	MH607912
NICD0299_SLE_PL_2014-09-05	5	9	2014	Port Loko	Buya Romende	SL3.2.5	MH607913
NICD0338_SLE_PL_2014-09-08	8	9	2014	Port Loko	Port Loko	SL3.2.2	MH607914
NICD0369_SLE_BOM_2014-09-07	7	9	2014	Bombali	Makeni	SL3.2.5	MH607915
NICD0393-Vero_SLE_WUR_2014-09-09	9	9	2014	WAU	Freetown	SL3.1.2	MH607916
NICD0424_SLE_TONK_2014-09-10	10	9	2014	Tonkolili	Magburaka	SL3.2.2	MH607917
NICD0520_SLE_BOM_2014-09-11	11	9	2014	Bombali	Makeni	SL3.2	MH607918
NICD0637_SLE_TONK_2014-09-16	16	9	2014	Tonkolili	Yele	SL3.2.5	MH607919
NICD0650_SLE_BOM_2014-09-15	15	9	2014	Bombali	Makeni	SL3.2.5	MH607920
NICD0689_SLE_TONK_2014-09-17	17	9	2014	Tonkolili	Magburaka	SL3.2.1	MH607921
NICD0720-Vero_SLE_PL_2014-09-16	16	9	2014	Port Loko	Lunsar	SL3.2.2	MH607922
NICD0721_SLE_PL_2014-09-16	16	9	2014	Port Loko	Lunsar	SL3.2.2	MH607923
NICD0782_SLE_BOM_2014-09-17	17	9	2014	Bombali	Makeni	SL3.2.5	MH607924
NICD0787_SLE_BOM_2014-09-17	17	9	2014	Bombali	Makeni	SL3.2.1	MH607925

NICD0821_SLE_WR_2014-09-20	20	9	2014	WAR	Waterloo	SL3.1.2	MH607926
NICD0856-Vero_SLE_WR_2014-09-20	20	9	2014	WAR	Waterloo	SL3.2.3	MH607927
NICD0878_SLE_PL_2014-09-20	20	9	2014	Port Loko	Lunsar	SL3.2.2	MH607928
NICD0894_SLE_PL_2014-09-20	20	9	2014	Port Loko	Lunsar	SL3.2.2	MH607929
NICD0899_SLE_PL_2014-09-20	20	9	2014	Port Loko	Masuba	SL3.2.2	MH607930
NICD0900_SLE_PL_2014-09-20	20	9	2014	Port Loko	Lunsar	SL3.2.2	MH607931
NICD0905_SLE_TONK_2014-09-20	20	9	2014	Tonkolili	Magburaka	SL3.2.1	MH607932
NICD0908_SLE_TONK_2014-09-20	20	9	2014	Tonkolili	Magburaka	SL3.2.1	MH607933
NICD0911_SLE_TONK_2014-09-20	20	9	2014	Tonkolili	Magburaka	SL3.2.1	MH607934
NICD0919_SLE_TONK_2014-09-19	19	9	2014	Tonkolili	Magburaka	SL3.2.5	MH607935
NICD0964_SLE_TONK_2014-09-21	21	9	2014	Tonkolili	Magburaka	SL3.2.1	MH607936
NICD0983_SLE_WUR_2014-09-22	22	9	2014	WAU	Freetown	SL3.1.2	MH607937
NICD0986-Vero_SLE_WUR_2014-09-22	22	9	2014	WAU	Freetown	SL3.1.2	MH607938
NICD1016_SLE_PL_2014-09-21	21	9	2014	Port Loko	Buya Romende	SL3.2.5	MH607939
NICD1043_SLE_PL_2014-09-22	22	9	2014	Port Loko	Port Loko	SL3.2.3	MH607940
NICD1049_SLE_PL_2014-09-21	21	9	2014	Port Loko	Port Loko	SL3.2.2	MH607941
NICD1051_SLE_PL_2014-09-21	21	9	2014	Port Loko	Lungi	SL3.2.3	MH607942
NICD1079_SLE_BOM_2014-09-20	20	9	2014	Bombali	Makeni	SL3.2.5	MH607943
NICD1081_SLE_BOM_2014-09-20	20	9	2014	Bombali	Makeni	SL3.2.1	MH607944

NICD1089-Vero_SLE_BOM_2014-09-20	20	9	2014	Bombali	Makeni	SL3.2.5	MH607945
NICD1091_SLE_BOM_2014-09-20	20	9	2014	Bombali	Makeni	SL3.2.1	MH607946
NICD1092_SLE_BOM_2014-09-20	20	9	2014	Bombali	Makeni	SL3.2.1	MH607947
NICD1100_SLE_BOM_2014-09-21	21	9	2014	Bombali	Makeni	SL3.2.5	MH607948
NICD1105_SLE_BOM_2014-09-21	21	9	2014	Bombali	Makeni	SL3.2.1	MH607949
NICD1109_SLE_BOM_2014-09-19	19	9	2014	Bombali	Makeni	SL3.2.1	MH607950
NICD1113_SLE_BOM_2014-09-19	19	9	2014	Bombali	Makeni	SL3.1.2	MH607951
NICD1136_SLE_WR_2014-09-23	23	9	2014	WAR	Waterloo	SL3.2.1	MH607952
NICD1142_SLE_PL_2014-09-22	22	9	2014	Port Loko	Mamamah (Mamana)	SL3.2.1	MH607953
NICD1143_SLE_PL_2014-09-23	23	9	2014	Port Loko	Port Loko	SL3.2.1	MH607954
NICD1147_SLE_PL_2014-09-22	22	9	2014	Port Loko	Port Loko	SL3.2.5	MH607955
NICD1148_SLE_PL_2014-09-22	22	9	2014	Port Loko	Port Loko	SL3.2.5	MH607956
NICD1156_SLE_PL_2014-09-22	22	9	2014	Port Loko	Kalangbani	SL3.2.5	MH607957
NICD1167_SLE_PL_2014-09-22	22	9	2014	Port Loko	Port Loko	SL3.2.5	MH607958
NICD1201_SLE_TONK_2014-09-20	20	9	2014	Tonkolili	Yonibana	SL3.2.5	MH607959
NICD1209-Vero_SLE_WUR_2014-09-23	23	9	2014	WAU	Freetown	SL3.1.2	MH607960
NICD1227_SLE_BOM_2014-09-22	22	9	2014	Bombali	Makeni	SL3.2.1	MH607961
NICD1256-Vero_SLE_BOM_2014-09-22	22	9	2014	Bombali	Makeni	SL3.2.1	MH607962
NICD1261_SLE_BOM_2014-09-22	22	9	2014	Bombali	Makeni	SL3.2.1	MH607963

NICD1272-Vero_SLE_BOM_2014-09-22	22	9	2014	Bombali	Makeni	SL3.2.1	MH607964
NICD1275_SLE_WR_2014-09-25	25	9	2014	WAR	Waterloo	SL3.1.1	MH607965
NICD1317_SLE_BOM_2014-09-24	24	9	2014	Bombali	Makeni	SL3.2.5	MH607966
NICD1329-Vero_SLE_BOM_2014-09-24	24	9	2014	Bombali	Makeni	SL3.2.1	MH607967
NICD1396_SLE_PL_2014-09-25	25	9	2014	Port Loko	Port Loko	SL3.2.2	MH607968
NICD1422_SLE_PL_2014-09-26	26	9	2014	Port Loko	Port Loko	SL3.2.5	MH607969
NICD1430_SLE_PL_2014-09-26	26	9	2014	Port Loko	Port Loko	SL3.2.5	MH607970
NICD1458_SLE_WUR_2014-09-29	29	9	2014	WAU	Freetown	SL3.1.2	MH607971
NICD1514_SLE_PL_2014-09-28	28	9	2014	Port Loko	Port Loko	SL3.2.5	MH607972
NICD1526_SLE_WR_2014-10-01	1	10	2014	WAR	Waterloo	SL3.2.4	MH607973
NICD1662-Vero_SLE_BOM_2014-10-01	1	10	2014	Bombali	Makeni	SL3.2.5	MH607974
NICD1972-Vero_SLE_WUR_2014-10-09	9	10	2014	WAU	Freetown	SL3.2.4	MH607975
NICD2031-Vero_SLE_WUR_2014-10-11	11	10	2014	WAU	Freetown	SL3.1.2	MH607976
NICD2032-Vero_SLE_WUR_2014-10-11	11	10	2014	WAU	Freetown	SL3.1.2	MH607977
NICD2118_SLE_WUR_2014-10-11	11	10	2014	WAU	Freetown	SL3.2.4	MH607978
NICD2219_SLE_WUR_2014-10-14	14	10	2014	WAU	Freetown	SL3.2.5	MH607979
NICD2308_SLE_WR_2014-10-16	16	10	2014	WAR	Goderich	SL3.2.5	MH607980
NICD2310_SLE_WR_2014-10-16	16	10	2014	WAR	Goderich	SL3.2.5	MH607981
NICD2494_SLE_WR_2014-10-23	23	10	2014	WAR	Goderich	SL3.2.5	MH607982

NICD2521-swab_SLE_WUR_2014-10-23	23	10	2014	WAU	Freetown	SL3.2.3	MH607983
NICD2538_SLE_WUR_2014-10-22	22	10	2014	WAU	Freetown	SL3.1.2	MH607984
NICD2540-Vero_SLE_WUR_2014-10-22	22	10	2014	WAU	Freetown	SL3.2.4	MH607985
NICD2541_SLE_WUR_2014-10-22	22	10	2014	WAU	Freetown	SL3.1.2	MH607986
NICD2542_SLE_WUR_2014-10-22	22	10	2014	WAU	Freetown	SL3.2.4	MH607987
NICD2589_SLE_WR_2014-10-24	24	10	2014	WAR	Waterloo	SL3.2.5	MH607988
NICD2748_SLE_KMB_2014-11-06	6	11	2014	Kambia	Mambolo	SL3.1.2	MH607989
NICD2749_SLE_KMB_2014-11-06	6	11	2014	Kambia	Kambia	SL3.2.4	MH607990
NICD2763-Vero_SLE_PL_2014-11-06	6	11	2014	Port Loko	Mabamba	SL3.1.1	MH607991
NICD2965-Vero_SLE_PL_2014-11-10	10	11	2014	Port Loko	Lunsar	SL3.2.3	MH607992
NICD2974-swab_SLE_PL_2014-11-08	8	11	2014	Port Loko	Makare	SL3.2.2	MH607993
NICD3031_SLE_PL_2014-11-11	11	11	2014	Port Loko	Port Loko	SL3.1.2	MH607994
NICD3087_SLE_WUR_2014-11-13	13	11	2014	WAU	Freetown	SL3.1.2	MH607995
NICD3089_SLE_PL_2014-11-12	12	11	2014	Port Loko	Port Loko	SL3.1.2	MH607996
NICD3097_SLE_PL_2014-11-12	12	11	2014	Port Loko	Masiaka	SL3.2.2	MH607997
NICD3208-Vero_SLE_WR_2014-11-14	14	11	2014	WAR	Goderich	SL3.2.1	MH607998
NICD3294_SLE_WR_2014-11-14	14	11	2014	WAR	Goderich	SL3.2.1	MH607999
NICD3304-swab_SLE_WUR_2014-11-15	15	11	2014	WAU	Freetown	SL3.2.5	MH608000
NICD3335_SLE_WR_2014-11-16	16	11	2014	WAR	Waterloo	SL3.1.2	MH608001

NICD3341_SLE_WUR_2014-11-16	16	11	2014	WAU	Freetown	SL3.2.5	MH608002
NICD3420_SLE_WUR_2014-11-17	17	11	2014	WAU	Freetown	SL3.2.5	MH608003
NICD3465-swab_SLE_WUR_2014-11-18	18	11	2014	WAU	Freetown	SL3.2.4	MH608004
NICD3483-Vero_SLE_WUR_2014-11-18	18	11	2014	WAU	Freetown	SL3.1.2	MH608005
NICD3577_SLE_WR_2014-11-20	20	11	2014	WAR	Waterloo	SL3.1.1	MH608006
NICD3596_SLE_WUR_2014-11-21	21	11	2014	WAU	Freetown	SL3.1.2	MH608007
NICD3608-Vero_SLE_WR_2014-11-21	21	11	2014	WAR	Goderich	SL3.2.1	MH608008
NICD3666_SLE_WR_2014-11-22	22	11	2014	WAR	Waterloo	SL3.2.4	MH608009
NICD3670_SLE_WR_2014-11-22	22	11	2014	WAR	Rokel	SL3.2.3	MH608010
NICD3687-Vero_SLE_WUR_2014-11-23	23	11	2014	WAU	Freetown	SL3.2.4	MH608011
NICD3693-Vero_SLE_WR_2014-11-23	23	11	2014	WAR	Goderich	SL3.2.1	MH608012
NICD3707_SLE_WUR_2014-11-24	24	11	2014	WAU	Freetown	SL3.1.2	MH608013
NICD3752_SLE_WR_2014-11-24	24	11	2014	WAR	Waterloo	SL3.2.4	MH608014
NICD3753_SLE_WR_2014-11-24	24	11	2014	WAR	Goderich	SL3.2.4	MH608015
NICD3820_SLE_WUR_2014-11-25	25	11	2014	WAU	Freetown	SL3.1.2	MH608016
NICD3823_SLE_WR_2014-11-25	25	11	2014	WAR	Rokel	SL3.1.2	MH608017
NICD3824_SLE_WR_2014-11-25	25	11	2014	WAR	Goderich	SL3.2.4	MH608018
NICD3925_SLE_WUR_2014-11-27	27	11	2014	WAU	Freetown	SL3.1.2	MH608019
NICD3926_SLE_WUR_2014-11-27	27	11	2014	WAU	Freetown	SL3.1.1	MH608020

NICD3928_SLE_WUR_2014-11-26	26	11	2014	WAU	Freetown	SL3.1.2	MH608021
NICD3932_SLE_WUR_2014-11-26	26	11	2014	WAU	Freetown	SL3.1.2	MH608022
NICD3937_SLE_WUR_2014-11-26	26	11	2014	WAU	Freetown	SL3.1.2	MH608023
NICD3944_SLE_WUR_2014-11-26	26	11	2014	WAU	Freetown	SL3.2.4	MH608024
NICD3954_SLE_WUR_2014-11-26	26	11	2014	WAU	Freetown	SL3.2.4	MH608025
NICD4024-Vero_SLE_WUR_2014-11-29	29	11	2014	WAU	Freetown	SL3.2.4	MH608026
NICD4025-Vero_SLE_WUR_2014-11-29	29	11	2014	WAU	Freetown	SL3.1.2	MH608027
NICD4027-Vero_SLE_WUR_2014-11-29	29	11	2014	WAU	Freetown	SL3.2.4	MH608028
NICD4029-Vero_SLE_WUR_2014-11-29	29	11	2014	WAU	Freetown	SL3.2.4	MH608029
NICD4030-Vero_SLE_WR_2014-11-29	29	11	2014	WAR	Goderich	SL3.2.5	MH608030
NICD4041-Vero_SLE_WUR_2014-11-29	29	11	2014	WAU	Lumley	SL3.2.4	MH608031
NICD4055_SLE_WUR_2014-11-28	28	11	2014	WAU	Freetown	SL3.1.2	MH608032
NICD4064-swab_SLE_WUR_2014-11-29	29	11	2014	WAU	Freetown	SL3.1.2	MH608033
NICD4072-swab_SLE_WUR_2014-11-30	30	11	2014	WAU	Freetown	SL3.2.5	MH608034
NICD4113_SLE_WUR_2014-12-01	1	12	2014	WAU	Freetown	SL3.2.5	MH608035
NICD4122_SLE_WR_2014-12-02	2	12	2014	WAR	Songo	SL3.2.4	MH608036
NICD4125_SLE_WR_2014-12-02	2	12	2014	WAR	Hill Station	SL3.2.4	MH608037
NICD4167_SLE_WUR_2014-12-02	2	12	2014	WAU	Freetown	SL3.2.4	MH608038
NICD4279-Vero_SLE_WUR_2014-12-05	5	12	2014	WAU	Freetown	SL3.1.2	MH608039

NICD4325_SLE_WR_2014-12-05	5	12	2014	WAR	Waterloo	SL3.2.4	MH608040
NICD4329_SLE_WUR_2014-12-05	5	12	2014	WAU	Freetown	SL3.2.5	MH608041
NICD4356_SLE_WUR_2014-12-06	6	12	2014	WAU	Freetown	SL3.2.5	MH608042
NICD4361-Vero_SLE_WR_2014-12-06	6	12	2014	WAR	Waterloo	SL3.2.4	MH608043
NICD4430_SLE_WUR_2014-12-06	6	12	2014	WAU	Freetown	SL3.2.4	MH608044
NICD4513-Vero_SLE_WUR_2014-12-09	9	12	2014	WAU	Freetown	SL3.2.1	MH608045
NICD4670_SLE_WUR_2014-12-12	12	12	2014	WAU	Freetown	SL3.2.1	MH608046
NICD4672_SLE_WUR_2014-12-12	12	12	2014	WAU	Freetown	SL3.2.1	MH608047
NICD4674_SLE_WR_2014-12-12	12	12	2014	WAR	Waterloo	SL3.2.4	MH608048
NICD4682_SLE_WUR_2014-12-13	13	12	2014	WAU	Freetown	SL3.1.2	MH608049
NICD4691_SLE_WUR_2014-12-11	11	12	2014	WAU	Freetown	SL3.2.1	MH608050
NICD4692_SLE_WUR_2014-12-13	13	12	2014	WAU	Freetown	SL3.2.1	MH608051
NICD4693_SLE_WUR_2014-12-10	10	12	2014	WAU	Freetown	SL3.2.4	MH608052
NICD4735_SLE_WUR_2014-12-13	13	12	2014	WAU	Freetown	SL3.2.4	MH608053
NICD4743_SLE_WR_2014-12-12	12	12	2014	WAR	Tombo	SL2	MH608054
NICD4795-swab_SLE_WUR_2014-12-14	14	12	2014	WAU	Freetown	SL3.2.2	MH608055
NICD4823_SLE_WUR_2014-12-15	15	12	2014	WAU	Freetown	SL3.2.4	MH608056
NICD4830_SLE_WUR_2014-12-15	15	12	2014	WAU	Freetown	SL3.1.2	MH608057
NICD4877_SLE_WUR_2014-12-16	16	12	2014	WAU	Freetown	SL3.2.4	MH608058

NICD4932-Vero_SLE_WUR_2014-12-17	17	12	2014	WAU	Freetown	SL3.2.4	MH608059
NICD4975_SLE_WUR_2014-12-18	18	12	2014	WAU	Freetown	SL3.2.4	MH608060
NICD5071_SLE_WUR_2014-12-20	20	12	2014	WAU	Freetown	SL3.2.4	MH608061
NICD5154_SLE_WUR_2014-12-23	23	12	2014	WAU	Freetown	SL2	MH608062
NICD5200_SLE_WUR_2014-12-24	24	12	2014	WAU	Freetown	SL3.1.2	MH608063
NICD5260_SLE_WUR_2014-12-24	24	12	2014	WAU	Freetown	SL3.1.2	MH608064
NICD5266_SLE_WUR_2014-12-24	24	12	2014	WAU	Freetown	SL3.1.2	MH608065
NICD5279-swab_SLE_WUR_2014-12-24	24	12	2014	WAU	Freetown	SL3.2	MH608066
NICD5291_SLE_WUR_2014-12-25	25	12	2014	WAU	Freetown	SL3.2.5	MH608067
NICD5293_SLE_WUR_2014-12-25	25	12	2014	WAU	Freetown	SL3.2.5	MH608068
NICD5311_SLE_WUR_2014-12-26	26	12	2014	WAU	Freetown	SL3.2.5	MH608069
NICD5314_SLE_WUR_2014-12-26	26	12	2014	WAU	Freetown	SL3.1.2	MH608070
NICD5354_SLE_WUR_2014-12-27	27	12	2014	WAU	Freetown	SL3.1.2	MH608071
NICD5360_SLE_WUR_2014-12-27	27	12	2014	WAU	Freetown	SL3.1.2	MH608072
NICD5367-swab_SLE_WUR_2014-12-27	27	12	2014	WAU	Freetown	SL3.2.2	MH608073
NICD5370_SLE_WR_2014-12-28	28	12	2014	WAR	Goderich	SL3.2.5	MH608074
NICD5386_SLE_WUR_2014-12-29	29	12	2014	WAU	Freetown	SL3.2.4	MH608075
NICD5388_SLE_WUR_2014-12-29	29	12	2014	WAU	Freetown	SL3.2.4	MH608076
NICD5395_SLE_WUR_2014-12-29	29	12	2014	WAU	Freetown	SL3.2.4	MH608077

NICD5400_SLE_WUR_2014-12-28	28	12	2014	WAU	Freetown	SL3.2.4	MH608078
NICD5421_SLE_WUR_2014-12-30	30	12	2014	WAU	Freetown	SL3.2.5	MH608079
NICD5584_SLE_WUR_2015-01-01	1	1	2015	WAU	Freetown	SL3.1.1	MH608080
NICD5647-Vero_SLE_WUR_2015-01-03	3	1	2015	WAU	Freetown	SL3.2.4	MH608081
NICD5725_SLE_WUR_2015-01-07	7	1	2015	WAU	Freetown	SL3.1.2	MH608082
NICD5747_SLE_WUR_2015-01-08	8	1	2015	WAU	Freetown	SL3.2.4	MH608083
NICD5819_SLE_WUR_2015-01-09	9	1	2015	WAU	Freetown	SL3.2.4	MH608084
NICD6079_SLE_WUR_2015-01-17	17	1	2015	WAU	Freetown	SL3.2.2	MH608085
NICD6123_SLE_WR_2015-01-20	20	1	2015	WAR	Freetown	SL3.1.2	MH608086
NICD6141_SLE_WR_2015-01-21	21	1	2015	WAR	Freetown	SL3.1.2	MH608087
NICD6195_SLE_WUR_2015-01-23	23	1	2015	WAU	Freetown	SL3.2.4	MH608088
NICD6218_SLE_WUR_2015-01-24	24	1	2015	WAU	Freetown	SL3.1.2	MH608089
NICD6274_SLE_WUR_2015-01-26	26	1	2015	WAU	Freetown	SL3.1.2	MH608090
NICD6345_SLE_WUR_2015-01-27	27	1	2015	WAU	Freetown	SL3.2.4	MH608091
NICD6348_SLE_WUR_2015-01-28	28	1	2015	WAU	Freetown	SL3.2.4	MH608092
NICD6365_SLE_WR_2015-01-29	29	1	2015	WAR	Hamilton	SL3.1.2	MH608093
NICD6392-Vero_SLE_WUR_2015-01-30	30	1	2015	WAU	Freetown	SL3.2.4	MH608094
NICD6403_SLE_WUR_2015-01-29	29	1	2015	WAU	Freetown	SL3.2.4	MH608095
NICD6404_SLE_WUR_2015-01-29	29	1	2015	WAU	Freetown	SL3.2.4	MH608096

NICD6414_SLE_WUR_2014-01-30	30	1	2015	WAU	Freetown	SL3.2.4	MH608097
NICD6564_SLE_WUR_2015-02-07	7	2	2015	WAU	Aberdeen	SL3.2.4	MH608098
NICD6587_SLE_WUR_2015-02-10	10	2	2015	WAU	Freetown	SL3.2.4	MH608099
NICD6601_SLE_WUR_2015-02-11	11	2	2015	WAU	Freetown	SL3.2.4	MH608100
NICD6605_SLE_WUR_2015-02-11	11	2	2015	WAU	Aberdeen	SL3.2.4	MH608101
NICD6640_SLE_WUR_2015-02-13	13	2	2015	WAU	Freetown	SL3.2.4	MH608102
NICD6643_SLE_WUR_2015-02-13	13	2	2015	WAU	Freetown	SL3.2.4	MH608103
NICD6644_SLE_WUR_2015-02-13	13	2	2015	WAU	Freetown	SL3.2.4	MH608104
NICD6672_SLE_WUR_2015-02-14	14	2	2015	WAU	Freetown	SL3.2.4	MH608105
NICD6823-swab_SLE_WUR_2015-02-26	26	2	2015	WAU	Freetown	SL3.2.5	MH608106
NICD7034_SLE_WUR_2015-03-06	6	3	2015	WAU	Aberdeen	SL3.2.4	MH608107
NICD7036_SLE_WUR_2015-03-07	7	3	2015	WAU	Aberdeen	SL3.2.4	MH608108

Table S2. Metadata for complete dataset used in this study

taxon	Date	Source	DISTRICT	lineage
EBOV_KT5317_KU296352_SLE_WesternRural_2015-02-24	15/02/24	Genbank	WAR	GIN
EBOV_PL4432_KU296481_SLE_Kambia_2015-03-07	15/03/07	Genbank	Kambia	GIN
EBOV_PL5249_KU296744_SLE_Kambia_2015-03-24	15/03/24	Genbank	Kambia	GIN
EBOV_PL5294_KU296764_SLE_Kambia_2015-03-24	15/03/24	Genbank	Kambia	GIN
EBOV_EM095_KM034550_SLE_Kailahun_2014-05-25	14/05/25	Genbank	Kailahun	SL1
EBOV_G3676_KM034554_SLE_Kailahun_2014-05-27	14/05/27	Genbank	Kailahun	SL1
EBOV_G3680_KM034559_SLE_Kailahun_2014-05-28	14/05/28	Genbank	Kailahun	SL1
EBOV_G3683_KM034561_SLE_Kailahun_2014-05-28	14/05/28	Genbank	Kailahun	SL1
EBOV_G3687_KM034563_SLE_Kailahun_2014-05-28	14/05/28	Genbank	Kailahun	SL1
EBOV_EM_079983_KR817227_SLE_Kailahun_2014-06-13	14/06/13	Genbank	Kailahun	SL1
EBOV_EM_080011_KR817229_SLE_Kailahun_2014-06-15	14/06/15	Genbank	Kailahun	SL1
EBOV_EM_080132_KR817236_SLE_Kailahun_2014-06-24	14/06/24	Genbank	Kailahun	SL1
EBOV_G3686_KM034562_SLE_Kailahun_2014-05-28	14/05/28	Genbank	Kailahun	SL2
EBOV_EM106_KM233036_SLE_Kailahun_2014-06-02	14/06/02	Genbank	Kailahun	SL2
EBOV_EM115_KM233041_SLE_Kailahun_2014-06-03	14/06/03	Genbank	Kailahun	SL2
EBOV_G3713_KM233050_SLE_Kailahun_2014-06-09	14/06/09	Genbank	Kailahun	SL2
EBOV_G3750_KM233058_SLE_Kailahun_2014-06-10	14/06/10	Genbank	Kailahun	SL2
EBOV_G3765_KM233064_SLE_Kailahun_2014-06-14	14/06/14	Genbank	Kailahun	SL2
EBOV_G3789_KM233076_SLE_Kailahun_2014-06-14	14/06/14	Genbank	Kailahun	SL2
EBOV_G3798_KM233079_SLE_Kailahun_2014-06-15	14/06/15	Genbank	Kailahun	SL2
EBOV_G3805_KM233082_SLE_Kailahun_2014-06-15	14/06/15	Genbank	Kailahun	SL2
EBOV_G3814_KM233089_SLE_Kailahun_2014-06-15	14/06/15	Genbank	Kailahun	SL2
EBOV_G3817_KM233091_SLE_Kailahun_2014-06-15	14/06/15	Genbank	Kailahun	SL2
EBOV_G3819_KM233093_SLE_Kailahun_2014-06-15	14/06/15	Genbank	Kailahun	SL2
EBOV_G3850_KM233111_SLE_Kailahun_2014-06-18	14/06/18	Genbank	Kailahun	SL2
EBOV_G3856_KM233113_SLE_Kenema_2014-06-18	14/06/18	Genbank	Kenema	SL2
EBOV_G3952_KR105211_SLE_Kailahun_2014-06-25	14/06/25	Genbank	Kailahun	SL2
EBOV_G4151_KR105215_SLE_Kenema_2014-07-06	14/07/06	Genbank	Kenema	SL2
EBOV_G4235_KR105220_SLE_Kailahun_2014-07-11	14/07/11	Genbank	Kailahun	SL2
EBOV_G4312_KR105230_SLE_PortLoko_2014-07-12	14/07/12	Genbank	Port Loko	SL2
EBOV_G4333_KR105236_SLE_Kailahun_2014-07-13	14/07/13	Genbank	Kailahun	SL2

EBOV_G4316_KR105231_SLE_Kenema_2014-07-13	14/07/13	Genbank	Kenema	SL2
EBOV_G4337_KR105238_SLE_Kailahun_2014-07-14	14/07/14	Genbank	Kailahun	SL2
EBOV_G4324_KR105233_SLE_Kenema_2014-07-14	14/07/14	Genbank	Kenema	SL2
EBOV_G4325_KR105234_SLE_Kenema_2014-07-14	14/07/14	Genbank	Kenema	SL2
EBOV_G4350_KR105242_SLE_Kenema_2014-07-14	14/07/14	Genbank	Kenema	SL2
EBOV_G4380_KR105243_SLE_Kenema_2014-07-15	14/07/15	Genbank	Kenema	SL2
EBOV_G4389_KR105245_SLE_Kenema_2014-07-16	14/07/16	Genbank	Kenema	SL2
EBOV_G4345_KR105239_SLE_Kenema_2014-07-18	14/07/18	Genbank	Kenema	SL2
EBOV_G4415_KR105247_SLE_Kenema_2014-07-18	14/07/18	Genbank	Kenema	SL2
EBOV_G4406_KR105246_SLE_Kono_2014-07-18	14/07/18	Genbank	Kono	SL2
EBOV_G4422_KR105250_SLE_Kenema_2014-07-19	14/07/19	Genbank	Kenema	SL2
EBOV_G4450_KR105257_SLE_Kenema_2014-07-21	14/07/21	Genbank	Kenema	SL2
EBOV_G4527_KR105261_SLE_Kenema_2014-07-26	14/07/26	Genbank	Kenema	SL2
EBOV_G4701_KR105264_SLE_Kenema_2014-08-03	14/08/03	Genbank	Kenema	SL2
EBOV_G4702_KR105265_SLE_Kenema_2014-08-03	14/08/03	Genbank	Kenema	SL2
EBOV_G4837_KR105272_SLE_Kenema_2014-08-09	14/08/09	Genbank	Kenema	SL2
EBOV_G4856_KR105273_SLE_Kenema_2014-08-10	14/08/10	Genbank	Kenema	SL2
EBOV_G4886_KR105276_SLE_Kenema_2014-08-11	14/08/11	Genbank	Kenema	SL2
EBOV_G4907_KR105277_SLE_Kenema_2014-08-12	14/08/12	Genbank	Kenema	SL2
EBOV_G4942_KR105279_SLE_Moyamba_2014-08-12	14/08/12	Genbank	Moyamba	SL2
EBOV_G4937_KR105278_SLE_Kenema_2014-08-13	14/08/13	Genbank	Kenema	SL2
EBOV_G4946_KR105280_SLE_Kenema_2014-08-13	14/08/13	Genbank	Kenema	SL2
EBOV_G4960_KR105283_SLE_Kenema_2014-08-14	14/08/14	Genbank	Kenema	SL2
EBOV_G4971_KR105284_SLE_Kenema_2014-08-14	14/08/14	Genbank	Kenema	SL2
EBOV_G5059_KR105296_SLE_Kenema_2014-08-18	14/08/18	Genbank	Kenema	SL2
EBOV_20140008_KR653251_SLE_Kenema_2014-08-22	14/08/22	Genbank	Kenema	SL2
EBOV_20140038_KR653267_SLE_Tonkolili_2014-08-23	14/08/23	Genbank	Tonkolili	SL2
EBOV_Makona-UK1_KP184503_SLE_Kenema_2014-08-25	14/08/25	Genbank	Kenema	SL2
EBOV_G5370_KR105306_SLE_Kenema_2014-08-28	14/08/28	Genbank	Kenema	SL2
EBOV_G5364_KR105305_SLE_UNKNOWN_2014-08-28	14/08/28	Genbank	UNKNOWN	SL2
EBOV_G5570_KR105310_SLE_Kenema_2014-09-08	14/09/08	Genbank	Kenema	SL2
EBOV_20140910_KR653269_SLE_Kenema_2014-09-18	14/09/18	Genbank	Kenema	SL2
EBOV_J0156_KP759698_SLE_WesternUrban_2014-11-07	14/11/07	Genbank	WAU	SL2
NICD4743_SLE_WR_2014-12-12	14/12/12	NICD	WAR	SL2

NICD5154_SLE_WUR_2014-12-23	14/12/23	NICD	WAU	SL2
EBOV_EM096_KM034551_SLE_Kailahun_2014-05-26	14/05/26	Genbank	Kailahun	SL3.1
EBOV_EM098_KM034552_SLE_Kailahun_2014-05-26	14/05/26	Genbank	Kailahun	SL3.1
EBOV_G3677_KM034556_SLE_Kailahun_2014-05-26	14/05/26	Genbank	Kailahun	SL3.1
EBOV_G3670_KM034553_SLE_Kailahun_2014-05-27	14/05/27	Genbank	Kailahun	SL3.1
EBOV_G3679_KM034558_SLE_Kailahun_2014-05-28	14/05/28	Genbank	Kailahun	SL3.1
EBOV_G3682_KM034560_SLE_Kailahun_2014-05-28	14/05/28	Genbank	Kailahun	SL3.1
EBOV_G3707_KM233049_SLE_Kailahun_2014-05-31	14/05/31	Genbank	Kailahun	SL3.1
EBOV_EM104_KM233035_SLE_Kailahun_2014-06-02	14/06/02	Genbank	Kailahun	SL3.1
EBOV_EM110_KM233037_SLE_Kailahun_2014-06-03	14/06/03	Genbank	Kailahun	SL3.1
EBOV_EM111_KM233038_SLE_Kailahun_2014-06-03	14/06/03	Genbank	Kailahun	SL3.1
EBOV_EM112_KM233039_SLE_Kailahun_2014-06-03	14/06/03	Genbank	Kailahun	SL3.1
EBOV_EM113_KM233040_SLE_Kailahun_2014-06-03	14/06/03	Genbank	Kailahun	SL3.1
EBOV_EM120_KM233043_SLE_Kailahun_2014-06-03	14/06/03	Genbank	Kailahun	SL3.1
EBOV_EM121_KM233044_SLE_Kailahun_2014-06-04	14/06/04	Genbank	Kailahun	SL3.1
EBOV_EM124_KM233045_SLE_Kailahun_2014-06-04	14/06/04	Genbank	Kailahun	SL3.1
EBOV_NM042_KM233116_SLE_Kambia_2014-06-04	14/06/04	Genbank	Kambia	SL3.1
EBOV_G3724_KM233053_SLE_Kailahun_2014-06-05	14/06/05	Genbank	Kailahun	SL3.1
EBOV_G3729_KM233054_SLE_Kailahun_2014-06-07	14/06/07	Genbank	Kailahun	SL3.1
EBOV_G3734_KM233055_SLE_Kailahun_2014-06-07	14/06/07	Genbank	Kailahun	SL3.1
EBOV_G3735_KM233056_SLE_Kailahun_2014-06-07	14/06/07	Genbank	Kailahun	SL3.1
EBOV_G3758_KM233062_SLE_Kailahun_2014-06-11	14/06/11	Genbank	Kailahun	SL3.1
EBOV_G3769_KM233065_SLE_Kailahun_2014-06-12	14/06/12	Genbank	Kailahun	SL3.1
EBOV_G3771_KM233071_SLE_Kailahun_2014-06-12	14/06/12	Genbank	Kailahun	SL3.1
EBOV_G3764_KM233063_SLE_UNKNOWN_2014-06-12	14/06/12	Genbank	UNKNOWN	SL3.1
EBOV_EM_080003_KR817228_SLE_Kailahun_2014-06-14	14/06/14	Genbank	Kailahun	SL3.1
EBOV_G3782_KM233072_SLE_Kailahun_2014-06-14	14/06/14	Genbank	Kailahun	SL3.1
EBOV_G3786_KM233073_SLE_Kailahun_2014-06-14	14/06/14	Genbank	Kailahun	SL3.1
EBOV_G3787_KM233074_SLE_Kailahun_2014-06-14	14/06/14	Genbank	Kailahun	SL3.1
EBOV_G3788_KM233075_SLE_Kailahun_2014-06-14	14/06/14	Genbank	Kailahun	SL3.1
EBOV_G3795_KM233077_SLE_Kailahun_2014-06-15	14/06/15	Genbank	Kailahun	SL3.1
EBOV_G3796_KM233078_SLE_Kailahun_2014-06-15	14/06/15	Genbank	Kailahun	SL3.1
EBOV_G3799_KM233080_SLE_Kailahun_2014-06-15	14/06/15	Genbank	Kailahun	SL3.1
EBOV_G3800_KM233081_SLE_Kailahun_2014-06-15	14/06/15	Genbank	Kailahun	SL3.1

EBOV_G3807_KM233084_SLE_Kailahun_2014-06-15	14/06/15	Genbank	Kailahun	SL3.1
EBOV_G3808_KM233085_SLE_Kailahun_2014-06-15	14/06/15	Genbank	Kailahun	SL3.1
EBOV_G3809_KM233086_SLE_Kailahun_2014-06-15	14/06/15	Genbank	Kailahun	SL3.1
EBOV_G3810_KM233087_SLE_Kailahun_2014-06-15	14/06/15	Genbank	Kailahun	SL3.1
EBOV_G3816_KM233090_SLE_Kailahun_2014-06-15	14/06/15	Genbank	Kailahun	SL3.1
EBOV_G3818_KM233092_SLE_Kailahun_2014-06-15	14/06/15	Genbank	Kailahun	SL3.1
EBOV_G3820_KM233094_SLE_Kailahun_2014-06-15	14/06/15	Genbank	Kailahun	SL3.1
EBOV_G3821_KM233095_SLE_Kailahun_2014-06-15	14/06/15	Genbank	Kailahun	SL3.1
EBOV_G3822_KM233096_SLE_Kailahun_2014-06-15	14/06/15	Genbank	Kailahun	SL3.1
EBOV_G3823_KM233097_SLE_Kailahun_2014-06-15	14/06/15	Genbank	Kailahun	SL3.1
EBOV_G3826_KM233100_SLE_Kailahun_2014-06-16	14/06/16	Genbank	Kailahun	SL3.1
EBOV_G3827_KM233101_SLE_Kailahun_2014-06-16	14/06/16	Genbank	Kailahun	SL3.1
EBOV_G3831_KM233103_SLE_Kailahun_2014-06-16	14/06/16	Genbank	Kailahun	SL3.1
EBOV_G3838_KR105200_SLE_Kailahun_2014-06-16	14/06/16	Genbank	Kailahun	SL3.1
EBOV_G3841_KM233107_SLE_Bo_2014-06-17	14/06/17	Genbank	Bo	SL3.1
EBOV_G3838_KM233105_SLE_Kailahun_2014-06-17	14/06/17	Genbank	Kailahun	SL3.1
EBOV_G3840_KM233106_SLE_Kailahun_2014-06-17	14/06/17	Genbank	Kailahun	SL3.1
EBOV_G3845_KM233108_SLE_Kailahun_2014-06-18	14/06/18	Genbank	Kailahun	SL3.1
EBOV_G3845_KR105201_SLE_Kenema_2014-06-19	14/06/19	Genbank	Kenema	SL3.1
EBOV_G3917_KR105207_SLE_Kenema_2014-06-21	14/06/21	Genbank	Kenema	SL3.1
EBOV_G3926_KR105208_SLE_Kailahun_2014-06-22	14/06/22	Genbank	Kailahun	SL3.1
EBOV_G3949_KR105209_SLE_UNKNOWN_2014-06-24	14/06/24	Genbank	UNKNOWN	SL3.1
EBOV_G3950_KR105210_SLE_UNKNOWN_2014-06-24	14/06/24	Genbank	UNKNOWN	SL3.1
EBOV_G3972_KR105212_SLE_UNKNOWN_2014-06-24	14/06/24	Genbank	UNKNOWN	SL3.1
EBOV_EM_080165_KR817238_SLE_Kailahun_2014-06-26	14/06/26	Genbank	Kailahun	SL3.1
EBOV_G4133_KR105214_SLE_Kailahun_2014-07-04	14/07/04	Genbank	Kailahun	SL3.1
EBOV_G4217_KR105218_SLE_Bo_2014-07-08	14/07/08	Genbank	Bo	SL3.1
EBOV_G4348_KR105241_SLE_Kenema_2014-07-09	14/07/09	Genbank	Kenema	SL3.1
EBOV_G4221_KR105219_SLE_UNKNOWN_2014-07-09	14/07/09	Genbank	UNKNOWN	SL3.1
EBOV_G4236_KR105221_SLE_Kailahun_2014-07-10	14/07/10	Genbank	Kailahun	SL3.1
EBOV_G4250_KR105222_SLE_Kailahun_2014-07-11	14/07/11	Genbank	Kailahun	SL3.1
EBOV_G4251_KR105223_SLE_Kailahun_2014-07-11	14/07/11	Genbank	Kailahun	SL3.1
EBOV_G4252_KR105224_SLE_Kailahun_2014-07-11	14/07/11	Genbank	Kailahun	SL3.1
EBOV_G4254_KR105225_SLE_Kailahun_2014-07-11	14/07/11	Genbank	Kailahun	SL3.1

EBOV_G4299_KR105229_SLE_Kailahun_2014-07-12	14/07/12	Genbank	Kailahun	SL3.1
EBOV_G4382_KR105244_SLE_Kenema_2014-07-16	14/07/16	Genbank	Kenema	SL3.1
EBOV_G4416_KR105248_SLE_Bo_2014-07-18	14/07/18	Genbank	Bo	SL3.1
EBOV_G4419_KR105249_SLE_Kenema_2014-07-19	14/07/19	Genbank	Kenema	SL3.1
EBOV_G4433_KR105254_SLE_Kailahun_2014-07-20	14/07/20	Genbank	Kailahun	SL3.1
EBOV_G4437_KR105255_SLE_Kenema_2014-07-20	14/07/20	Genbank	Kenema	SL3.1
EBOV_G4445_KR105256_SLE_Kenema_2014-07-21	14/07/21	Genbank	Kenema	SL3.1
EBOV_G4751_KR105271_SLE_UNKNOWN_2014-08-05	14/08/05	Genbank	UNKNOWN	SL3.1
EBOV_G4973_KR105286_SLE_UNKNOWN_2014-08-12	14/08/12	Genbank	UNKNOWN	SL3.1
EBOV_G5012_KR105292_SLE_WesternArea_2014-08-15	14/08/15	Genbank	Western Area	SL3.1
EBOV_G5016_KR105293_SLE_Kenema_2014-08-16	14/08/16	Genbank	Kenema	SL3.1
EBOV_20140100_KR653241_SLE_Pujehun_2014-08-24	14/08/24	Genbank	Pujehun	SL3.1
EBOV_20140161_KR653265_SLE_Kailahun_2014-08-27	14/08/27	Genbank	Kailahun	SL3.1
EBOV_20140254_KR653296_SLE_Bo_2014-08-29	14/08/29	Genbank	Bo	SL3.1
EBOV_20140517_KR653263_SLE_Kailahun_2014-09-05	14/09/05	Genbank	Kailahun	SL3.1
EBOV_G5571_KR105311_SLE_Kenema_2014-09-08	14/09/08	Genbank	Kenema	SL3.1
EBOV_20140729_KR653286_SLE_Bo_2014-09-10	14/09/10	Genbank	Bo	SL3.1
EBOV_G5640_KR105313_SLE_UNKNOWN_2014-09-11	14/09/11	Genbank	UNKNOWN	SL3.1
EBOV_G5644_KR105314_SLE_UNKNOWN_2014-09-11	14/09/11	Genbank	UNKNOWN	SL3.1
EBOV_G5647_KR105315_SLE_UNKNOWN_2014-09-11	14/09/11	Genbank	UNKNOWN	SL3.1
EBOV_20140933_KR653280_SLE_Moyamba_2014-09-16	14/09/16	Genbank	Moyamba	SL3.1
EBOV_G5731_KR105320_SLE_Moyamba_2014-09-16	14/09/16	Genbank	Moyamba	SL3.1
EBOV_G5737_KR105321_SLE_Moyamba_2014-09-16	14/09/16	Genbank	Moyamba	SL3.1
EBOV_G5763_KR105325_SLE_Moyamba_2014-09-16	14/09/16	Genbank	Moyamba	SL3.1
EBOV_G5765_KR105326_SLE_Moyamba_2014-09-16	14/09/16	Genbank	Moyamba	SL3.1
EBOV_G5767_KR105327_SLE_UNKNOWN_2014-09-16	14/09/16	Genbank	UNKNOWN	SL3.1
EBOV_G5756_KR105324_SLE_UNKNOWN_2014-09-18	14/09/18	Genbank	UNKNOWN	SL3.1
EBOV_20141043_KR653305_SLE_Moyamba_2014-09-21	14/09/21	Genbank	Moyamba	SL3.1
EBOV_20141061_KR653250_SLE_Moyamba_2014-09-21	14/09/21	Genbank	Moyamba	SL3.1
EBOV_G5844_KR105328_SLE_Moyamba_2014-09-21	14/09/21	Genbank	Moyamba	SL3.1
EBOV_20141012_KR653244_SLE_Pujehun_2014-09-21	14/09/21	Genbank	Pujehun	SL3.1
EBOV_G5879_KR105330_SLE_Pujehun_2014-09-22	14/09/22	Genbank	Pujehun	SL3.1
EBOV_20141241_KR653300_SLE_PortLoko_2014-09-24	14/09/24	Genbank	Port Loko	SL3.1

EBOV_G6060_KR105342_SLE_Pujehun_2014-09-25	14/09/25	Genbank	Pujehun	SL3.1
EBOV_G6062_KR105343_SLE_Pujehun_2014-09-25	14/09/25	Genbank	Pujehun	SL3.1
EBOV_20141227_KR653303_SLE_Bo_2014-09-26	14/09/26	Genbank	Bo	SL3.1
EBOV_20141643_KR653233_SLE_Pujehun_2014-10-04	14/10/04	Genbank	Pujehun	SL3.1
EBOV_20142065_KR653274_SLE_Kenema_2014-10-11	14/10/11	Genbank	Kenema	SL3.1
EBOV_20142477_KR653255_SLE_Moyamba_2014-10-19	14/10/19	Genbank	Moyamba	SL3.1
EBOV_20143164_KR653277_SLE_Kenema_2014-10-27	14/10/27	Genbank	Kenema	SL3.1
EBOV_20143466_KR653304_SLE_Kenema_2014-11-01	14/11/01	Genbank	Kenema	SL3.1
EBOV_EM119_KM233042_SLE_Kailahun_2014-06-03	14/06/03	Genbank	Kailahun	SL3.1.1
EBOV_G3770_KM233069_SLE_Kailahun_2014-06-12	14/06/12	Genbank	Kailahun	SL3.1.1
EBOV_G3846_KM233109_SLE_Kailahun_2014-06-18	14/06/18	Genbank	Kailahun	SL3.1.1
EBOV_G4190_KR105216_SLE_Kailahun_2014-07-07	14/07/07	Genbank	Kailahun	SL3.1.1
NICD0143_SLE_WUR_2014-08-31	14/08/31	NICD	WAU	SL3.1.1
EBOV_G6012_KR105340_SLE_UNKNOWN_2014-09-25	14/09/25	Genbank	UNKNOWN	SL3.1.1
NICD1275_SLE_WR_2014-09-25	14/09/25	NICD	WAR	SL3.1.1
EBOV_J0053_KP759721_SLE_WesternRural_2014-10-06	14/10/06	Genbank	WAR	SL3.1.1
EBOV_J0054_KP759722_SLE_WesternRural_2014-10-06	14/10/06	Genbank	WAR	SL3.1.1
EBOV_J0051_KP759633_SLE_WesternUrban_2014-10-07	14/10/07	Genbank	WAU	SL3.1.1
EBOV_20142407_KR653249_SLE_Tonkolili_2014-10-16	14/10/16	Genbank	Tonkolili	SL3.1.1
EBOV_20142417_KR653254_SLE_Bo_2014-10-19	14/10/19	Genbank	Bo	SL3.1.1
EBOV_J0092_KP759650_SLE_WesternRural_2014-10-25	14/10/25	Genbank	WAR	SL3.1.1
EBOV_J0099_KP759764_SLE_Kambia_2014-10-28	14/10/28	Genbank	Kambia	SL3.1.1
EBOV_J0137_KP759685_SLE_WesternRural_2014-11-01	14/11/01	Genbank	WAR	SL3.1.1
NICD2763-Vero_SLE_PL_2014-11-06	14/11/06	NICD	Port Loko	SL3.1.1
EBOV_20143964_KR653242_SLE_Bo_2014-11-07	14/11/07	Genbank	Bo	SL3.1.1
NICD3577_SLE_WR_2014-11-20	14/11/20	NICD	WAR	SL3.1.1
NICD3926_SLE_WUR_2014-11-27	14/11/27	NICD	WAU	SL3.1.1
NICD5584_SLE_WUR_2015-01-01	15/01/01	NICD	WAU	SL3.1.1
EBOV_77577_EMLK_KU296533_SLE_WesternUrban_2015-01-03	15/01/03	Genbank	WAU	SL3.1.1
EBOV_77578_EMLK_KU296806_SLE_WesternUrban_2015-01-03	15/01/03	Genbank	WAU	SL3.1.1
EBOV_77588_EMLK_KU296801_SLE_WesternUrban_2015-01-04	15/01/04	Genbank	WAU	SL3.1.1
EBOV_DML24708_KT357845_SLE_Kono_2015-01-28	15/01/28	Genbank	Kono	SL3.1.1
EBOV_KT5022_KU296519_SLE_WesternRural_2015-02-13	15/02/13	Genbank	WAR	SL3.1.1
EBOV_PL4226_KU296615_SLE_Kambia_2015-03-02	15/03/02	Genbank	Kambia	SL3.1.1

EBOV_PL4483_KU296389_SLE_Kambia_2015-03-08	15/03/08	Genbank	Kambia	SL3.1.1
EBOV_PL4736_KU296724_SLE_Kambia_2015-03-13	15/03/13	Genbank	Kambia	SL3.1.1
EBOV_PL4763_KU296503_SLE_Kambia_2015-03-14	15/03/14	Genbank	Kambia	SL3.1.1
EBOV_PL4864_KU296437_SLE_Kambia_2015-03-16	15/03/16	Genbank	Kambia	SL3.1.1
EBOV_PL4865_KU296633_SLE_Kambia_2015-03-16	15/03/16	Genbank	Kambia	SL3.1.1
EBOV_PL4866_KU296390_SLE_Kambia_2015-03-16	15/03/16	Genbank	Kambia	SL3.1.1
EBOV_PL5025_KU296412_SLE_Kambia_2015-03-20	15/03/20	Genbank	Kambia	SL3.1.1
EBOV_PL5157_KU296421_SLE_PortLoko_2015-03-22	15/03/22	Genbank	Port Loko	SL3.1.1
EBOV_PL6049_KU296743_SLE_Kambia_2015-04-11	15/04/11	Genbank	Kambia	SL3.1.1
EBOV_14743_EML_KU296298_SLE_Kambia_2015-04-19	15/04/19	Genbank	Kambia	SL3.1.1
EBOV_14846_EMLK_KU296831_SLE_Kambia_2015-04-29	15/04/29	Genbank	Kambia	SL3.1.1
EBOV_PL6656_KU296523_SLE_PortLoko_2015-05-01	15/05/01	Genbank	Port Loko	SL3.1.1
EBOV_15000_EMLK_KU296504_SLE_Kambia_2015-05-13	15/05/13	Genbank	Kambia	SL3.1.1
EBOV_PL7053_KU296757_SLE_PortLoko_2015-05-15	15/05/15	Genbank	Port Loko	SL3.1.1
EBOV_PL7055_KU296484_SLE_PortLoko_2015-05-15	15/05/15	Genbank	Port Loko	SL3.1.1
EBOV_PL7136b_KU296732_SLE_PortLoko_2015-05-18	15/05/18	Genbank	Port Loko	SL3.1.1
EBOV_PL7375_KU296498_SLE_PortLoko_2015-05-27	15/05/27	Genbank	Port Loko	SL3.1.1
EBOV_PL7376_KU296740_SLE_PortLoko_2015-05-27	15/05/27	Genbank	Port Loko	SL3.1.1
EBOV_PL7401_KU296819_SLE_PortLoko_2015-05-27	15/05/27	Genbank	Port Loko	SL3.1.1
EBOV_15207R_EMLK_KU296488_SLE_Kambia_2015-05-29	15/05/29	Genbank	Kambia	SL3.1.1
EBOV_PL7429_KU296617_SLE_PortLoko_2015-05-29	15/05/29	Genbank	Port Loko	SL3.1.1
EBOV_PL7451_KU296837_SLE_PortLoko_2015-05-29	15/05/29	Genbank	Port Loko	SL3.1.1
EBOV_PL7496_KU296846_SLE_PortLoko_2015-05-31	15/05/31	Genbank	Port Loko	SL3.1.1
EBOV_PL7511_KU296685_SLE_PortLoko_2015-06-01	15/06/01	Genbank	Port Loko	SL3.1.1
EBOV_PL7551_KU296322_SLE_PortLoko_2015-06-02	15/06/02	Genbank	Port Loko	SL3.1.1
EBOV_PL7577_KU296385_SLE_PortLoko_2015-06-03	15/06/03	Genbank	Port Loko	SL3.1.1
EBOV_15274_EMLK_KU296337_SLE_Kambia_2015-06-04	15/06/04	Genbank	Kambia	SL3.1.1
EBOV_PL7604_KU296592_SLE_PortLoko_2015-06-04	15/06/04	Genbank	Port Loko	SL3.1.1
EBOV_PL7605_KU296486_SLE_PortLoko_2015-06-04	15/06/04	Genbank	Port Loko	SL3.1.1
EBOV_PL7626_KU296753_SLE_PortLoko_2015-06-04	15/06/04	Genbank	Port Loko	SL3.1.1
EBOV_15314_EMLK_KU296600_SLE_Kambia_2015-06-06	15/06/06	Genbank	Kambia	SL3.1.1
EBOV_PL7678_KU296625_SLE_PortLoko_2015-06-06	15/06/06	Genbank	Port Loko	SL3.1.1
EBOV_PL7685_KU296556_SLE_PortLoko_2015-06-06	15/06/06	Genbank	Port Loko	SL3.1.1
EBOV_PL7689_KU296712_SLE_PortLoko_2015-06-06	15/06/06	Genbank	Port Loko	SL3.1.1

EBOV_PL7709_KU296426_SLE_PortLoko_2015-06-06	15/06/06	Genbank	Port Loko	SL3.1.1
EBOV_15323_EMLK_KU296668_SLE_Kambia_2015-06-07	15/06/07	Genbank	Kambia	SL3.1.1
EBOV_15327_EMLK_KU296649_SLE_Kambia_2015-06-07	15/06/07	Genbank	Kambia	SL3.1.1
EBOV_15338_EMLK_KU296414_SLE_Kambia_2015-06-08	15/06/08	Genbank	Kambia	SL3.1.1
EBOV_15360_EMLK_KU296399_SLE_Kambia_2015-06-08	15/06/08	Genbank	Kambia	SL3.1.1
EBOV_PL7773_KU296417_SLE_UNKNOWN_2015-06-08	15/06/08	Genbank	UNKNOWN	SL3.1.1
EBOV_PL7774_KU296589_SLE__2015-06-08	15/06/08	Genbank	UNKNOWN	SL3.1.1
EBOV_PL7775_KU296422_SLE_UNKNOWN_2015-06-08	15/06/08	Genbank	UNKNOWN	SL3.1.1
EBOV_15349_EMLK_KU296442_SLE_Kambia_2015-06-09	15/06/09	Genbank	Kambia	SL3.1.1
EBOV_PL7792_KU296793_SLE_PortLoko_2015-06-09	15/06/09	Genbank	Port Loko	SL3.1.1
EBOV_PL7801_KU296327_SLE_PortLoko_2015-06-09	15/06/09	Genbank	Port Loko	SL3.1.1
EBOV_PL7820_KU296804_SLE_PortLoko_2015-06-09	15/06/09	Genbank	Port Loko	SL3.1.1
EBOV_15373_EMLK_KU296802_SLE_Kambia_2015-06-10	15/06/10	Genbank	Kambia	SL3.1.1
EBOV_PL7834_KU296326_SLE_PortLoko_2015-06-10	15/06/10	Genbank	Port Loko	SL3.1.1
EBOV_PL7946_KU296667_SLE_PortLoko_2015-06-12	15/06/12	Genbank	Port Loko	SL3.1.1
EBOV_PL7947_KU296571_SLE_PortLoko_2015-06-12	15/06/12	Genbank	Port Loko	SL3.1.1
EBOV_15421_EMLK_KU296796_SLE_Kambia_2015-06-13	15/06/13	Genbank	Kambia	SL3.1.1
EBOV_15438R_EMLK_KU296353_SLE_Kambia_2015-06-14	15/06/14	Genbank	Kambia	SL3.1.1
EBOV_15470_EMLK_KU296543_SLE_Kambia_2015-06-16	15/06/16	Genbank	Kambia	SL3.1.1
EBOV_PL8128_KU296340_SLE_PortLoko_2015-06-16	15/06/16	Genbank	Port Loko	SL3.1.1
EBOV_PL8172_KU296428_SLE_PortLoko_2015-06-18	15/06/18	Genbank	Port Loko	SL3.1.1
EBOV_PL8310_KU296618_SLE_PortLoko_2015-06-21	15/06/21	Genbank	Port Loko	SL3.1.1
EBOV_15543_EMLK_KU296666_SLE_Kambia_2015-06-22	15/06/22	Genbank	Kambia	SL3.1.1
EBOV_PL8429_KU296440_SLE_PortLoko_2015-06-24	15/06/24	Genbank	Port Loko	SL3.1.1
EBOV_15661_EMLK_KU296822_SLE_Kambia_2015-06-28	15/06/28	Genbank	Kambia	SL3.1.1
EBOV_15674_EMLK_KU296843_SLE_Kambia_2015-06-28	15/06/28	Genbank	Kambia	SL3.1.1
EBOV_PL8630_KU296325_SLE_PortLoko_2015-06-29	15/06/29	Genbank	Port Loko	SL3.1.1
EBOV_15686_EMLK_KU296555_SLE_Kambia_2015-06-30	15/06/30	Genbank	Kambia	SL3.1.1
EBOV_PL8780_KU296404_SLE_PortLoko_2015-07-03	15/07/03	Genbank	Port Loko	SL3.1.1
EBOV_19521_EMLK_KU296306_SLE_Kambia_2015-07-05	15/07/05	Genbank	Kambia	SL3.1.1
EBOV_KT7095_KU296690_SLE_Kambia_2015-07-05	15/07/05	Genbank	Kambia	SL3.1.1
EBOV_PL8896_KU296342_SLE_PortLoko_2015-07-06	15/07/06	Genbank	Port Loko	SL3.1.1
EBOV_19560R_EMLK_KU296580_SLE_Kambia_2015-07-07	15/07/07	Genbank	Kambia	SL3.1.1
EBOV_PL9150_KU296522_SLE_PortLoko_2015-07-12	15/07/12	Genbank	Port Loko	SL3.1.1

EBOV_PL9192_KU296401_SLE_PortLoko_2015-07-13	15/07/13	Genbank	Port Loko	SL3.1.1
EBOV_PL9199_KU296371_SLE_PortLoko_2015-07-13	15/07/13	Genbank	Port Loko	SL3.1.1
EBOV_020380_EMLK_KU296775_SLE_Kambia_2015-08-28	15/08/28	Genbank	Kambia	SL3.1.1
EBOV_020484_EMLK_KU296462_SLE_Kambia_2015-09-04	15/09/04	Genbank	Kambia	SL3.1.1
EBOV_20524_EMLK_KU296424_SLE_Kambia_2015-09-06	15/09/06	Genbank	Kambia	SL3.1.1
EBOV_20525_EMLK_KU296487_SLE_Kambia_2015-09-06	15/09/06	Genbank	Kambia	SL3.1.1
EBOV_20547_EMLK_KU296455_SLE_Kambia_2015-09-07	15/09/07	Genbank	Kambia	SL3.1.1
EBOV_G5244_KR105301_SLE_UNKNOWN_2014-08-22	14/08/22	Genbank	UNKNOWN	SL3.1.2
NICD0022_SLE_WUR_2014-08-26	14/08/26	NICD	WAU	SL3.1.2
NICD0031_SLE_WUR_2014-08-26	14/08/26	NICD	WAU	SL3.1.2
NICD0034_SLE_WUR_2014-08-26	14/08/26	NICD	WAU	SL3.1.2
EBOV_20140174_KR653294_SLE_WesternUrban_2014-08-27	14/08/27	Genbank	WAU	SL3.1.2
NICD0393-Vero_SLE_WUR_2014-09-09	14/09/09	NICD	WAU	SL3.1.2
EBOV_G5723_KR105319_SLE_Bonthe_2014-09-15	14/09/15	Genbank	Bonthe	SL3.1.2
EBOV_20140872_KR653297_SLE_WesternUrban_2014-09-15	14/09/15	Genbank	WAU	SL3.1.2
NICD1113_SLE_BOM_2014-09-19	14/09/19	NICD	Bombali	SL3.1.2
NICD0821_SLE_WR_2014-09-20	14/09/20	NICD	WAR	SL3.1.2
NICD0983_SLE_WUR_2014-09-22	14/09/22	NICD	WAU	SL3.1.2
NICD0986-Vero_SLE_WUR_2014-09-22	14/09/22	NICD	WAU	SL3.1.2
NICD1209-Vero_SLE_WUR_2014-09-23	14/09/23	NICD	WAU	SL3.1.2
EBOV_G6069_KR105344_SLE_Kambia_2014-09-25	14/09/25	Genbank	Kambia	SL3.1.2
EBOV_G6020_KR105341_SLE_UNKNOWN_2014-09-25	14/09/25	Genbank	UNKNOWN	SL3.1.2
EBOV_20141352_KR653284_SLE_Kambia_2014-09-26	14/09/26	Genbank	Kambia	SL3.1.2
EBOV_J0005_KP759628_SLE_WesternRural_2014-09-26	14/09/26	Genbank	WAR	SL3.1.2
EBOV_J0001_KP759636_SLE_WesternRural_2014-09-27	14/09/27	Genbank	WAR	SL3.1.2
EBOV_J0007_KP759631_SLE_WesternRural_2014-09-27	14/09/27	Genbank	WAR	SL3.1.2
EBOV_J0002_KP759640_SLE_WesternUrban_2014-09-27	14/09/27	Genbank	WAU	SL3.1.2
EBOV_20141397_KR653288_SLE_Moyamba_2014-09-28	14/09/28	Genbank	Moyamba	SL3.1.2
EBOV_G6103_KR105348_SLE_Moyamba_2014-09-28	14/09/28	Genbank	Moyamba	SL3.1.2
EBOV_G6104_KR105349_SLE_Moyamba_2014-09-28	14/09/28	Genbank	Moyamba	SL3.1.2
EBOV_J0024_KP759663_SLE_WesternRural_2014-09-29	14/09/29	Genbank	WAR	SL3.1.2
EBOV_J0008_KP759718_SLE_WesternUrban_2014-09-29	14/09/29	Genbank	WAU	SL3.1.2
NICD1458_SLE_WUR_2014-09-29	14/09/29	NICD	WAU	SL3.1.2
EBOV_J0016_KP759742_SLE_WesternRural_2014-09-30	14/09/30	Genbank	WAR	SL3.1.2

EBOV_J0028_KP759683_SLE_Bombali_2014-10-02	14/10/02	Genbank	Bombali	SL3.1.2
EBOV_J0039_KP759620_SLE_PortLoko_2014-10-03	14/10/03	Genbank	Port Loko	SL3.1.2
EBOV_J0041_KP759710_SLE_WesternArea_2014-10-04	14/10/04	Genbank	Western Area	SL3.1.2
EBOV_J0042_KP759711_SLE_WesternRural_2014-10-04	14/10/04	Genbank	WAR	SL3.1.2
EBOV_J0050_KP759719_SLE_WesternUrban_2014-10-05	14/10/05	Genbank	WAU	SL3.1.2
EBOV_20141997_KR653293_SLE_Kono_2014-10-10	14/10/10	Genbank	Kono	SL3.1.2
EBOV_J0066_KP759730_SLE_WesternArea_2014-10-10	14/10/10	Genbank	Western Area	SL3.1.2
NICD2031-Vero_SLE_WUR_2014-10-11	14/10/11	NICD	WAU	SL3.1.2
NICD2032-Vero_SLE_WUR_2014-10-11	14/10/11	NICD	WAU	SL3.1.2
NICD2538_SLE_WUR_2014-10-22	14/10/22	NICD	WAU	SL3.1.2
NICD2541_SLE_WUR_2014-10-22	14/10/22	NICD	WAU	SL3.1.2
EBOV_J0089_KP759758_SLE_WesternArea_2014-10-23	14/10/23	Genbank	Western Area	SL3.1.2
EBOV_20143317_KR653282_SLE_Bombali_2014-10-29	14/10/29	Genbank	Bombali	SL3.1.2
EBOV_J0121_KP759669_SLE_WesternUrban_2014-10-29	14/10/29	Genbank	WAU	SL3.1.2
EBOV_J0113_KP759662_SLE_WesternUrban_2014-10-30	14/10/30	Genbank	WAU	SL3.1.2
EBOV_J0112_KP759661_SLE_WesternArea_2014-10-31	14/10/31	Genbank	Western Area	SL3.1.2
EBOV_J0134_KP759682_SLE_WesternRural_2014-10-31	14/10/31	Genbank	WAR	SL3.1.2
EBOV_20143458_KR653245_SLE_Tonkolili_2014-11-01	14/11/01	Genbank	Tonkolili	SL3.1.2
EBOV_J0135_KP759602_SLE_WesternRural_2014-11-03	14/11/03	Genbank	WAR	SL3.1.2
EBOV_J0143_KP759604_SLE_Kambia_2014-11-04	14/11/04	Genbank	Kambia	SL3.1.2
EBOV_J0144_KP759605_SLE_Kambia_2014-11-04	14/11/04	Genbank	Kambia	SL3.1.2
EBOV_J0159_KP759619_SLE_WesternRural_2014-11-05	14/11/05	Genbank	WAR	SL3.1.2
NICD2748_SLE_KMB_2014-11-06	14/11/06	NICD	Kambia	SL3.1.2
EBOV_J0150_KP759696_SLE_WesternUrban_2014-11-06	14/11/06	Genbank	WAU	SL3.1.2
EBOV_J0157_KP759699_SLE_WesternUrban_2014-11-06	14/11/06	Genbank	WAU	SL3.1.2
EBOV_J0154_KP759617_SLE_WesternArea_2014-11-07	14/11/07	Genbank	Western Area	SL3.1.2
EBOV_J0146_KP759610_SLE_WesternUrban_2014-11-07	14/11/07	Genbank	WAU	SL3.1.2
EBOV_J0147_KP759611_SLE_WesternUrban_2014-11-07	14/11/07	Genbank	WAU	SL3.1.2
EBOV_J0149_KP759695_SLE_WesternUrban_2014-11-07	14/11/07	Genbank	WAU	SL3.1.2
EBOV_J0171_KP759707_SLE_WesternRural_2014-11-10	14/11/10	Genbank	WAR	SL3.1.2

NICD3031_SLE_PL_2014-11-11	14/11/11	NICD	Port Loko	SL3.1.2
EBOV_J0174_KP759708_SLE_WesternArea_2014-11-11	14/11/11	Genbank	Western Area	SL3.1.2
EBOV_J0173_KP759627_SLE_WesternRural_2014-11-11	14/11/11	Genbank	WAR	SL3.1.2
EBOV_20144521_KR653271_SLE_Kono_2014-11-12	14/11/12	Genbank	Kono	SL3.1.2
NICD3089_SLE_PL_2014-11-12	14/11/12	NICD	Port Loko	SL3.1.2
NICD3087_SLE_WUR_2014-11-13	14/11/13	NICD	WAU	SL3.1.2
NICD3335_SLE_WR_2014-11-16	14/11/16	NICD	WAR	SL3.1.2
NICD3483-Vero_SLE_WUR_2014-11-18	14/11/18	NICD	WAU	SL3.1.2
NICD3596_SLE_WUR_2014-11-21	14/11/21	NICD	WAU	SL3.1.2
EBOV_20146001_KR653237_SLE_Kono_2014-11-24	14/11/24	Genbank	Kono	SL3.1.2
NICD3707_SLE_WUR_2014-11-24	14/11/24	NICD	WAU	SL3.1.2
NICD3823_SLE_WR_2014-11-25	14/11/25	NICD	WAR	SL3.1.2
NICD3820_SLE_WUR_2014-11-25	14/11/25	NICD	WAU	SL3.1.2
NICD3928_SLE_WUR_2014-11-26	14/11/26	NICD	WAU	SL3.1.2
NICD3932_SLE_WUR_2014-11-26	14/11/26	NICD	WAU	SL3.1.2
NICD3937_SLE_WUR_2014-11-26	14/11/26	NICD	WAU	SL3.1.2
NICD3925_SLE_WUR_2014-11-27	14/11/27	NICD	WAU	SL3.1.2
NICD4055_SLE_WUR_2014-11-28	14/11/28	NICD	WAU	SL3.1.2
NICD4025-Vero_SLE_WUR_2014-11-29	14/11/29	NICD	WAU	SL3.1.2
NICD4064-swab_SLE_WUR_2014-11-29	14/11/29	NICD	WAU	SL3.1.2
NICD4279-Vero_SLE_WUR_2014-12-05	14/12/05	NICD	WAU	SL3.1.2
EBOV_KT2449_KU296465_SLE_WesternUrban_2014-12-05	14/12/05	Genbank	WAU	SL3.1.2
NICD4682_SLE_WUR_2014-12-13	14/12/13	NICD	WAU	SL3.1.2
NICD4830_SLE_WUR_2014-12-15	14/12/15	NICD	WAU	SL3.1.2
EBOV_MK0384_KU296628_SLE_Bombali_2014-12-22	14/12/22	Genbank	Bombali	SL3.1.2
EBOV_MK0464_KU296835_SLE_UNKNOWN_2014-12-24	14/12/24	Genbank	UNKNOWN	SL3.1.2
NICD5200_SLE_WUR_2014-12-24	14/12/24	NICD	WAU	SL3.1.2
NICD5260_SLE_WUR_2014-12-24	14/12/24	NICD	WAU	SL3.1.2
NICD5266_SLE_WUR_2014-12-24	14/12/24	NICD	WAU	SL3.1.2
NICD5314_SLE_WUR_2014-12-26	14/12/26	NICD	WAU	SL3.1.2
NICD5354_SLE_WUR_2014-12-27	14/12/27	NICD	WAU	SL3.1.2
NICD5360_SLE_WUR_2014-12-27	14/12/27	NICD	WAU	SL3.1.2
EBOV_77524_EMLK_KU296368_SLE__2014-12-30	14/12/30	Genbank	UNKNOWN	SL3.1.2

EBOV_77529_EMLK_KU296477_SLE__2014-12-30	14/12/30	Genbank	UNKNOWN	SL3.1.2
EBOV_77551_EMLK_KU296808_SLE__2015-01-01	15/01/01	Genbank	UNKNOWN	SL3.1.2
EBOV_77572_EMLK_KU296508_SLE__2015-01-03	15/01/03	Genbank	UNKNOWN	SL3.1.2
EBOV_PL1387_KU296434_SLE_PortLoko_2015-01-04	15/01/04	Genbank	Port Loko	SL3.1.2
EBOV_MK0865_KU296500_SLE_UNKNOWN_2015-01-05	15/01/05	Genbank	UNKNOWN	SL3.1.2
EBOV_MK0866_KU296497_SLE_UNKNOWN_2015-01-05	15/01/05	Genbank	UNKNOWN	SL3.1.2
EBOV_MK0867_KU296748_SLE_UNKNOWN_2015-01-05	15/01/05	Genbank	UNKNOWN	SL3.1.2
EBOV_77601_EMLK_KU296826_SLE_WesternUrban_2015-01-05	15/01/05	Genbank	WAU	SL3.1.2
EBOV_MK0885_KU296758_SLE_Bombali_2015-01-06	15/01/06	Genbank	Bombali	SL3.1.2
EBOV_MK0888_KU296376_SLE_UNKNOWN_2015-01-06	15/01/06	Genbank	UNKNOWN	SL3.1.2
EBOV_MK0889_KU296794_SLE_UNKNOWN_2015-01-06	15/01/06	Genbank	UNKNOWN	SL3.1.2
NICD5725_SLE_WUR_2015-01-07	15/01/07	NICD	WAU	SL3.1.2
EBOV_MK1040_KU296528_SLE_Bombali_2015-01-11	15/01/11	Genbank	Bombali	SL3.1.2
EBOV_MK1079_KU296815_SLE_Bombali_2015-01-11	15/01/11	Genbank	Bombali	SL3.1.2
EBOV_DML24502_KT357825_SLE_Kono_2015-01-13	15/01/13	Genbank	Kono	SL3.1.2
EBOV_DML24504_KT357826_SLE_Kono_2015-01-13	15/01/13	Genbank	Kono	SL3.1.2
EBOV_75674_EMLH_KU296350_SLE_WesternUrban_2015-01-13	15/01/13	Genbank	WAU	SL3.1.2
EBOV_DML24506_KT357827_SLE_Kono_2015-01-14	15/01/14	Genbank	Kono	SL3.1.2
EBOV_DML24511_KT357828_SLE_Kono_2015-01-14	15/01/14	Genbank	Kono	SL3.1.2
EBOV_75731_EMLH_KU296564_SLE_WesternUrban_2015-01-16	15/01/16	Genbank	WAU	SL3.1.2
EBOV_DML24552_KT357829_SLE_Kono_2015-01-17	15/01/17	Genbank	Kono	SL3.1.2
EBOV_DML24553_KT357830_SLE_Kono_2015-01-17	15/01/17	Genbank	Kono	SL3.1.2
EBOV_DML24573_KT357831_SLE_Kono_2015-01-18	15/01/18	Genbank	Kono	SL3.1.2
EBOV_DML24581_KT357832_SLE_Kono_2015-01-19	15/01/19	Genbank	Kono	SL3.1.2
EBOV_DML24592_KT357833_SLE_Kono_2015-01-20	15/01/20	Genbank	Kono	SL3.1.2
EBOV_DML24601_KT357834_SLE_Kono_2015-01-20	15/01/20	Genbank	Kono	SL3.1.2
EBOV_DML24604_KT357835_SLE_Kono_2015-01-20	15/01/20	Genbank	Kono	SL3.1.2
EBOV_DML24605_KT357836_SLE_Kono_2015-01-20	15/01/20	Genbank	Kono	SL3.1.2
EBOV_DML24606_KT357837_SLE_Kono_2015-01-20	15/01/20	Genbank	Kono	SL3.1.2
EBOV_DML24608_KT357838_SLE_Kono_2015-01-20	15/01/20	Genbank	Kono	SL3.1.2
NICD6123_SLE_WR_2015-01-20	15/01/20	NICD	WAR	SL3.1.2
EBOV_DML24611_KT357839_SLE_Kono_2015-01-21	15/01/21	Genbank	Kono	SL3.1.2
EBOV_DML24620_KT357840_SLE_Kono_2015-01-21	15/01/21	Genbank	Kono	SL3.1.2
EBOV_361_EMLH_KU296420_SLE_WesternUrban_2015-01-21	15/01/21	Genbank	WAU	SL3.1.2

NICD6141_SLE_WR_2015-01-21	15/01/21	NICD	WAR	SL3.1.2
NICD6218_SLE_WUR_2015-01-24	15/01/24	NICD	WAU	SL3.1.2
EBOV_DML24669_KT357841_SLE_Kono_2015-01-25	15/01/25	Genbank	Kono	SL3.1.2
EBOV_DML24677_KT357842_SLE_Kono_2015-01-25	15/01/25	Genbank	Kono	SL3.1.2
EBOV_DML24683_KT357843_SLE_Kono_2015-01-26	15/01/26	Genbank	Kono	SL3.1.2
NICD6274_SLE_WUR_2015-01-26	15/01/26	NICD	WAU	SL3.1.2
EBOV_DML24706_KT357844_SLE_Kono_2015-01-28	15/01/28	Genbank	Kono	SL3.1.2
EBOV_DML24720_KT357846_SLE_Kono_2015-01-29	15/01/29	Genbank	Kono	SL3.1.2
EBOV_473_EMLH_KU296524_SLE_UNKNOWN_2015-01-29	15/01/29	Genbank	UNKNOWN	SL3.1.2
EBOV_KT4553_KU296698_SLE_UNKNOWN_2015-01-29	15/01/29	Genbank	UNKNOWN	SL3.1.2
EBOV_KT4555_KU296687_SLE_UNKNOWN_2015-01-29	15/01/29	Genbank	UNKNOWN	SL3.1.2
NICD6365_SLE_WR_2015-01-29	15/01/29	NICD	WAR	SL3.1.2
EBOV_DML24758_KT357847_SLE_Kono_2015-01-30	15/01/30	Genbank	Kono	SL3.1.2
EBOV_490_EMLH_KU296746_SLE_UNKNOWN_2015-01-30	15/01/30	Genbank	UNKNOWN	SL3.1.2
EBOV_491_EMLH_KU296588_SLE_UNKNOWN_2015-01-30	15/01/30	Genbank	UNKNOWN	SL3.1.2
EBOV_508_EMLH_KU296304_SLE_WesternUrban_2015-01-31	15/01/31	Genbank	WAU	SL3.1.2
EBOV_539_EMLH_KU296823_SLE_WesternUrban_2015-02-02	15/02/02	Genbank	WAU	SL3.1.2
EBOV_DML24818_KT357848_SLE_Kono_2015-02-03	15/02/03	Genbank	Kono	SL3.1.2
EBOV_567_EMLH_KU296427_SLE_UNKNOWN_2015-02-03	15/02/03	Genbank	UNKNOWN	SL3.1.2
EBOV_KT4663_KU296570_SLE_UNKNOWN_2015-02-03	15/02/03	Genbank	UNKNOWN	SL3.1.2
EBOV_DML24825_KT357849_SLE_Tonkolili_2015-02-04	15/02/04	Genbank	Tonkolili	SL3.1.2
EBOV_592_EMLH_KU296447_SLE_UNKNOWN_2015-02-05	15/02/05	Genbank	UNKNOWN	SL3.1.2
EBOV_DML24853_KT357850_SLE_Kono_2015-02-06	15/02/06	Genbank	Kono	SL3.1.2
EBOV_DML24854_KT357851_SLE_Kono_2015-02-06	15/02/06	Genbank	Kono	SL3.1.2
EBOV_704_EMLH_KU296721_SLE_UNKNOWN_2015-02-09	15/02/09	Genbank	UNKNOWN	SL3.1.2
EBOV_DML25123_KT357854_SLE_Kenema_2015-02-18	15/02/18	Genbank	Kenema	SL3.1.2
EBOV_DML25083_KT357852_SLE_Kono_2015-02-18	15/02/18	Genbank	Kono	SL3.1.2
EBOV_DML25103_KT357853_SLE_Kono_2015-02-19	15/02/19	Genbank	Kono	SL3.1.2
EBOV_PL4119_KU296645_SLE_PortLoko_2015-02-28	15/02/28	Genbank	Port Loko	SL3.1.2
EBOV_PL4197_KU296299_SLE_PortLoko_2015-03-02	15/03/02	Genbank	Port Loko	SL3.1.2
EBOV_PL4203_KU296311_SLE_PortLoko_2015-03-02	15/03/02	Genbank	Port Loko	SL3.1.2
EBOV_PL4248_KU296658_SLE_PortLoko_2015-03-03	15/03/03	Genbank	Port Loko	SL3.1.2
EBOV_PL4249_KU296349_SLE_PortLoko_2015-03-03	15/03/03	Genbank	Port Loko	SL3.1.2
EBOV_PL4283_KU296840_SLE_PortLoko_2015-03-03	15/03/03	Genbank	Port Loko	SL3.1.2

EBOV_PL4284_KU296829_SLE_PortLoko_2015-03-03	15/03/03	Genbank	Port Loko	SL3.1.2
EBOV_PL4443_KU296418_SLE_PortLoko_2015-03-07	15/03/07	Genbank	Port Loko	SL3.1.2
EBOV_PL4785_KU296688_SLE_PortLoko_2015-03-14	15/03/14	Genbank	Port Loko	SL3.1.2
EBOV_PL4813_KU296798_SLE_PortLoko_2015-03-14	15/03/14	Genbank	Port Loko	SL3.1.2
EBOV_PL4804_KU296766_SLE_PortLoko_2015-03-15	15/03/15	Genbank	Port Loko	SL3.1.2
EBOV_PL4844_KU296527_SLE_PortLoko_2015-03-16	15/03/16	Genbank	Port Loko	SL3.1.2
EBOV_PL4886_KU296461_SLE_PortLoko_2015-03-16	15/03/16	Genbank	Port Loko	SL3.1.2
EBOV_PL4932_KU296547_SLE_PortLoko_2015-03-18	15/03/18	Genbank	Port Loko	SL3.1.2
EBOV_PL5133_KU296489_SLE_PortLoko_2015-03-21	15/03/21	Genbank	Port Loko	SL3.1.2
EBOV_PL5275_KU296439_SLE_PortLoko_2015-03-24	15/03/24	Genbank	Port Loko	SL3.1.2
EBOV_PL5534_KU296784_SLE_PortLoko_2015-03-29	15/03/29	Genbank	Port Loko	SL3.1.2
EBOV_PL5674_KU296699_SLE_PortLoko_2015-03-31	15/03/31	Genbank	Port Loko	SL3.1.2
EBOV_PL5950_KU296559_SLE_PortLoko_2015-04-08	15/04/08	Genbank	Port Loko	SL3.1.2
EBOV_G3752_KM233061_SLE_Kailahun_2014-06-10	14/06/10	Genbank	Kailahun	SL3.2
EBOV_G3825_KM233098_SLE_Kailahun_2014-06-16	14/06/16	Genbank	Kailahun	SL3.2
EBOV_G3829_KM233102_SLE_Kailahun_2014-06-16	14/06/16	Genbank	Kailahun	SL3.2
EBOV_G3834_KM233104_SLE_Kailahun_2014-06-17	14/06/17	Genbank	Kailahun	SL3.2
EBOV_G3855_KR105203_SLE_Kenema_2014-06-17	14/06/17	Genbank	Kenema	SL3.2
EBOV_G3848_KM233110_SLE_Kailahun_2014-06-18	14/06/18	Genbank	Kailahun	SL3.2
EBOV_G3857_KM233115_SLE_Kailahun_2014-06-18	14/06/18	Genbank	Kailahun	SL3.2
EBOV_G3851_KM233112_SLE_Kenema_2014-06-18	14/06/18	Genbank	Kenema	SL3.2
EBOV_G3851_KR105202_SLE_Kenema_2014-06-18	14/06/18	Genbank	Kenema	SL3.2
EBOV_G3886_KR105204_SLE_Kenema_2014-06-19	14/06/19	Genbank	Kenema	SL3.2
EBOV_G3889_KR105205_SLE_Kenema_2014-06-19	14/06/19	Genbank	Kenema	SL3.2
EBOV_G3913_KR105206_SLE_Kenema_2014-06-21	14/06/21	Genbank	Kenema	SL3.2
EBOV_G4132_KR105213_SLE_Kenema_2014-07-05	14/07/05	Genbank	Kenema	SL3.2
EBOV_G4200_KR105217_SLE_Kenema_2014-07-08	14/07/08	Genbank	Kenema	SL3.2
EBOV_G4255_KR105226_SLE_Kailahun_2014-07-11	14/07/11	Genbank	Kailahun	SL3.2
EBOV_G4263_KR105227_SLE_Kenema_2014-07-11	14/07/11	Genbank	Kenema	SL3.2
EBOV_G4264_KR105228_SLE_Kenema_2014-07-11	14/07/11	Genbank	Kenema	SL3.2
EBOV_EM_080265_KR817244_SLE_Kailahun_2014-07-12	14/07/12	Genbank	Kailahun	SL3.2
EBOV_G4329_KR105235_SLE_Kailahun_2014-07-14	14/07/14	Genbank	Kailahun	SL3.2
EBOV_G4323_KR105232_SLE_Kenema_2014-07-14	14/07/14	Genbank	Kenema	SL3.2
EBOV_G4334_KR105237_SLE_Kenema_2014-07-14	14/07/14	Genbank	Kenema	SL3.2

EBOV_G4347_KR105240_SLE_Kenema_2014-07-15	14/07/15	Genbank	Kenema	SL3.2
EBOV_G4423_KR105251_SLE_Kailahun_2014-07-19	14/07/19	Genbank	Kailahun	SL3.2
EBOV_G4424_KR105252_SLE_Kailahun_2014-07-19	14/07/19	Genbank	Kailahun	SL3.2
EBOV_G4431_KR105253_SLE_Bo_2014-07-20	14/07/20	Genbank	Bo	SL3.2
EBOV_G4454_KR105258_SLE_Kailahun_2014-07-21	14/07/21	Genbank	Kailahun	SL3.2
EBOV_G4466_KR105260_SLE_Bo_2014-07-22	14/07/22	Genbank	Bo	SL3.2
EBOV_G4683_KR105262_SLE_UNKNOWN_2014-08-01	14/08/01	Genbank	UNKNOWN	SL3.2
EBOV_G4698_KR105263_SLE_Kenema_2014-08-03	14/08/03	Genbank	Kenema	SL3.2
EBOV_G4717_KR105266_SLE_Kenema_2014-08-04	14/08/04	Genbank	Kenema	SL3.2
EBOV_G4465_KR105259_SLE_Kenema_2014-08-05	14/08/05	Genbank	Kenema	SL3.2
EBOV_G4730_KR105268_SLE_Kenema_2014-08-05	14/08/05	Genbank	Kenema	SL3.2
EBOV_G4736_KR105269_SLE_Kenema_2014-08-05	14/08/05	Genbank	Kenema	SL3.2
EBOV_G4748_KR105270_SLE_UNKNOWN_2014-08-05	14/08/05	Genbank	UNKNOWN	SL3.2
EBOV_G4981_KR105287_SLE_UNKNOWN_2014-08-14	14/08/14	Genbank	UNKNOWN	SL3.2
EBOV_G4982_KR105288_SLE_UNKNOWN_2014-08-14	14/08/14	Genbank	UNKNOWN	SL3.2
EBOV_G4996_KR105290_SLE_UNKNOWN_2014-08-15	14/08/15	Genbank	UNKNOWN	SL3.2
EBOV_G5039_KR105295_SLE_Kenema_2014-08-17	14/08/17	Genbank	Kenema	SL3.2
NICD0023_SLE_TONK_2014-08-26	14/08/26	NICD	Tonkolili	SL3.2
EBOV_20140489_KR653235_SLE_Kenema_2014-09-04	14/09/04	Genbank	Kenema	SL3.2
EBOV_20140590_KR653278_SLE_Kenema_2014-09-07	14/09/07	Genbank	Kenema	SL3.2
NICD0520_SLE_BOM_2014-09-11	14/09/11	NICD	Bombali	SL3.2
EBOV_Makona-201403147_KP240931_SLE_UNKNOWN_2014-09-13	14/09/13	Genbank	UNKNOWN	SL3.2
EBOV_J0162_KP759622_SLE_Kambia_2014-11-09	14/11/09	Genbank	Kambia	SL3.2
EBOV_KT2669_KU296726_SLE_UNKNOWN_2014-12-13	14/12/13	Genbank	UNKNOWN	SL3.2
NICD5279-swab_SLE_WUR_2014-12-24	14/12/24	NICD	WAU	SL3.2
EBOV_77591_EMLK_KU296836_SLE_WesternUrban_2015-01-04	15/01/04	Genbank	WAU	SL3.2
EBOV_KT4715_KU296707_SLE_UNKNOWN_2015-02-06	15/02/06	Genbank	UNKNOWN	SL3.2
EBOV_PL4094_KU296476_SLE_Kambia_2015-02-27	15/02/27	Genbank	Kambia	SL3.2
EBOV_PL4347_KU296720_SLE_Kambia_2015-03-04	15/03/04	Genbank	Kambia	SL3.2
EBOV_PL4872_KU296419_SLE_Kambia_2015-03-16	15/03/16	Genbank	Kambia	SL3.2
EBOV_PL5260_KU296591_SLE_Kambia_2015-03-23	15/03/23	Genbank	Kambia	SL3.2
EBOV_PL5361_KU296415_SLE_Kambia_2015-03-26	15/03/26	Genbank	Kambia	SL3.2
EBOV_PL5428_KU296532_SLE_Kambia_2015-03-27	15/03/27	Genbank	Kambia	SL3.2

EBOV_PL5801_KU296844_SLE_Kambia_2015-04-04	15/04/04	Genbank	Kambia	SL3.2
EBOV_PL5803_KU296795_SLE_Kambia_2015-04-04	15/04/04	Genbank	Kambia	SL3.2
EBOV_PL6070_KU296614_SLE_Kambia_2015-04-11	15/04/11	Genbank	Kambia	SL3.2
EBOV_PL6158_KU296308_SLE_Kambia_2015-04-13	15/04/13	Genbank	Kambia	SL3.2
EBOV_14723_EML_KU296541_SLE_Kambia_2015-04-18	15/04/18	Genbank	Kambia	SL3.2
EBOV_14729_EML_KU296392_SLE_Kambia_2015-04-18	15/04/18	Genbank	Kambia	SL3.2
EBOV_14748_EML_KU296769_SLE_Kambia_2015-04-19	15/04/19	Genbank	Kambia	SL3.2
EBOV_14750_EML_KU296459_SLE_Kambia_2015-04-20	15/04/20	Genbank	Kambia	SL3.2
EBOV_14795_EMLK_KU296737_SLE_Kambia_2015-04-25	15/04/25	Genbank	Kambia	SL3.2
EBOV_14859_EMLK_KU296438_SLE_Kambia_2015-04-30	15/04/30	Genbank	Kambia	SL3.2
EBOV_G5295_KR105302_SLE_UNKNOWN_2014-08-25	14/08/25	Genbank	UNKNOWN	SL3.2.1
EBOV_G5296_KR105303_SLE_UNKNOWN_2014-08-25	14/08/25	Genbank	UNKNOWN	SL3.2.1
EBOV_20140134_KR653227_SLE_Bombali_2014-08-26	14/08/26	Genbank	Bombali	SL3.2.1
NICD0787_SLE_BOM_2014-09-17	14/09/17	NICD	Bombali	SL3.2.1
NICD0689_SLE_TONK_2014-09-17	14/09/17	NICD	Tonkolili	SL3.2.1
NICD1109_SLE_BOM_2014-09-19	14/09/19	NICD	Bombali	SL3.2.1
NICD1081_SLE_BOM_2014-09-20	14/09/20	NICD	Bombali	SL3.2.1
NICD1092_SLE_BOM_2014-09-20	14/09/20	NICD	Bombali	SL3.2.1
NICD0905_SLE_TONK_2014-09-20	14/09/20	NICD	Tonkolili	SL3.2.1
NICD0908_SLE_TONK_2014-09-20	14/09/20	NICD	Tonkolili	SL3.2.1
NICD0911_SLE_TONK_2014-09-20	14/09/20	NICD	Tonkolili	SL3.2.1
NICD1091_SLE_BOM_2014-09-20	14/09/20	NICD	Bombali	SL3.2.1
NICD1105_SLE_BOM_2014-09-21	14/09/21	NICD	Bombali	SL3.2.1
NICD0964_SLE_TONK_2014-09-21	14/09/21	NICD	Tonkolili	SL3.2.1
NICD1227_SLE_BOM_2014-09-22	14/09/22	NICD	Bombali	SL3.2.1
NICD1256-Vero_SLE_BOM_2014-09-22	14/09/22	NICD	Bombali	SL3.2.1
NICD1261_SLE_BOM_2014-09-22	14/09/22	NICD	Bombali	SL3.2.1
NICD1272-Vero_SLE_BOM_2014-09-22	14/09/22	NICD	Bombali	SL3.2.1
NICD1142_SLE_PL_2014-09-22	14/09/22	NICD	Port Loko	SL3.2.1
NICD1143_SLE_PL_2014-09-23	14/09/23	NICD	Port Loko	SL3.2.1
NICD1136_SLE_WR_2014-09-23	14/09/23	NICD	WAR	SL3.2.1
NICD1329-Vero_SLE_BOM_2014-09-24	14/09/24	NICD	Bombali	SL3.2.1
EBOV_J0019_KP759754_SLE_Bombali_2014-09-25	14/09/25	Genbank	Bombali	SL3.2.1
EBOV_J0020_KP759755_SLE_Bombali_2014-09-25	14/09/25	Genbank	Bombali	SL3.2.1

EBOV_J0022_KP759757_SLE_Bombali_2014-09-25	14/09/25	Genbank	Bombali	SL3.2.1
EBOV_20141232_KR653266_SLE_Tonkolili_2014-09-25	14/09/25	Genbank	Tonkolili	SL3.2.1
EBOV_J0015_KP759741_SLE_Bombali_2014-09-26	14/09/26	Genbank	Bombali	SL3.2.1
EBOV_J0017_KP759747_SLE_Bombali_2014-09-26	14/09/26	Genbank	Bombali	SL3.2.1
EBOV_J0021_KP759756_SLE_Bombali_2014-09-29	14/09/29	Genbank	Bombali	SL3.2.1
EBOV_20141497_KR653273_SLE_Bombali_2014-10-01	14/10/01	Genbank	Bombali	SL3.2.1
EBOV_20141960_KR653283_SLE_Tonkolili_2014-10-09	14/10/09	Genbank	Tonkolili	SL3.2.1
EBOV_20142127_KR653234_SLE_Bombali_2014-10-12	14/10/12	Genbank	Bombali	SL3.2.1
EBOV_20142260_KR653262_SLE_Bombali_2014-10-14	14/10/14	Genbank	Bombali	SL3.2.1
EBOV_20142843_KR653301_SLE_Bombali_2014-10-23	14/10/23	Genbank	Bombali	SL3.2.1
EBOV_20142856_KR653302_SLE_Tonkolili_2014-10-24	14/10/24	Genbank	Tonkolili	SL3.2.1
EBOV_20143107_KR653247_SLE_Bombali_2014-10-25	14/10/25	Genbank	Bombali	SL3.2.1
EBOV_J0096_KP759654_SLE_WesternUrban_2014-10-27	14/10/27	Genbank	WAU	SL3.2.1
EBOV_J0123_KP759672_SLE_WesternRural_2014-11-01	14/11/01	Genbank	WAR	SL3.2.1
EBOV_J0152_KP759614_SLE_WesternRural_2014-11-07	14/11/07	Genbank	WAR	SL3.2.1
EBOV_J0160_KP759621_SLE_WesternRural_2014-11-07	14/11/07	Genbank	WAR	SL3.2.1
EBOV_20144192_KR653238_SLE_Bombali_2014-11-08	14/11/08	Genbank	Bombali	SL3.2.1
EBOV_20144837_KR653259_SLE_Bombali_2014-11-14	14/11/14	Genbank	Bombali	SL3.2.1
NICD3208-Vero_SLE_WR_2014-11-14	14/11/14	NICD	WAR	SL3.2.1
NICD3294_SLE_WR_2014-11-14	14/11/14	NICD	WAR	SL3.2.1
NICD3608-Vero_SLE_WR_2014-11-21	14/11/21	NICD	WAR	SL3.2.1
NICD3693-Vero_SLE_WR_2014-11-23	14/11/23	NICD	WAR	SL3.2.1
EBOV_20145835_KR653295_SLE_Tonkolili_2014-11-25	14/11/25	Genbank	Tonkolili	SL3.2.1
EBOV_MK0024_KU296705_SLE_Bombali_2014-12-08	14/12/08	Genbank	Bombali	SL3.2.1
EBOV_MK0045_KU296776_SLE_Bombali_2014-12-09	14/12/09	Genbank	Bombali	SL3.2.1
NICD4513-Vero_SLE_WUR_2014-12-09	14/12/09	NICD	WAU	SL3.2.1
EBOV_MK0085_KU296738_SLE_Bombali_2014-12-10	14/12/10	Genbank	Bombali	SL3.2.1
NICD4691_SLE_WUR_2014-12-11	14/12/11	NICD	WAU	SL3.2.1
EBOV_KT2641_KU296709_SLE_UNKNOWN_2014-12-12	14/12/12	Genbank	UNKNOWN	SL3.2.1
NICD4670_SLE_WUR_2014-12-12	14/12/12	NICD	WAU	SL3.2.1
NICD4672_SLE_WUR_2014-12-12	14/12/12	NICD	WAU	SL3.2.1
NICD4692_SLE_WUR_2014-12-13	14/12/13	NICD	WAU	SL3.2.1
EBOV_KT2810_KU296778_SLE_UNKNOWN_2014-12-16	14/12/16	Genbank	UNKNOWN	SL3.2.1
EBOV_MK0364_KU296453_SLE_Bombali_2014-12-21	14/12/21	Genbank	Bombali	SL3.2.1

EBOV_MK0365_KU296406_SLE_Bombali_2014-12-21	14/12/21	Genbank	Bombali	SL3.2.1
EBOV_MK0366_KU296320_SLE_Bombali_2014-12-21	14/12/21	Genbank	Bombali	SL3.2.1
EBOV_MK0367_KU296770_SLE_Bombali_2014-12-21	14/12/21	Genbank	Bombali	SL3.2.1
EBOV_MK0368_KU296718_SLE_Bombali_2014-12-21	14/12/21	Genbank	Bombali	SL3.2.1
EBOV_MK0369_KU296413_SLE_Bombali_2014-12-21	14/12/21	Genbank	Bombali	SL3.2.1
EBOV_MK0363_KU296545_SLE_UNKNOWN_2014-12-21	14/12/21	Genbank	UNKNOWN	SL3.2.1
EBOV_MK0375_KU296606_SLE_Bombali_2014-12-22	14/12/22	Genbank	Bombali	SL3.2.1
EBOV_MK0379_KU296480_SLE_Bombali_2014-12-22	14/12/22	Genbank	Bombali	SL3.2.1
EBOV_MK0388_KU296630_SLE_Bombali_2014-12-22	14/12/22	Genbank	Bombali	SL3.2.1
EBOV_MK0390_KU296398_SLE_Bombali_2014-12-22	14/12/22	Genbank	Bombali	SL3.2.1
EBOV_MK0392_KU296691_SLE_Bombali_2014-12-22	14/12/22	Genbank	Bombali	SL3.2.1
EBOV_20146553_KR653224_SLE_Koinadugu_2014-12-22	14/12/22	Genbank	Koinadugu	SL3.2.1
EBOV_MK0393_KU296496_SLE_UNKNOWN_2014-12-22	14/12/22	Genbank	UNKNOWN	SL3.2.1
EBOV_MK0425_KU296379_SLE_Bombali_2014-12-23	14/12/23	Genbank	Bombali	SL3.2.1
EBOV_MK0431_KU296621_SLE_Bombali_2014-12-23	14/12/23	Genbank	Bombali	SL3.2.1
EBOV_MK0433_KU296845_SLE_Bombali_2014-12-23	14/12/23	Genbank	Bombali	SL3.2.1
EBOV_MK0434_KU296554_SLE_Bombali_2014-12-23	14/12/23	Genbank	Bombali	SL3.2.1
EBOV_MK0491_KU296510_SLE_Bombali_2014-12-23	14/12/23	Genbank	Bombali	SL3.2.1
EBOV_MK0531_KU296448_SLE_Bombali_2014-12-26	14/12/26	Genbank	Bombali	SL3.2.1
EBOV_MK0533_KU296436_SLE_UNKNOWN_2014-12-26	14/12/26	Genbank	UNKNOWN	SL3.2.1
EBOV_MK0549_KU296825_SLE_Bombali_2014-12-27	14/12/27	Genbank	Bombali	SL3.2.1
EBOV_MK0551_KU296717_SLE_UNKNOWN_2014-12-27	14/12/27	Genbank	UNKNOWN	SL3.2.1
EBOV_MK0584_KU296339_SLE_Bombali_2014-12-28	14/12/28	Genbank	Bombali	SL3.2.1
EBOV_MK0575_KU296681_SLE_UNKNOWN_2014-12-28	14/12/28	Genbank	UNKNOWN	SL3.2.1
EBOV_MK0607_KU296335_SLE_UNKNOWN_2014-12-28	14/12/28	Genbank	UNKNOWN	SL3.2.1
EBOV_MK0664_KU296761_SLE_UNKNOWN_2014-12-30	14/12/30	Genbank	UNKNOWN	SL3.2.1
EBOV_MK0709_KU296373_SLE_Tonkolili_2014-12-31	14/12/31	Genbank	Tonkolili	SL3.2.1
EBOV_MK0723_KU296610_SLE__2015-01-01	15/01/01	Genbank	UNKNOWN	SL3.2.1
EBOV_MK0766_KU296777_SLE_UNKNOWN_2015-01-02	15/01/02	Genbank	UNKNOWN	SL3.2.1
EBOV_MK0869_KU296534_SLE_Bombali_2015-01-05	15/01/05	Genbank	Bombali	SL3.2.1
EBOV_MK0849_KU296323_SLE_UNKNOWN_2015-01-05	15/01/05	Genbank	UNKNOWN	SL3.2.1
EBOV_MK0857_KU296789_SLE_UNKNOWN_2015-01-05	15/01/05	Genbank	UNKNOWN	SL3.2.1
EBOV_MK0868_KU296537_SLE_UNKNOWN_2015-01-05	15/01/05	Genbank	UNKNOWN	SL3.2.1
EBOV_MK0966_KU296507_SLE_Bombali_2015-01-08	15/01/08	Genbank	Bombali	SL3.2.1

EBOV_MK1000_KU296384_SLE_Bombali_2015-01-10	15/01/10	Genbank	Bombali	SL3.2.1
EBOV_MK1014_KU296316_SLE_Bombali_2015-01-10	15/01/10	Genbank	Bombali	SL3.2.1
EBOV_MK1036_KU296834_SLE_Bombali_2015-01-11	15/01/11	Genbank	Bombali	SL3.2.1
EBOV_KT4054_KU296429_SLE_UNKNOWN_2015-01-13	15/01/13	Genbank	UNKNOWN	SL3.2.1
EBOV_MK1197_KU296301_SLE_Bombali_2015-01-17	15/01/17	Genbank	Bombali	SL3.2.1
EBOV_MK1202_KU296581_SLE_Bombali_2015-01-17	15/01/17	Genbank	Bombali	SL3.2.1
EBOV_MK1320_KU296354_SLE_Bombali_2015-01-20	15/01/20	Genbank	Bombali	SL3.2.1
EBOV_466_EMLH_KU296433_SLE_WesternUrban_2015-01-28	15/01/28	Genbank	WAU	SL3.2.1
EBOV_PL2921_KU296506_SLE_Kambia_2015-02-03	15/02/03	Genbank	Kambia	SL3.2.1
EBOV_MK1782_KU296792_SLE_UNKNOWN_2015-02-06	15/02/06	Genbank	UNKNOWN	SL3.2.1
EBOV_MK1888_KU296708_SLE_UNKNOWN_2015-02-09	15/02/09	Genbank	UNKNOWN	SL3.2.1
EBOV_KT4801_KU296722_SLE_UNKNOWN_2015-02-10	15/02/10	Genbank	UNKNOWN	SL3.2.1
EBOV_MK4165_KU296575_SLE_Koinadugu_2015-04-16	15/04/16	Genbank	Koinadugu	SL3.2.1
EBOV_G4956_KR105282_SLE_Tonkolili_2014-08-13	14/08/13	Genbank	Tonkolili	SL3.2.2
EBOV_G4972_KR105285_SLE_Kenema_2014-08-14	14/08/14	Genbank	Kenema	SL3.2.2
NICD0108_SLE_PL_2014-08-30	14/08/30	NICD	Port Loko	SL3.2.2
NICD0104_SLE_WR_2014-08-30	14/08/30	NICD	WAR	SL3.2.2
EBOV_20140433_KR653246_SLE_Tonkolili_2014-09-03	14/09/03	Genbank	Tonkolili	SL3.2.2
NICD0338_SLE_PL_2014-09-08	14/09/08	NICD	Port Loko	SL3.2.2
NICD0424_SLE_TONK_2014-09-10	14/09/10	NICD	Tonkolili	SL3.2.2
NICD0720-Vero_SLE_PL_2014-09-16	14/09/16	NICD	Port Loko	SL3.2.2
NICD0721_SLE_PL_2014-09-16	14/09/16	NICD	Port Loko	SL3.2.2
NICD0878_SLE_PL_2014-09-20	14/09/20	NICD	Port Loko	SL3.2.2
NICD0894_SLE_PL_2014-09-20	14/09/20	NICD	Port Loko	SL3.2.2
NICD0899_SLE_PL_2014-09-20	14/09/20	NICD	Port Loko	SL3.2.2
NICD0900_SLE_PL_2014-09-20	14/09/20	NICD	Port Loko	SL3.2.2
NICD1049_SLE_PL_2014-09-21	14/09/21	NICD	Port Loko	SL3.2.2
EBOV_20141288_KR653232_SLE_PortLoko_2014-09-23	14/09/23	Genbank	Port Loko	SL3.2.2
NICD1396_SLE_PL_2014-09-25	14/09/25	NICD	Port Loko	SL3.2.2
EBOV_G5982_KR105332_SLE_UNKNOWN_2014-09-25	14/09/25	Genbank	UNKNOWN	SL3.2.2
EBOV_G5986_KR105335_SLE_UNKNOWN_2014-09-25	14/09/25	Genbank	UNKNOWN	SL3.2.2
EBOV_G5988_KR105336_SLE_UNKNOWN_2014-09-25	14/09/25	Genbank	UNKNOWN	SL3.2.2
EBOV_J0013_KP759643_SLE_PortLoko_2014-09-28	14/09/28	Genbank	Port Loko	SL3.2.2
EBOV_J0011_KP759641_SLE_PortLoko_2014-09-29	14/09/29	Genbank	Port Loko	SL3.2.2

EBOV_J0012_KP759642_SLE_PortLoko_2014-09-29	14/09/29	Genbank	Port Loko	SL3.2.2
EBOV_J0014_KP759740_SLE_PortLoko_2014-09-29	14/09/29	Genbank	Port Loko	SL3.2.2
EBOV_J0029_KP759688_SLE_PortLoko_2014-09-29	14/09/29	Genbank	Port Loko	SL3.2.2
EBOV_J0018_KP759644_SLE_WesternRural_2014-09-30	14/09/30	Genbank	WAR	SL3.2.2
EBOV_J0030_KP759606_SLE_PortLoko_2014-10-03	14/10/03	Genbank	Port Loko	SL3.2.2
EBOV_J0049_KP759717_SLE_PortLoko_2014-10-07	14/10/07	Genbank	Port Loko	SL3.2.2
EBOV_J0061_KP759637_SLE_PortLoko_2014-10-10	14/10/10	Genbank	Port Loko	SL3.2.2
EBOV_J0073_KP759738_SLE_PortLoko_2014-10-12	14/10/12	Genbank	Port Loko	SL3.2.2
EBOV_20143036_KR653285_SLE_PortLoko_2014-10-24	14/10/24	Genbank	Port Loko	SL3.2.2
EBOV_J0114_KP759596_SLE_PortLoko_2014-10-29	14/10/29	Genbank	Port Loko	SL3.2.2
EBOV_J0115_KP759597_SLE_PortLoko_2014-10-29	14/10/29	Genbank	Port Loko	SL3.2.2
EBOV_J0140_KP759603_SLE_WesternRural_2014-10-30	14/10/30	Genbank	WAR	SL3.2.2
EBOV_J0142_KP759690_SLE_WesternUrban_2014-11-03	14/11/03	Genbank	WAU	SL3.2.2
EBOV_J0163_KP759702_SLE_PortLoko_2014-11-08	14/11/08	Genbank	Port Loko	SL3.2.2
NICD2974-swab_SLE_PL_2014-11-08	14/11/08	NICD	Port Loko	SL3.2.2
EBOV_J0164_KP759623_SLE_WesternRural_2014-11-09	14/11/09	Genbank	WAR	SL3.2.2
NICD3097_SLE_PL_2014-11-12	14/11/12	NICD	Port Loko	SL3.2.2
EBOV_Makona-GE1_KP728283_SLE_WesternArea_2014-11-21	14/11/21	Genbank	Western Area	SL3.2.2
EBOV_KT2315_KU296812_SLE_PortLoko_2014-12-02	14/12/02	Genbank	Port Loko	SL3.2.2
EBOV_KT2316_KU296774_SLE_PortLoko_2014-12-02	14/12/02	Genbank	Port Loko	SL3.2.2
NICD4795-swab_SLE_WUR_2014-12-14	14/12/14	NICD	WAU	SL3.2.2
NICD5367-swab_SLE_WUR_2014-12-27	14/12/27	NICD	WAU	SL3.2.2
EBOV_77581_EMLK_KU296540_SLE_WesternUrban_2015-01-03	15/01/03	Genbank	WAU	SL3.2.2
EBOV_77582_EMLK_KU296816_SLE_WesternUrban_2015-01-03	15/01/03	Genbank	WAU	SL3.2.2
EBOV_77594_EMLK_KU296343_SLE_WesternUrban_2015-01-04	15/01/04	Genbank	WAU	SL3.2.2
EBOV_77724_EMLK_KU296656_SLE_UNKNOWN_2015-01-10	15/01/10	Genbank	UNKNOWN	SL3.2.2
EBOV_77706_EMLK_KU296572_SLE_WesternUrban_2015-01-10	15/01/10	Genbank	WAU	SL3.2.2
EBOV_75688_EMLH_KU296672_SLE_WesternRural_2015-01-14	15/01/14	Genbank	WAR	SL3.2.2
EBOV_75750_EMLH_KU296464_SLE_WesternUrban_2015-01-17	15/01/17	Genbank	WAU	SL3.2.2
NICD6079_SLE_WUR_2015-01-17	15/01/17	NICD	WAU	SL3.2.2
EBOV_375_EMLH_KU296813_SLE_UNKNOWN_2015-01-21	15/01/21	Genbank	UNKNOWN	SL3.2.2
EBOV_362_EMLH_KU296505_SLE_WesternUrban_2015-01-21	15/01/21	Genbank	WAU	SL3.2.2
EBOV_KT5243_KU296736_SLE_UNKNOWN_2015-02-22	15/02/22	Genbank	UNKNOWN	SL3.2.2

EBOV_PL4028_KU296552_SLE_PortLoko_2015-02-26	15/02/26	Genbank	Port Loko	SL3.2.2
EBOV_PL4121_KU296305_SLE_PortLoko_2015-02-28	15/02/28	Genbank	Port Loko	SL3.2.2
EBOV_PL4123_KU296752_SLE_PortLoko_2015-02-28	15/02/28	Genbank	Port Loko	SL3.2.2
EBOV_PL4125_KU296471_SLE_PortLoko_2015-02-28	15/02/28	Genbank	Port Loko	SL3.2.2
EBOV_PL4126_KU296478_SLE_PortLoko_2015-02-28	15/02/28	Genbank	Port Loko	SL3.2.2
EBOV_PL4127_KU296529_SLE_PortLoko_2015-02-28	15/02/28	Genbank	Port Loko	SL3.2.2
EBOV_PL4169_KU296828_SLE_PortLoko_2015-03-01	15/03/01	Genbank	Port Loko	SL3.2.2
EBOV_PL4256_KU296509_SLE_PortLoko_2015-03-03	15/03/03	Genbank	Port Loko	SL3.2.2
EBOV_PL4322_KU296677_SLE_PortLoko_2015-03-05	15/03/05	Genbank	Port Loko	SL3.2.2
EBOV_PL4330_KU296624_SLE_PortLoko_2015-03-05	15/03/05	Genbank	Port Loko	SL3.2.2
EBOV_PL4561_KU296725_SLE_PortLoko_2015-03-10	15/03/10	Genbank	Port Loko	SL3.2.2
EBOV_PL4784_KU296660_SLE_PortLoko_2015-03-14	15/03/14	Genbank	Port Loko	SL3.2.2
EBOV_PL5201_KU296612_SLE_PortLoko_2015-03-23	15/03/23	Genbank	Port Loko	SL3.2.2
EBOV_G4725_KR105267_SLE_Moyamba_2014-08-04	14/08/04	Genbank	Moyamba	SL3.2.3
EBOV_G4955_KR105281_SLE_UNKNOWN_2014-08-13	14/08/13	Genbank	UNKNOWN	SL3.2.3
EBOV_G4999_KR105291_SLE_Kenema_2014-08-15	14/08/15	Genbank	Kenema	SL3.2.3
EBOV_G4994_KR105289_SLE_UNKNOWN_2014-08-15	14/08/15	Genbank	UNKNOWN	SL3.2.3
EBOV_G5064_KR105297_SLE_Moyamba_2014-08-17	14/08/17	Genbank	Moyamba	SL3.2.3
EBOV_20140091_KR653239_SLE_WesternRural_2014-08-22	14/08/22	Genbank	WAR	SL3.2.3
NICD0013_SLE_WR_2014-08-26	14/08/26	NICD	WAR	SL3.2.3
NICD0032_SLE_MOY_2014-08-27	14/08/27	NICD	Moyamba	SL3.2.3
NICD0110_SLE_PL_2014-08-30	14/08/30	NICD	Port Loko	SL3.2.3
EBOV_20140436_KR653287_SLE_Moyamba_2014-09-03	14/09/03	Genbank	Moyamba	SL3.2.3
EBOV_G5516_KR105307_SLE_Moyamba_2014-09-04	14/09/04	Genbank	Moyamba	SL3.2.3
EBOV_G5520_KR105308_SLE_Kenema_2014-09-05	14/09/05	Genbank	Kenema	SL3.2.3
NICD0298_SLE_PL_2014-09-05	14/09/05	NICD	Port Loko	SL3.2.3
EBOV_G5617_KR105312_SLE_Kenema_2014-09-10	14/09/10	Genbank	Kenema	SL3.2.3
EBOV_G5691_KR105318_SLE_Kenema_2014-09-14	14/09/14	Genbank	Kenema	SL3.2.3
EBOV_G5738_KR105322_SLE_Moyamba_2014-09-15	14/09/15	Genbank	Moyamba	SL3.2.3
NICD0856-Vero_SLE_WR_2014-09-20	14/09/20	NICD	WAR	SL3.2.3
EBOV_G5853_KR105329_SLE_Moyamba_2014-09-21	14/09/21	Genbank	Moyamba	SL3.2.3
NICD1051_SLE_PL_2014-09-21	14/09/21	NICD	Port Loko	SL3.2.3
NICD1043_SLE_PL_2014-09-22	14/09/22	NICD	Port Loko	SL3.2.3
EBOV_20141282_KR653229_SLE_Kambia_2014-09-23	14/09/23	Genbank	Kambia	SL3.2.3

EBOV_G5985_KR105334_SLE_UNKNOWN_2014-09-25	14/09/25	Genbank	UNKNOWN	SL3.2.3
EBOV_J0003_KP759651_SLE_PortLoko_2014-09-28	14/09/28	Genbank	Port Loko	SL3.2.3
EBOV_J0032_KP759691_SLE_PortLoko_2014-09-30	14/09/30	Genbank	Port Loko	SL3.2.3
EBOV_J0038_KP759618_SLE_PortLoko_2014-09-30	14/09/30	Genbank	Port Loko	SL3.2.3
EBOV_20141491_KR653289_SLE_Kambia_2014-10-01	14/10/01	Genbank	Kambia	SL3.2.3
EBOV_J0048_KP759716_SLE_WesternRural_2014-10-05	14/10/05	Genbank	WAR	SL3.2.3
EBOV_J0074_KP759739_SLE_WesternRural_2014-10-13	14/10/13	Genbank	WAR	SL3.2.3
EBOV_J0079_KP759748_SLE_WesternRural_2014-10-16	14/10/16	Genbank	WAR	SL3.2.3
EBOV_J0085_KP759645_SLE_PortLoko_2014-10-17	14/10/17	Genbank	Port Loko	SL3.2.3
EBOV_J0088_KP759648_SLE_PortLoko_2014-10-20	14/10/20	Genbank	Port Loko	SL3.2.3
NICD2521-swab_SLE_WUR_2014-10-23	14/10/23	NICD	WAU	SL3.2.3
EBOV_J0102_KP759657_SLE_PortLoko_2014-10-27	14/10/27	Genbank	Port Loko	SL3.2.3
EBOV_J0101_KP759656_SLE_PortLoko_2014-10-29	14/10/29	Genbank	Port Loko	SL3.2.3
EBOV_J0103_KP759658_SLE_PortLoko_2014-10-29	14/10/29	Genbank	Port Loko	SL3.2.3
EBOV_J0104_KP759765_SLE_PortLoko_2014-10-29	14/10/29	Genbank	Port Loko	SL3.2.3
EBOV_J0105_KP759766_SLE_PortLoko_2014-10-29	14/10/29	Genbank	Port Loko	SL3.2.3
EBOV_J0118_KP759598_SLE_WesternRural_2014-10-31	14/10/31	Genbank	WAR	SL3.2.3
EBOV_J0141_KP759689_SLE_WesternRural_2014-11-04	14/11/04	Genbank	WAR	SL3.2.3
EBOV_J0165_KP759703_SLE_WesternUrban_2014-11-08	14/11/08	Genbank	WAU	SL3.2.3
NICD2965-Vero_SLE_PL_2014-11-10	14/11/10	NICD	Port Loko	SL3.2.3
EBOV_J0168_KP759705_SLE_WesternArea_2014-11-11	14/11/11	Genbank	Western Area	SL3.2.3
EBOV_20144865_KR653231_SLE_PortLoko_2014-11-13	14/11/13	Genbank	Port Loko	SL3.2.3
NICD3670_SLE_WR_2014-11-22	14/11/22	NICD	WAR	SL3.2.3
EBOV_G4861_KR105274_SLE_UNKNOWN_2014-08-10	14/08/10	Genbank	UNKNOWN	SL3.2.4
EBOV_G5114_KR105299_SLE_Pujehun_2014-08-18	14/08/18	Genbank	Pujehun	SL3.2.4
EBOV_G5112_KR105298_SLE_Kenema_2014-08-19	14/08/19	Genbank	Kenema	SL3.2.4
EBOV_G5119_KR105300_SLE_Kenema_2014-08-24	14/08/24	Genbank	Kenema	SL3.2.4
NICD0019_SLE_WR_2014-08-26	14/08/26	NICD	WAR	SL3.2.4
EBOV_20140395_KR653279_SLE_Kono_2014-09-02	14/09/02	Genbank	Kono	SL3.2.4
EBOV_G5529_KR105309_SLE_Kono_2014-09-05	14/09/05	Genbank	Kono	SL3.2.4
EBOV_G5684_KR105316_SLE_Kenema_2014-09-13	14/09/13	Genbank	Kenema	SL3.2.4
EBOV_G5685_KR105317_SLE_Kenema_2014-09-13	14/09/13	Genbank	Kenema	SL3.2.4
EBOV_G5743_KR105323_SLE_Kono_2014-09-17	14/09/17	Genbank	Kono	SL3.2.4

EBOV_20141123_KR653291_SLE_Bo_2014-09-22	14/09/22	Genbank	Bo	SL3.2.4
EBOV_G5898_KR105331_SLE_Bo_2014-09-22	14/09/22	Genbank	Bo	SL3.2.4
EBOV_20141280_KR653276_SLE_Kambia_2014-09-23	14/09/23	Genbank	Kambia	SL3.2.4
EBOV_J0004_KP759668_SLE_WesternRural_2014-09-28	14/09/28	Genbank	WAR	SL3.2.4
EBOV_J0006_KP759630_SLE_WesternRural_2014-09-28	14/09/28	Genbank	WAR	SL3.2.4
NICD1526_SLE_WR_2014-10-01	14/10/01	NICD	WAR	SL3.2.4
EBOV_J0026_KP759670_SLE_Bombali_2014-10-02	14/10/02	Genbank	Bombali	SL3.2.4
EBOV_20141582_KR653230_SLE_Bo_2014-10-04	14/10/04	Genbank	Bo	SL3.2.4
EBOV_J0040_KP759629_SLE_WesternArea_2014-10-04	14/10/04	Genbank	Western Area	SL3.2.4
EBOV_J0043_KP759712_SLE_WesternArea_2014-10-04	14/10/04	Genbank	Western Area	SL3.2.4
EBOV_20141650_KR653225_SLE_Kenema_2014-10-05	14/10/05	Genbank	Kenema	SL3.2.4
EBOV_J0044_KP759713_SLE_WesternRural_2014-10-05	14/10/05	Genbank	WAR	SL3.2.4
EBOV_J0045_KP759714_SLE_WesternUrban_2014-10-05	14/10/05	Genbank	WAU	SL3.2.4
EBOV_J0047_KP759632_SLE_WesternUrban_2014-10-05	14/10/05	Genbank	WAU	SL3.2.4
EBOV_J0060_KP759726_SLE_WesternUrban_2014-10-06	14/10/06	Genbank	WAU	SL3.2.4
EBOV_J0059_KP759725_SLE_WesternRural_2014-10-08	14/10/08	Genbank	WAR	SL3.2.4
EBOV_J0052_KP759720_SLE_WesternUrban_2014-10-08	14/10/08	Genbank	WAU	SL3.2.4
EBOV_J0065_KP759729_SLE_WesternRural_2014-10-09	14/10/09	Genbank	WAR	SL3.2.4
EBOV_J0068_KP759732_SLE_WesternRural_2014-10-09	14/10/09	Genbank	WAR	SL3.2.4
EBOV_J0069_KP759733_SLE_WesternRural_2014-10-09	14/10/09	Genbank	WAR	SL3.2.4
EBOV_J0064_KP759728_SLE_WesternUrban_2014-10-09	14/10/09	Genbank	WAU	SL3.2.4
NICD1972-Vero_SLE_WUR_2014-10-09	14/10/09	NICD	WAU	SL3.2.4
NICD2118_SLE_WUR_2014-10-11	14/10/11	NICD	WAU	SL3.2.4
EBOV_J0081_KP759750_SLE_WesternRural_2014-10-16	14/10/16	Genbank	WAR	SL3.2.4
EBOV_J0075_KP759743_SLE_WesternRural_2014-10-17	14/10/17	Genbank	WAR	SL3.2.4
EBOV_J0077_KP759745_SLE_WesternRural_2014-10-17	14/10/17	Genbank	WAR	SL3.2.4
EBOV_J0080_KP759749_SLE_PortLoko_2014-10-18	14/10/18	Genbank	Port Loko	SL3.2.4
EBOV_J0078_KP759746_SLE_WesternUrban_2014-10-18	14/10/18	Genbank	WAU	SL3.2.4
NICD2540-Vero_SLE_WUR_2014-10-22	14/10/22	NICD	WAU	SL3.2.4
NICD2542_SLE_WUR_2014-10-22	14/10/22	NICD	WAU	SL3.2.4
EBOV_J0087_KP759647_SLE_PortLoko_2014-10-23	14/10/23	Genbank	Port Loko	SL3.2.4
EBOV_J0086_KP759646_SLE_WesternRural_2014-10-23	14/10/23	Genbank	WAR	SL3.2.4

EBOV_J0090_KP759649_SLE_WesternUrban_2014-10-23	14/10/23	Genbank	WAU	SL3.2.4
EBOV_J0093_KP759760_SLE_WesternRural_2014-10-24	14/10/24	Genbank	WAR	SL3.2.4
EBOV_J0091_KP759759_SLE_WesternArea_2014-10-25	14/10/25	Genbank	Western Area	SL3.2.4
EBOV_20143031_KR653275_SLE_WesternUrban_2014-10-25	14/10/25	Genbank	WAU	SL3.2.4
EBOV_J0100_KP759655_SLE_Kambia_2014-10-26	14/10/26	Genbank	Kambia	SL3.2.4
EBOV_J0094_KP759761_SLE_WesternRural_2014-10-27	14/10/27	Genbank	WAR	SL3.2.4
EBOV_20143187_KR653298_SLE_Pujehun_2014-10-28	14/10/28	Genbank	Pujehun	SL3.2.4
EBOV_J0098_KP759763_SLE_WesternRural_2014-10-28	14/10/28	Genbank	WAR	SL3.2.4
EBOV_J0106_KP759767_SLE_WesternRural_2014-10-28	14/10/28	Genbank	WAR	SL3.2.4
EBOV_J0108_KP759659_SLE_WesternRural_2014-10-28	14/10/28	Genbank	WAR	SL3.2.4
EBOV_J0107_KP759768_SLE_WesternUrban_2014-10-29	14/10/29	Genbank	WAU	SL3.2.4
EBOV_J0116_KP759664_SLE_WesternRural_2014-10-30	14/10/30	Genbank	WAR	SL3.2.4
EBOV_J0117_KP759665_SLE_WesternRural_2014-10-31	14/10/31	Genbank	WAR	SL3.2.4
EBOV_J0133_KP759681_SLE_WesternRural_2014-10-31	14/10/31	Genbank	WAR	SL3.2.4
EBOV_J0136_KP759684_SLE_WesternRural_2014-11-01	14/11/01	Genbank	WAR	SL3.2.4
EBOV_J0124_KP759673_SLE_WesternArea_2014-11-02	14/11/02	Genbank	Western Area	SL3.2.4
EBOV_J0129_KP759600_SLE_WesternUrban_2014-11-02	14/11/02	Genbank	WAU	SL3.2.4
EBOV_J0132_KP759601_SLE_WesternUrban_2014-11-02	14/11/02	Genbank	WAU	SL3.2.4
EBOV_20143716_KR653292_SLE_Moyamba_2014-11-04	14/11/04	Genbank	Moyamba	SL3.2.4
EBOV_20143796_KR653257_SLE_Moyamba_2014-11-05	14/11/05	Genbank	Moyamba	SL3.2.4
EBOV_20143753_KR653236_SLE_Tonkolili_2014-11-05	14/11/05	Genbank	Tonkolili	SL3.2.4
NICD3824_SLE_WR_2014-11-25	14/11/05	NICD	WAR	SL3.2.4
NICD2749_SLE_KMB_2014-11-06	14/11/06	NICD	Kambia	SL3.2.4
EBOV_J0148_KP759612_SLE_WesternRural_2014-11-06	14/11/06	Genbank	WAR	SL3.2.4
EBOV_J0151_KP759613_SLE_WesternRural_2014-11-07	14/11/07	Genbank	WAR	SL3.2.4
EBOV_J0153_KP759616_SLE_WesternRural_2014-11-07	14/11/07	Genbank	WAR	SL3.2.4
EBOV_J0167_KP759624_SLE_WesternUrban_2014-11-08	14/11/08	Genbank	WAU	SL3.2.4
EBOV_J0169_KP759706_SLE_WesternUrban_2014-11-09	14/11/09	Genbank	WAU	SL3.2.4
EBOV_J0170_KP759625_SLE_WesternUrban_2014-11-10	14/11/10	Genbank	WAU	SL3.2.4
EBOV_J0172_KP759626_SLE_WesternRural_2014-11-11	14/11/11	Genbank	WAR	SL3.2.4
EBOV_20144610_KR653270_SLE_Tonkolili_2014-11-12	14/11/12	Genbank	Tonkolili	SL3.2.4
NICD3465-swab_SLE_WUR_2014-11-18	14/11/18	NICD	WAU	SL3.2.4

NICD3666_SLE_WR_2014-11-22	14/11/22	NICD	WAR	SL3.2.4
NICD3687-Vero_SLE_WUR_2014-11-23	14/11/23	NICD	WAU	SL3.2.4
NICD3752_SLE_WR_2014-11-24	14/11/24	NICD	WAR	SL3.2.4
NICD3753_SLE_WR_2014-11-24	14/11/24	NICD	WAR	SL3.2.4
EBOV_20145853_KR653290_SLE_Pujehun_2014-11-26	14/11/26	Genbank	Pujehun	SL3.2.4
NICD3944_SLE_WUR_2014-11-26	14/11/26	NICD	WAU	SL3.2.4
NICD3954_SLE_WUR_2014-11-26	14/11/26	NICD	WAU	SL3.2.4
NICD4024-Vero_SLE_WUR_2014-11-29	14/11/29	NICD	WAU	SL3.2.4
NICD4027-Vero_SLE_WUR_2014-11-29	14/11/29	NICD	WAU	SL3.2.4
NICD4029-Vero_SLE_WUR_2014-11-29	14/11/29	NICD	WAU	SL3.2.4
NICD4041-Vero_SLE_WUR_2014-11-29	14/11/29	NICD	WAU	SL3.2.4
EBOV_KT2317_KU296735_SLE_PortLoko_2014-12-02	14/12/02	Genbank	Port Loko	SL3.2.4
EBOV_KT2321_KU296762_SLE_PortLoko_2014-12-02	14/12/02	Genbank	Port Loko	SL3.2.4
NICD4122_SLE_WR_2014-12-02	14/12/02	NICD	WAR	SL3.2.4
NICD4125_SLE_WR_2014-12-02	14/12/02	NICD	WAR	SL3.2.4
NICD4167_SLE_WUR_2014-12-02	14/12/02	NICD	WAU	SL3.2.4
NICD4325_SLE_WR_2014-12-05	14/12/05	NICD	WAR	SL3.2.4
NICD4361-Vero_SLE_WR_2014-12-06	14/12/06	NICD	WAR	SL3.2.4
NICD4430_SLE_WUR_2014-12-06	14/12/06	NICD	WAU	SL3.2.4
NICD4693_SLE_WUR_2014-12-10	14/12/10	NICD	WAU	SL3.2.4
NICD4674_SLE_WR_2014-12-12	14/12/12	NICD	WAR	SL3.2.4
NICD4735_SLE_WUR_2014-12-13	14/12/13	NICD	WAU	SL3.2.4
NICD4823_SLE_WUR_2014-12-15	14/12/15	NICD	WAU	SL3.2.4
NICD4877_SLE_WUR_2014-12-16	14/12/16	NICD	WAU	SL3.2.4
NICD4932-Vero_SLE_WUR_2014-12-17	14/12/17	NICD	WAU	SL3.2.4
NICD4975_SLE_WUR_2014-12-18	14/12/18	NICD	WAU	SL3.2.4
NICD5071_SLE_WUR_2014-12-20	14/12/20	NICD	WAU	SL3.2.4
NICD5400_SLE_WUR_2014-12-28	14/12/28	NICD	WAU	SL3.2.4
EBOV_Makona-UK2_KP658432_SLE_WesternArea_2014-12-29	14/12/29	Genbank	Western Area	SL3.2.4
NICD5386_SLE_WUR_2014-12-29	14/12/29	NICD	WAU	SL3.2.4
NICD5388_SLE_WUR_2014-12-29	14/12/29	NICD	WAU	SL3.2.4
NICD5395_SLE_WUR_2014-12-29	14/12/29	NICD	WAU	SL3.2.4
EBOV_77525_EMLK_KU296820_SLE__2014-12-30	14/12/30	Genbank	UNKNOWN	SL3.2.4

EBOV_77526_EMLK_KU296824_SLE__2014-12-30	14/12/30	Genbank	UNKNOWN	SL3.2.4
EBOV_77549_EMLK_KU296293_SLE__2015-01-01	15/01/01	Genbank	UNKNOWN	SL3.2.4
EBOV_77558_EMLK_KU296733_SLE__2015-01-02	15/01/02	Genbank	UNKNOWN	SL3.2.4
EBOV_77563_EMLK_KU296432_SLE__2015-01-02	15/01/02	Genbank	UNKNOWN	SL3.2.4
EBOV_77574_EMLK_KU296700_SLE__2015-01-03	15/01/03	Genbank	UNKNOWN	SL3.2.4
EBOV_77573_EMLK_KU296516_SLE_WesternUrban_2015-01-03	15/01/03	Genbank	WAU	SL3.2.4
EBOV_77580_EMLK_KU296431_SLE_WesternUrban_2015-01-03	15/01/03	Genbank	WAU	SL3.2.4
NICD5647-Vero_SLE_WUR_2015-01-03	15/01/03	NICD	WAU	SL3.2.4
EBOV_77589_EMLK_KU296403_SLE_WesternUrban_2015-01-04	15/01/04	Genbank	WAU	SL3.2.4
EBOV_77612_EMLK_KU296636_SLE_WesternUrban_2015-01-05	15/01/05	Genbank	WAU	SL3.2.4
EBOV_77613_EMLK_KU296579_SLE_WesternUrban_2015-01-05	15/01/05	Genbank	WAU	SL3.2.4
EBOV_77615_EMLK_KU296706_SLE_WesternUrban_2015-01-05	15/01/05	Genbank	WAU	SL3.2.4
EBOV_77630_EMLK_KU296779_SLE_WesternUrban_2015-01-06	15/01/06	Genbank	WAU	SL3.2.4
EBOV_77632_EMLK_KU296710_SLE_WesternUrban_2015-01-06	15/01/06	Genbank	WAU	SL3.2.4
EBOV_77643_EMLK_KU296395_SLE_WesternUrban_2015-01-07	15/01/07	Genbank	WAU	SL3.2.4
EBOV_77644_EMLK_KU296312_SLE_WesternUrban_2015-01-07	15/01/07	Genbank	WAU	SL3.2.4
EBOV_77648_EMLK_KU296620_SLE_WesternUrban_2015-01-07	15/01/07	Genbank	WAU	SL3.2.4
EBOV_77649_EMLK_KU296380_SLE_WesternUrban_2015-01-07	15/01/07	Genbank	WAU	SL3.2.4
EBOV_77667_EMLK_KU296361_SLE__2015-01-08	15/01/08	Genbank	UNKNOWN	SL3.2.4
EBOV_77669_EMLK_KU296367_SLE__2015-01-08	15/01/08	Genbank	UNKNOWN	SL3.2.4
EBOV_77670_EMLK_KU296329_SLE__2015-01-08	15/01/08	Genbank	UNKNOWN	SL3.2.4
EBOV_77673_EMLK_KU296458_SLE__2015-01-08	15/01/08	Genbank	UNKNOWN	SL3.2.4
EBOV_77675_EMLK_KU296807_SLE__2015-01-08	15/01/08	Genbank	UNKNOWN	SL3.2.4
EBOV_77676_EMLK_KU296309_SLE__2015-01-08	15/01/08	Genbank	UNKNOWN	SL3.2.4
EBOV_77677_EMLK_KU296558_SLE__2015-01-08	15/01/08	Genbank	UNKNOWN	SL3.2.4
EBOV_77679_EMLK_KU296512_SLE__2015-01-08	15/01/08	Genbank	UNKNOWN	SL3.2.4
EBOV_77681_EMLK_KU296435_SLE_UNKNOWN_2015-01-08	15/01/08	Genbank	UNKNOWN	SL3.2.4
NICD5747_SLE_WUR_2015-01-08	15/01/08	NICD	WAU	SL3.2.4
NICD5819_SLE_WUR_2015-01-09	15/01/09	NICD	WAU	SL3.2.4
EBOV_77710_EMLK_KU296696_SLE_WesternUrban_2015-01-10	15/01/10	Genbank	WAU	SL3.2.4
EBOV_77711_EMLK_KU296644_SLE_WesternUrban_2015-01-10	15/01/10	Genbank	WAU	SL3.2.4
EBOV_77712_EMLK_KU296400_SLE_WesternUrban_2015-01-10	15/01/10	Genbank	WAU	SL3.2.4
EBOV_77716_EMLK_KU296569_SLE_WesternUrban_2015-01-10	15/01/10	Genbank	WAU	SL3.2.4
EBOV_75685_EMLH_KU296365_SLE_UNKNOWN_2015-01-14	15/01/14	Genbank	UNKNOWN	SL3.2.4

EBOV_75708_EMLH_KU296739_SLE__2015-01-15	15/01/15	Genbank	UNKNOWN	SL3.2.4
EBOV_75722_EMLH_KU296791_SLE_WesternRural_2015-01-15	15/01/15	Genbank	WAR	SL3.2.4
EBOV_PL2101_KU296714_SLE_PortLoko_2015-01-18	15/01/18	Genbank	Port Loko	SL3.2.4
EBOV_75779_EMLH_KU296407_SLE_WesternUrban_2015-01-19	15/01/19	Genbank	WAU	SL3.2.4
EBOV_356_EMLH_KU296546_SLE_UNKNOWN_2015-01-21	15/01/21	Genbank	UNKNOWN	SL3.2.4
EBOV_380_EMLH_KU296377_SLE_UNKNOWN_2015-01-22	15/01/22	Genbank	UNKNOWN	SL3.2.4
EBOV_389_EMLH_KU296593_SLE_WesternRural_2015-01-23	15/01/23	Genbank	WAR	SL3.2.4
EBOV_402_EMLH_KU296495_SLE_WesternRural_2015-01-23	15/01/23	Genbank	WAR	SL3.2.4
NICD6195_SLE_WUR_2015-01-23	15/01/23	NICD	WAU	SL3.2.4
EBOV_405_EMLH_KU296805_SLE_UNKNOWN_2015-01-24	15/01/24	Genbank	UNKNOWN	SL3.2.4
EBOV_408_EMLH_KU296408_SLE_UNKNOWN_2015-01-24	15/01/24	Genbank	UNKNOWN	SL3.2.4
EBOV_413_EMLH_KU296472_SLE_UNKNOWN_2015-01-24	15/01/24	Genbank	UNKNOWN	SL3.2.4
EBOV_442_EMLH_KU296375_SLE__2015-01-26	15/01/26	Genbank	UNKNOWN	SL3.2.4
EBOV_443_EMLH_KU296539_SLE_WesternUrban_2015-01-26	15/01/26	Genbank	WAU	SL3.2.4
EBOV_448_EMLH_KU296797_SLE__2015-01-27	15/01/27	Genbank	UNKNOWN	SL3.2.4
NICD6345_SLE_WUR_2015-01-27	15/01/27	NICD	WAU	SL3.2.4
NICD6348_SLE_WUR_2015-01-28	15/01/28	NICD	WAU	SL3.2.4
EBOV_PL2668_KU296294_SLE_PortLoko_2015-01-29	15/01/29	Genbank	Port Loko	SL3.2.4
EBOV_468_EMLH_KU296773_SLE_UNKNOWN_2015-01-29	15/01/29	Genbank	UNKNOWN	SL3.2.4
EBOV_470_EMLH_KU296662_SLE_UNKNOWN_2015-01-29	15/01/29	Genbank	UNKNOWN	SL3.2.4
EBOV_480_EMLH_KU296491_SLE_UNKNOWN_2015-01-29	15/01/29	Genbank	UNKNOWN	SL3.2.4
NICD6403_SLE_WUR_2015-01-29	15/01/29	NICD	WAU	SL3.2.4
NICD6404_SLE_WUR_2015-01-29	15/01/29	NICD	WAU	SL3.2.4
EBOV_489_EMLH_KU296324_SLE_WesternRural_2015-01-30	15/01/30	Genbank	WAR	SL3.2.4
NICD6392-Vero_SLE_WUR_2015-01-30	15/01/30	NICD	WAU	SL3.2.4
NICD6414_SLE_WUR_2014-01-30	15/01/30	NICD	WAU	SL3.2.4
EBOV_KT4609_KU296622_SLE__2015-01-31	15/01/31	Genbank	UNKNOWN	SL3.2.4
EBOV_KT4622_KU296587_SLE_UNKNOWN_2015-02-01	15/02/01	Genbank	UNKNOWN	SL3.2.4
EBOV_511_EMLH_KU296646_SLE_WesternUrban_2015-02-01	15/02/01	Genbank	WAU	SL3.2.4
EBOV_518_EMLH_KU296747_SLE_UNKNOWN_2015-02-02	15/02/02	Genbank	UNKNOWN	SL3.2.4
EBOV_KT4631_KU296607_SLE_WesternUrban_2015-02-02	15/02/02	Genbank	WAU	SL3.2.4
EBOV_554_EMLH_KU296817_SLE_UNKNOWN_2015-02-03	15/02/03	Genbank	UNKNOWN	SL3.2.4
EBOV_KT4677_KU296608_SLE_WesternRural_2015-02-03	15/02/03	Genbank	WAR	SL3.2.4
EBOV_KT4695_KU296549_SLE_WesternRural_2015-02-04	15/02/04	Genbank	WAR	SL3.2.4

EBOV_611_EMLH_KU296619_SLE_UNKNOWN_2015-02-05	15/02/05	Genbank	UNKNOWN	SL3.2.4
EBOV_614_EMLH_KU296597_SLE_WesternRural_2015-02-05	15/02/05	Genbank	WAR	SL3.2.4
EBOV_632_EMLH_KU296693_SLE_WesternUrban_2015-02-06	15/02/06	Genbank	WAU	SL3.2.4
EBOV_634_EMLH_KU296799_SLE_WesternUrban_2015-02-06	15/02/06	Genbank	WAU	SL3.2.4
EBOV_651_EMLH_KU296457_SLE__2015-02-07	15/02/07	Genbank	UNKNOWN	SL3.2.4
EBOV_KT4730_KU296416_SLE_UNKNOWN_2015-02-07	15/02/07	Genbank	UNKNOWN	SL3.2.4
NICD6564_SLE_WUR_2015-02-07	15/02/07	NICD	WAU	SL3.2.4
EBOV_705_EMLH_KU296683_SLE_UNKNOWN_2015-02-09	15/02/09	Genbank	UNKNOWN	SL3.2.4
EBOV_KT4828_KU296745_SLE_UNKNOWN_2015-02-10	15/02/10	Genbank	UNKNOWN	SL3.2.4
EBOV_10402_EMLK_KU296635_SLE_WesternUrban_2015-02-10	15/02/10	Genbank	WAU	SL3.2.4
EBOV_10415_EMLK_KU296741_SLE_WesternUrban_2015-02-10	15/02/10	Genbank	WAU	SL3.2.4
NICD6587_SLE_WUR_2015-02-10	15/02/10	NICD	WAU	SL3.2.4
EBOV_732_EMLH_KU296391_SLE_UNKNOWN_2015-02-11	15/02/11	Genbank	UNKNOWN	SL3.2.4
EBOV_KT4935_KU296467_SLE_WesternRural_2015-02-11	15/02/11	Genbank	WAR	SL3.2.4
EBOV_731_EMLH_KU296765_SLE_WesternUrban_2015-02-11	15/02/11	Genbank	WAU	SL3.2.4
EBOV_741_EMLH_KU296295_SLE_WesternUrban_2015-02-11	15/02/11	Genbank	WAU	SL3.2.4
NICD6601_SLE_WUR_2015-02-11	15/02/11	NICD	WAU	SL3.2.4
NICD6605_SLE_WUR_2015-02-11	15/02/11	NICD	WAU	SL3.2.4
EBOV_762_EMLH_KU296728_SLE_UNKNOWN_2015-02-12	15/02/12	Genbank	UNKNOWN	SL3.2.4
EBOV_10454_EMLK_KU296679_SLE_WesternUrban_2015-02-12	15/02/12	Genbank	WAU	SL3.2.4
EBOV_10455_EMLK_KU296388_SLE_WesternUrban_2015-02-12	15/02/12	Genbank	WAU	SL3.2.4
EBOV_MK2008_KU296526_SLE_WesternUrban_2015-02-12	15/02/12	Genbank	WAU	SL3.2.4
EBOV_793_EMLH_KU296492_SLE_UNKNOWN_2015-02-13	15/02/13	Genbank	UNKNOWN	SL3.2.4
EBOV_14521_EMLK_KU296755_SLE_WesternUrban_2015-02-13	15/02/13	Genbank	WAU	SL3.2.4
NICD6640_SLE_WUR_2015-02-13	15/02/13	NICD	WAU	SL3.2.4
NICD6643_SLE_WUR_2015-02-13	15/02/13	NICD	WAU	SL3.2.4
NICD6644_SLE_WUR_2015-02-13	15/02/13	NICD	WAU	SL3.2.4
EBOV_KT5034_KU296632_SLE__2015-02-14	15/02/14	Genbank	UNKNOWN	SL3.2.4
EBOV_12004_EMLH_KU296302_SLE_WesternRural_2015-02-14	15/02/14	Genbank	WAR	SL3.2.4
NICD6672_SLE_WUR_2015-02-14	15/02/14	NICD	WAU	SL3.2.4
EBOV_KT5073_KU296553_SLE_UNKNOWN_2015-02-16	15/02/16	Genbank	UNKNOWN	SL3.2.4
EBOV_DML12033_KT357813_SLE_WesternUrban_2015-02-19	15/02/19	Genbank	WAU	SL3.2.4
EBOV_Goderich1_KT345616_SLE_WesternUrban_2015-02-19	15/02/19	Genbank	WAU	SL3.2.4
EBOV_KT5214_KU296338_SLE_UNKNOWN_2015-02-20	15/02/20	Genbank	UNKNOWN	SL3.2.4

EBOV_KT5542_KU296557_SLE_UNKNOWN_2015-02-20	15/02/20	Genbank	UNKNOWN	SL3.2.4
EBOV_MK2255_KU296648_SLE_Bombali_2015-02-21	15/02/21	Genbank	Bombali	SL3.2.4
EBOV_12161_EMLH_KU296697_SLE_UNKNOWN_2015-02-21	15/02/21	Genbank	UNKNOWN	SL3.2.4
EBOV_KT5224_KU296383_SLE_UNKNOWN_2015-02-21	15/02/21	Genbank	UNKNOWN	SL3.2.4
EBOV_KT5225_KU296517_SLE_UNKNOWN_2015-02-21	15/02/21	Genbank	UNKNOWN	SL3.2.4
EBOV_KT5233_KU296750_SLE_WesternRural_2015-02-21	15/02/21	Genbank	WAR	SL3.2.4
EBOV_14687_EMLK_KU296443_SLE_WesternUrban_2015-02-21	15/02/21	Genbank	WAU	SL3.2.4
EBOV_DML12051_KT357814_SLE_WesternUrban_2015-02-21	15/02/21	Genbank	WAU	SL3.2.4
EBOV_MK2282_KU296788_SLE_Bombali_2015-02-22	15/02/22	Genbank	Bombali	SL3.2.4
EBOV_MK2283_KU296833_SLE_Bombali_2015-02-22	15/02/22	Genbank	Bombali	SL3.2.4
EBOV_KT5245_KU296411_SLE_UNKNOWN_2015-02-22	15/02/22	Genbank	UNKNOWN	SL3.2.4
EBOV_12178_EMLH_KU296525_SLE_WesternUrban_2015-02-22	15/02/22	Genbank	WAU	SL3.2.4
EBOV_12179_EMLH_KU296531_SLE_WesternUrban_2015-02-22	15/02/22	Genbank	WAU	SL3.2.4
EBOV_12180_EMLH_KU296310_SLE_WesternUrban_2015-02-22	15/02/22	Genbank	WAU	SL3.2.4
EBOV_KT5249_KU296601_SLE_WesternUrban_2015-02-22	15/02/22	Genbank	WAU	SL3.2.4
EBOV_MK2334_KU296366_SLE_Bombali_2015-02-23	15/02/23	Genbank	Bombali	SL3.2.4
EBOV_DML25180_KT357855_SLE_Kono_2015-02-23	15/02/23	Genbank	Kono	SL3.2.4
EBOV_MK2307_KU296536_SLE_UNKNOWN_2015-02-23	15/02/23	Genbank	UNKNOWN	SL3.2.4
EBOV_12181_EMLH_KU296783_SLE_WesternUrban_2015-02-23	15/02/23	Genbank	WAU	SL3.2.4
EBOV_12189_EMLH_KU296369_SLE_WesternUrban_2015-02-23	15/02/23	Genbank	WAU	SL3.2.4
EBOV_12192_EMLH_KU296511_SLE_WesternUrban_2015-02-23	15/02/23	Genbank	WAU	SL3.2.4
EBOV_12193_EMLH_KU296616_SLE_WesternUrban_2015-02-23	15/02/23	Genbank	WAU	SL3.2.4
EBOV_MK2341_KU296494_SLE_Bombali_2015-02-24	15/02/24	Genbank	Bombali	SL3.2.4
EBOV_MK2342_KU296347_SLE_Bombali_2015-02-24	15/02/24	Genbank	Bombali	SL3.2.4
EBOV_MK2343_KU296640_SLE_Bombali_2015-02-24	15/02/24	Genbank	Bombali	SL3.2.4
EBOV_MK2344_KU296586_SLE_Bombali_2015-02-24	15/02/24	Genbank	Bombali	SL3.2.4
EBOV_MK2362_KU296842_SLE_Bombali_2015-02-24	15/02/24	Genbank	Bombali	SL3.2.4
EBOV_MK2364_KU296719_SLE_Bombali_2015-02-24	15/02/24	Genbank	Bombali	SL3.2.4
EBOV_MK2365_KU296363_SLE_Bombali_2015-02-25	15/02/25	Genbank	Bombali	SL3.2.4
EBOV_MK2366_KU296810_SLE_Bombali_2015-02-25	15/02/25	Genbank	Bombali	SL3.2.4
EBOV_MK2367_KU296652_SLE_Bombali_2015-02-25	15/02/25	Genbank	Bombali	SL3.2.4
EBOV_MK2369_KU296832_SLE_Bombali_2015-02-25	15/02/25	Genbank	Bombali	SL3.2.4
EBOV_MK2370_KU296760_SLE_Bombali_2015-02-25	15/02/25	Genbank	Bombali	SL3.2.4
EBOV_MK2371_KU296397_SLE_Bombali_2015-02-25	15/02/25	Genbank	Bombali	SL3.2.4

EBOV_MK2382_KU296686_SLE_Bombali_2015-02-25	15/02/25	Genbank	Bombali	SL3.2.4
EBOV_MK2383_KU296583_SLE_Bombali_2015-02-25	15/02/25	Genbank	Bombali	SL3.2.4
EBOV_MK2395_KU296336_SLE_Bombali_2015-02-25	15/02/25	Genbank	Bombali	SL3.2.4
EBOV_MK2397_KU296444_SLE_Bombali_2015-02-25	15/02/25	Genbank	Bombali	SL3.2.4
EBOV_MK2398_KU296578_SLE_Bombali_2015-02-25	15/02/25	Genbank	Bombali	SL3.2.4
EBOV_MK2405_KU296542_SLE_Bombali_2015-02-25	15/02/25	Genbank	Bombali	SL3.2.4
EBOV_MK2427_KU296759_SLE_Bombali_2015-02-25	15/02/25	Genbank	Bombali	SL3.2.4
EBOV_MK2431_KU296562_SLE_Bombali_2015-02-25	15/02/25	Genbank	Bombali	SL3.2.4
EBOV_PL3984_KU296330_SLE_PortLoko_2015-02-25	15/02/25	Genbank	Port Loko	SL3.2.4
EBOV_KT5382_KU296657_SLE_WesternRural_2015-02-25	15/02/25	Genbank	WAR	SL3.2.4
EBOV_MK2436_KU296626_SLE_Bombali_2015-02-26	15/02/26	Genbank	Bombali	SL3.2.4
EBOV_MK2437_KU296469_SLE_Bombali_2015-02-26	15/02/26	Genbank	Bombali	SL3.2.4
EBOV_MK2447_KU296642_SLE_Bombali_2015-02-26	15/02/26	Genbank	Bombali	SL3.2.4
EBOV_MK2448_KU296818_SLE_Bombali_2015-02-26	15/02/26	Genbank	Bombali	SL3.2.4
EBOV_MK2449_KU296466_SLE_Bombali_2015-02-26	15/02/26	Genbank	Bombali	SL3.2.4
EBOV_PL4059_KU296485_SLE_PortLoko_2015-02-26	15/02/26	Genbank	Port Loko	SL3.2.4
EBOV_DML12116_KT357815_SLE_WesternUrban_2015-02-26	15/02/26	Genbank	WAU	SL3.2.4
EBOV_DML12117_KT357816_SLE_WesternUrban_2015-02-26	15/02/26	Genbank	WAU	SL3.2.4
EBOV_MK2506_KU296360_SLE_Bombali_2015-02-27	15/02/27	Genbank	Bombali	SL3.2.4
EBOV_KT5486_KU296680_SLE_UNKNOWN_2015-02-27	15/02/27	Genbank	UNKNOWN	SL3.2.4
EBOV_12229_EMLH_KU296643_SLE_WesternRural_2015-02-27	15/02/27	Genbank	WAR	SL3.2.4
EBOV_DML12120_KT357817_SLE_WesternUrban_2015-02-27	15/02/27	Genbank	WAU	SL3.2.4
EBOV_PL4105_KU296694_SLE_PortLoko_2015-02-28	15/02/28	Genbank	Port Loko	SL3.2.4
EBOV_12242_EMLH_KU296604_SLE_WesternUrban_2015-02-28	15/02/28	Genbank	WAU	SL3.2.4
EBOV_DML12137_KT357819_SLE_WesternUrban_2015-02-28	15/02/28	Genbank	WAU	SL3.2.4
EBOV_MK2570_KU296346_SLE_Bombali_2015-03-01	15/03/01	Genbank	Bombali	SL3.2.4
EBOV_12257_EMLH_KU296702_SLE_PortLoko_2015-03-01	15/03/01	Genbank	Port Loko	SL3.2.4
EBOV_PL4187_KU296567_SLE_PortLoko_2015-03-01	15/03/01	Genbank	Port Loko	SL3.2.4
EBOV_PL4190_KU296351_SLE_PortLoko_2015-03-01	15/03/01	Genbank	Port Loko	SL3.2.4
EBOV_PL4194_KU296501_SLE_PortLoko_2015-03-02	15/03/02	Genbank	Port Loko	SL3.2.4
EBOV_12275_EMLH_KU296538_SLE_WesternUrban_2015-03-02	15/03/02	Genbank	WAU	SL3.2.4
EBOV_MK2673_KU296749_SLE_Bombali_2015-03-03	15/03/03	Genbank	Bombali	SL3.2.4
EBOV_PL4292_KU296827_SLE_Kambia_2015-03-03	15/03/03	Genbank	Kambia	SL3.2.4
EBOV_MK2717_KU296425_SLE_Bombali_2015-03-04	15/03/04	Genbank	Bombali	SL3.2.4

EBOV_PL4346_KU296641_SLE_Kambia_2015-03-04	15/03/04	Genbank	Kambia	SL3.2.4
EBOV_DML12194_KT357818_SLE_WesternUrban_2015-03-04	15/03/04	Genbank	WAU	SL3.2.4
EBOV_MK2710_KU296703_SLE_Bombali_2015-03-05	15/03/05	Genbank	Bombali	SL3.2.4
EBOV_MK2724_KU296695_SLE_Bombali_2015-03-05	15/03/05	Genbank	Bombali	SL3.2.4
EBOV_DML25344_KT357856_SLE_Kono_2015-03-06	15/03/06	Genbank	Kono	SL3.2.4
NICD7034_SLE_WUR_2015-03-06	15/03/06	NICD	WAU	SL3.2.4
EBOV_MK2788_KU296573_SLE_Bombali_2015-03-07	15/03/07	Genbank	Bombali	SL3.2.4
EBOV_MK2789_KU296613_SLE_Bombali_2015-03-07	15/03/07	Genbank	Bombali	SL3.2.4
EBOV_MK2790_KU296452_SLE_Bombali_2015-03-07	15/03/07	Genbank	Bombali	SL3.2.4
EBOV_KT5786_KU296590_SLE__2015-03-07	15/03/07	Genbank	UNKNOWN	SL3.2.4
EBOV_KT5788_KU296704_SLE__2015-03-07	15/03/07	Genbank	UNKNOWN	SL3.2.4
EBOV_KT5787_KU296711_SLE_WesternUrban_2015-03-07	15/03/07	Genbank	WAU	SL3.2.4
EBOV_KT5789_KU296713_SLE_WesternUrban_2015-03-07	15/03/07	Genbank	WAU	SL3.2.4
NICD7036_SLE_WUR_2015-03-07	15/03/07	NICD	WAU	SL3.2.4
EBOV_MK2825_KU296402_SLE_Bombali_2015-03-08	15/03/08	Genbank	Bombali	SL3.2.4
EBOV_MK2826_KU296405_SLE_Bombali_2015-03-08	15/03/08	Genbank	Bombali	SL3.2.4
EBOV_MK2850_KU296374_SLE_Bombali_2015-03-09	15/03/09	Genbank	Bombali	SL3.2.4
EBOV_DML12239_KT357820_SLE_WesternUrban_2015-03-09	15/03/09	Genbank	WAU	SL3.2.4
EBOV_DML12260_KT357821_SLE_WesternUrban_2015-03-09	15/03/09	Genbank	WAU	SL3.2.4
EBOV_MK2900_KU296787_SLE_Bombali_2015-03-10	15/03/10	Genbank	Bombali	SL3.2.4
EBOV_PL4569_KU296598_SLE_PortLoko_2015-03-10	15/03/10	Genbank	Port Loko	SL3.2.4
EBOV_PL4570_KU296451_SLE_PortLoko_2015-03-10	15/03/10	Genbank	Port Loko	SL3.2.4
EBOV_KT5832_KU296493_SLE_UNKNOWN_2015-03-10	15/03/10	Genbank	UNKNOWN	SL3.2.4
EBOV_KT5858_KU296754_SLE__2015-03-10	15/03/10	Genbank	UNKNOWN	SL3.2.4
EBOV_KT5859_KU296463_SLE_UNKNOWN_2015-03-10	15/03/10	Genbank	UNKNOWN	SL3.2.4
EBOV_DML12268_KT357822_SLE_WesternUrban_2015-03-10	15/03/10	Genbank	WAU	SL3.2.4
EBOV_MK2938_KU296771_SLE_Bombali_2015-03-11	15/03/11	Genbank	Bombali	SL3.2.4
EBOV_MK2983_KU296393_SLE_Bombali_2015-03-12	15/03/12	Genbank	Bombali	SL3.2.4
EBOV_PL4651_KU296841_SLE_PortLoko_2015-03-12	15/03/12	Genbank	Port Loko	SL3.2.4
EBOV_PL4671_KU296782_SLE_PortLoko_2015-03-12	15/03/12	Genbank	Port Loko	SL3.2.4
EBOV_PL4679_KU296623_SLE_UNKNOWN_2015-03-12	15/03/12	Genbank	UNKNOWN	SL3.2.4
EBOV_PL4680_KU296689_SLE_UNKNOWN_2015-03-12	15/03/12	Genbank	UNKNOWN	SL3.2.4
EBOV_Makona-UK3_KR025228_SLE_WesternArea_2015-03-12	15/03/12	Genbank	Western Area	SL3.2.4

EBOV_PL4696_KU296513_SLE_PortLoko_2015-03-13	15/03/13	Genbank	Port Loko	SL3.2.4
EBOV_MK3043_KU296474_SLE_Bombali_2015-03-14	15/03/14	Genbank	Bombali	SL3.2.4
EBOV_KT5974_KU296475_SLE_UNKNOWN_2015-03-16	15/03/16	Genbank	UNKNOWN	SL3.2.4
EBOV_MK3183_KU296800_SLE_Bombali_2015-03-17	15/03/17	Genbank	Bombali	SL3.2.4
EBOV_PL5001_KU296514_SLE_PortLoko_2015-03-19	15/03/19	Genbank	Port Loko	SL3.2.4
EBOV_PL5019_KU296627_SLE_PortLoko_2015-03-19	15/03/19	Genbank	Port Loko	SL3.2.4
EBOV_PL5099_KU296682_SLE_PortLoko_2015-03-19	15/03/19	Genbank	Port Loko	SL3.2.4
EBOV_12578_EMLH_KU296307_SLE_WesternRural_2015-03-20	15/03/20	Genbank	WAR	SL3.2.4
EBOV_12579_EMLH_KU296548_SLE_WesternRural_2015-03-20	15/03/20	Genbank	WAR	SL3.2.4
EBOV_12580_EMLH_KU296574_SLE_WesternRural_2015-03-20	15/03/20	Genbank	WAR	SL3.2.4
EBOV_12600_EMLH_KU296449_SLE_WesternUrban_2015-03-21	15/03/21	Genbank	WAU	SL3.2.4
EBOV_PL5179_KU296638_SLE_PortLoko_2015-03-22	15/03/22	Genbank	Port Loko	SL3.2.4
EBOV_PL5202_KU296396_SLE_PortLoko_2015-03-23	15/03/23	Genbank	Port Loko	SL3.2.4
EBOV_PL5228_KU296535_SLE_PortLoko_2015-03-23	15/03/23	Genbank	Port Loko	SL3.2.4
EBOV_12628_EMLH_KU296692_SLE_WesternRural_2015-03-23	15/03/23	Genbank	WAR	SL3.2.4
EBOV_12634_EMLH_KU296653_SLE_WesternUrban_2015-03-24	15/03/24	Genbank	WAU	SL3.2.4
EBOV_MK3462_KU296565_SLE_Bombali_2015-03-26	15/03/26	Genbank	Bombali	SL3.2.4
EBOV_MK3479_KU296483_SLE_Bombali_2015-03-26	15/03/26	Genbank	Bombali	SL3.2.4
EBOV_12666_EMLH_KU296803_SLE_WesternRural_2015-03-26	15/03/26	Genbank	WAR	SL3.2.4
EBOV_DML12485_KT357824_SLE_WesternUrban_2015-03-31	15/03/31	Genbank	WAU	SL3.2.4
EBOV_12757_EMLH_KU296468_SLE_WesternUrban_2015-04-03	15/04/03	Genbank	WAU	SL3.2.4
EBOV_12767_EMLH_KU296768_SLE_WesternUrban_2015-04-04	15/04/04	Genbank	WAU	SL3.2.4
EBOV_PL5868_KU296550_SLE_Kambia_2015-04-06	15/04/06	Genbank	Kambia	SL3.2.4
EBOV_12781_EMLH_KU296520_SLE_WesternUrban_2015-04-06	15/04/06	Genbank	WAU	SL3.2.4
EBOV_PL6086_KU296651_SLE_Kambia_2015-04-12	15/04/12	Genbank	Kambia	SL3.2.4
EBOV_12851_EMLH_KU296671_SLE_WesternUrban_2015-04-15	15/04/15	Genbank	WAU	SL3.2.4
EBOV_12854_EMLH_KU296729_SLE_WesternUrban_2015-04-15	15/04/15	Genbank	WAU	SL3.2.4
EBOV_12855_EMLH_KU296723_SLE_WesternUrban_2015-04-15	15/04/15	Genbank	WAU	SL3.2.4
EBOV_12872_EMLH_KU296560_SLE_WesternUrban_2015-04-18	15/04/18	Genbank	WAU	SL3.2.4
EBOV_14782_EMLK_KU296639_SLE_Kambia_2015-04-23	15/04/23	Genbank	Kambia	SL3.2.4
EBOV_14830_EMLK_KU296345_SLE_Kambia_2015-04-27	15/04/27	Genbank	Kambia	SL3.2.4
EBOV_12916_EMLH_KU296318_SLE_WesternUrban_2015-04-29	15/04/29	Genbank	WAU	SL3.2.4
EBOV_14888_EMLK_KU296423_SLE_Kambia_2015-05-03	15/05/03	Genbank	Kambia	SL3.2.4
EBOV_12934_EMLH_KU296790_SLE_WesternUrban_2015-05-04	15/05/04	Genbank	WAU	SL3.2.4

EBOV_12936_EMLH_KU296561_SLE_WesternUrban_2015-05-04	15/05/04	Genbank	WAU	SL3.2.4
EBOV_13031_EMLH_KU296665_SLE_WesternUrban_2015-05-18	15/05/18	Genbank	WAU	SL3.2.4
EBOV_PL7483_KU296673_SLE_UNKNOWN_2015-05-31	15/05/31	Genbank	UNKNOWN	SL3.2.4
EBOV_13204_EMLH_KU296544_SLE_WesternUrban_2015-06-16	15/06/16	Genbank	WAU	SL3.2.4
EBOV_13247_EMLH_KU296830_SLE_UNKNOWN_2015-06-19	15/06/19	Genbank	UNKNOWN	SL3.2.4
EBOV_13268_EMLH_KU296603_SLE__2015-06-23	15/06/23	Genbank	UNKNOWN	SL3.2.4
EBOV_13275_EMLH_KU296430_SLE_WesternUrban_2015-06-24	15/06/24	Genbank	WAU	SL3.2.4
EBOV_PL8486_KU296446_SLE__2015-06-25	15/06/25	Genbank	UNKNOWN	SL3.2.4
EBOV_DML14077_KT357860_SLE_WesternUrban_2015-06-30	15/06/30	Genbank	WAU	SL3.2.4
EBOV_DML14163_KT357858_SLE_WesternUrban_2015-07-03	15/07/03	Genbank	WAU	SL3.2.4
EBOV_18538_EMLH_KU296727_SLE__2015-07-04	15/07/04	Genbank	UNKNOWN	SL3.2.4
EBOV_18596_EMLH_KU296715_SLE_WesternUrban_2015-07-05	15/07/05	Genbank	WAU	SL3.2.4
EBOV_18636_EMLH_KU296460_SLE_UNKNOWN_2015-07-08	15/07/08	Genbank	UNKNOWN	SL3.2.4
EBOV_18638R_EMLH_KU296348_SLE_WesternUrban_2015-07-08	15/07/08	Genbank	WAU	SL3.2.4
EBOV_18642R_EMLH_KU296357_SLE_WesternUrban_2015-07-08	15/07/08	Genbank	WAU	SL3.2.4
EBOV_18647_EMLH_KU296566_SLE_WesternUrban_2015-07-09	15/07/09	Genbank	WAU	SL3.2.4
EBOV_18648_EMLH_KU296701_SLE_WesternUrban_2015-07-09	15/07/09	Genbank	WAU	SL3.2.4
EBOV_18649_EMLH_KU296456_SLE_WesternUrban_2015-07-10	15/07/10	Genbank	WAU	SL3.2.4
EBOV_18650_EMLH_KU296370_SLE_WesternUrban_2015-07-10	15/07/10	Genbank	WAU	SL3.2.4
EBOV_18659R_EMLH_KU296585_SLE_WesternUrban_2015-07-10	15/07/10	Genbank	WAU	SL3.2.4
EBOV_18660_EMLH_KU296838_SLE_WesternUrban_2015-07-10	15/07/10	Genbank	WAU	SL3.2.4
EBOV_DML14366_KT357859_SLE_WesternUrban_2015-07-11	15/07/11	Genbank	WAU	SL3.2.4
EBOV_18687R_EMLH_KU296650_SLE_WesternUrban_2015-07-12	15/07/12	Genbank	WAU	SL3.2.4
EBOV_18706_EMLH_KU296599_SLE_UNKNOWN_2015-07-14	15/07/14	Genbank	UNKNOWN	SL3.2.4
EBOV_MK9396_KU296321_SLE_WesternUrban_2015-07-18	15/07/18	Genbank	WAU	SL3.2.4
EBOV_MK8878_KU296684_SLE_Tonkolili_2015-07-23	15/07/23	Genbank	Tonkolili	SL3.2.4
EBOV_MK10128_KU296502_SLE_Tonkolili_2015-07-31	15/07/31	Genbank	Tonkolili	SL3.2.4
EBOV_MK10173_KU296313_SLE_Tonkolili_2015-08-01	15/08/01	Genbank	Tonkolili	SL3.2.4
EBOV_15070R_DML__SLE_WesternUrban_2015-08-06	15/08/06	Genbank	WAU	SL3.2.4
EBOV_G4868_KR105275_SLE_UNKNOWN_2014-08-10	14/08/10	Genbank	UNKNOWN	SL3.2.5
EBOV_G5019_KR105294_SLE_Kenema_2014-08-16	14/08/16	Genbank	Kenema	SL3.2.5
EBOV_20140024_KR653252_SLE_PortLoko_2014-08-20	14/08/20	Genbank	Port Loko	SL3.2.5
EBOV_G5304_KR105304_SLE_UNKNOWN_2014-08-28	14/08/28	Genbank	UNKNOWN	SL3.2.5
NICD0086_SLE_BOM_2014-08-29	14/08/29	NICD	Bombali	SL3.2.5

NICD0129_SLE_BOM_2014-08-31	14/08/31	NICD	Bombali	SL3.2.5
NICD0188_SLE_BOM_2014-09-03	14/09/03	NICD	Bombali	SL3.2.5
NICD0190_SLE_BOM_2014-09-03	14/09/03	NICD	Bombali	SL3.2.5
NICD0193_SLE_BOM_2014-09-03	14/09/03	NICD	Bombali	SL3.2.5
NICD0256_SLE_BOM_2014-09-04	14/09/04	NICD	Bombali	SL3.2.5
NICD0234_SLE_PL_2014-09-04	14/09/04	NICD	Port Loko	SL3.2.5
NICD0235_SLE_PL_2014-09-04	14/09/04	NICD	Port Loko	SL3.2.5
NICD0257_SLE_BOM_2014-09-05	14/09/05	NICD	Bombali	SL3.2.5
NICD0264_SLE_BOM_2014-09-05	14/09/05	NICD	Bombali	SL3.2.5
NICD0299_SLE_PL_2014-09-05	14/09/05	NICD	Port Loko	SL3.2.5
NICD0369_SLE_BOM_2014-09-07	14/09/07	NICD	Bombali	SL3.2.5
NICD0650_SLE_BOM_2014-09-15	14/09/15	NICD	Bombali	SL3.2.5
NICD0637_SLE_TONK_2014-09-16	14/09/16	NICD	Tonkolili	SL3.2.5
NICD0782_SLE_BOM_2014-09-17	14/09/17	NICD	Bombali	SL3.2.5
NICD0919_SLE_TONK_2014-09-19	14/09/19	NICD	Tonkolili	SL3.2.5
NICD1079_SLE_BOM_2014-09-20	14/09/20	NICD	Bombali	SL3.2.5
NICD1089-Vero_SLE_BOM_2014-09-20	14/09/20	NICD	Bombali	SL3.2.5
NICD1201_SLE_TONK_2014-09-20	14/09/20	NICD	Tonkolili	SL3.2.5
NICD1100_SLE_BOM_2014-09-21	14/09/21	NICD	Bombali	SL3.2.5
NICD1016_SLE_PL_2014-09-21	14/09/21	NICD	Port Loko	SL3.2.5
NICD1147_SLE_PL_2014-09-22	14/09/22	NICD	Port Loko	SL3.2.5
NICD1148_SLE_PL_2014-09-22	14/09/22	NICD	Port Loko	SL3.2.5
NICD1156_SLE_PL_2014-09-22	14/09/22	NICD	Port Loko	SL3.2.5
NICD1167_SLE_PL_2014-09-22	14/09/22	NICD	Port Loko	SL3.2.5
NICD1317_SLE_BOM_2014-09-24	14/09/24	NICD	Bombali	SL3.2.5
EBOV_20141271_KR653261_SLE_PortLoko_2014-09-24	14/09/24	Genbank	Port Loko	SL3.2.5
EBOV_G5983_KR105333_SLE_UNKNOWN_2014-09-25	14/09/25	Genbank	UNKNOWN	SL3.2.5
EBOV_G5996_KR105337_SLE_UNKNOWN_2014-09-25	14/09/25	Genbank	UNKNOWN	SL3.2.5
EBOV_G5997_KR105338_SLE_UNKNOWN_2014-09-25	14/09/25	Genbank	UNKNOWN	SL3.2.5
EBOV_G5998_KR105339_SLE_UNKNOWN_2014-09-25	14/09/25	Genbank	UNKNOWN	SL3.2.5
NICD1422_SLE_PL_2014-09-26	14/09/26	NICD	Port Loko	SL3.2.5
NICD1430_SLE_PL_2014-09-26	14/09/26	NICD	Port Loko	SL3.2.5
EBOV_G6089_KR105345_SLE_Tonkolili_2014-09-27	14/09/27	Genbank	Tonkolili	SL3.2.5
EBOV_G6091_KR105346_SLE_Tonkolili_2014-09-27	14/09/27	Genbank	Tonkolili	SL3.2.5

EBOV_G6095_KR105347_SLE_Tonkolili_2014-09-27	14/09/27	Genbank	Tonkolili	SL3.2.5
EBOV_20141429_KR653260_SLE_Kono_2014-09-28	14/09/28	Genbank	Kono	SL3.2.5
EBOV_J0009_KP759734_SLE_PortLoko_2014-09-28	14/09/28	Genbank	Port Loko	SL3.2.5
EBOV_J0010_KP759639_SLE_PortLoko_2014-09-28	14/09/28	Genbank	Port Loko	SL3.2.5
NICD1514_SLE_PL_2014-09-28	14/09/28	NICD	Port Loko	SL3.2.5
EBOV_J0034_KP759608_SLE_PortLoko_2014-09-30	14/09/30	Genbank	Port Loko	SL3.2.5
NICD1662-Vero_SLE_BOM_2014-10-01	14/10/01	NICD	Bombali	SL3.2.5
EBOV_J0035_KP759609_SLE_PortLoko_2014-10-01	14/10/01	Genbank	Port Loko	SL3.2.5
EBOV_J0023_KP759652_SLE_WesternRural_2014-10-01	14/10/01	Genbank	WAR	SL3.2.5
EBOV_J0027_KP759678_SLE_Bombali_2014-10-02	14/10/02	Genbank	Bombali	SL3.2.5
EBOV_J0025_KP759666_SLE_WesternRural_2014-10-02	14/10/02	Genbank	WAR	SL3.2.5
EBOV_J0031_KP759607_SLE_PortLoko_2014-10-03	14/10/03	Genbank	Port Loko	SL3.2.5
EBOV_J0033_KP759692_SLE_PortLoko_2014-10-03	14/10/03	Genbank	Port Loko	SL3.2.5
EBOV_J0036_KP759694_SLE_PortLoko_2014-10-03	14/10/03	Genbank	Port Loko	SL3.2.5
EBOV_J0037_KP759615_SLE_PortLoko_2014-10-03	14/10/03	Genbank	Port Loko	SL3.2.5
EBOV_J0057_KP759635_SLE_PortLoko_2014-10-05	14/10/05	Genbank	Port Loko	SL3.2.5
EBOV_J0055_KP759723_SLE_PortLoko_2014-10-06	14/10/06	Genbank	Port Loko	SL3.2.5
EBOV_J0046_KP759715_SLE_WesternRural_2014-10-07	14/10/07	Genbank	WAR	SL3.2.5
EBOV_J0056_KP759634_SLE_PortLoko_2014-10-08	14/10/08	Genbank	Port Loko	SL3.2.5
EBOV_J0058_KP759724_SLE_PortLoko_2014-10-08	14/10/08	Genbank	Port Loko	SL3.2.5
EBOV_J0067_KP759731_SLE_PortLoko_2014-10-09	14/10/09	Genbank	Port Loko	SL3.2.5
EBOV_J0070_KP759735_SLE_PortLoko_2014-10-09	14/10/09	Genbank	Port Loko	SL3.2.5
EBOV_J0071_KP759736_SLE_PortLoko_2014-10-09	14/10/09	Genbank	Port Loko	SL3.2.5
EBOV_J0072_KP759737_SLE_PortLoko_2014-10-09	14/10/09	Genbank	Port Loko	SL3.2.5
EBOV_J0062_KP759727_SLE_PortLoko_2014-10-10	14/10/10	Genbank	Port Loko	SL3.2.5
EBOV_J0063_KP759638_SLE_PortLoko_2014-10-10	14/10/10	Genbank	Port Loko	SL3.2.5
NICD2219_SLE_WUR_2014-10-14	14/10/14	NICD	WAU	SL3.2.5
EBOV_J0084_KP759753_SLE_PortLoko_2014-10-16	14/10/16	Genbank	Port Loko	SL3.2.5
EBOV_J0082_KP759751_SLE_WesternRural_2014-10-16	14/10/16	Genbank	WAR	SL3.2.5
NICD2308_SLE_WR_2014-10-16	14/10/16	NICD	WAR	SL3.2.5
NICD2310_SLE_WR_2014-10-16	14/10/16	NICD	WAR	SL3.2.5
EBOV_J0076_KP759744_SLE_WesternRural_2014-10-17	14/10/17	Genbank	WAR	SL3.2.5
EBOV_J0083_KP759752_SLE_PortLoko_2014-10-18	14/10/18	Genbank	Port Loko	SL3.2.5
EBOV_20142551_KR653226_SLE_Koinadugu_2014-10-23	14/10/23	Genbank	Koinadugu	SL3.2.5

NICD2494_SLE_WR_2014-10-23	14/10/23	NICD	WAR	SL3.2.5
EBOV_20142895_KR653268_SLE_Koinadugu_2014-10-24	14/10/24	Genbank	Koinadugu	SL3.2.5
NICD2589_SLE_WR_2014-10-24	14/10/24	NICD	WAR	SL3.2.5
EBOV_20143018_KR653272_SLE_WesternUrban_2014-10-26	14/10/26	Genbank	WAU	SL3.2.5
EBOV_J0095_KP759653_SLE_WesternUrban_2014-10-27	14/10/27	Genbank	WAU	SL3.2.5
EBOV_J0097_KP759762_SLE_WesternRural_2014-10-28	14/10/28	Genbank	WAR	SL3.2.5
EBOV_J0122_KP759671_SLE_WesternUrban_2014-10-29	14/10/29	Genbank	WAU	SL3.2.5
EBOV_20143360_KR653258_SLE_Kenema_2014-10-30	14/10/30	Genbank	Kenema	SL3.2.5
EBOV_J0131_KP759680_SLE_PortLoko_2014-10-30	14/10/30	Genbank	Port Loko	SL3.2.5
EBOV_J0120_KP759599_SLE_WesternRural_2014-10-30	14/10/30	Genbank	WAR	SL3.2.5
EBOV_J0111_KP759595_SLE_WesternUrban_2014-10-30	14/10/30	Genbank	WAU	SL3.2.5
EBOV_J0119_KP759667_SLE_WesternUrban_2014-10-30	14/10/30	Genbank	WAU	SL3.2.5
EBOV_J0127_KP759676_SLE_WesternUrban_2014-10-30	14/10/30	Genbank	WAU	SL3.2.5
EBOV_J0139_KP759687_SLE_WesternUrban_2014-10-30	14/10/30	Genbank	WAU	SL3.2.5
EBOV_20143415_KR653248_SLE_WesternRural_2014-10-31	14/10/31	Genbank	WAR	SL3.2.5
EBOV_J0125_KP759674_SLE_WesternRural_2014-10-31	14/10/31	Genbank	WAR	SL3.2.5
EBOV_J0126_KP759675_SLE_WesternRural_2014-10-31	14/10/31	Genbank	WAR	SL3.2.5
EBOV_J0109_KP759594_SLE_WesternUrban_2014-10-31	14/10/31	Genbank	WAU	SL3.2.5
EBOV_J0110_KP759660_SLE_WesternUrban_2014-10-31	14/10/31	Genbank	WAU	SL3.2.5
EBOV_J0128_KP759677_SLE_WesternUrban_2014-10-31	14/10/31	Genbank	WAU	SL3.2.5
EBOV_20143550_KR653228_SLE_Bombali_2014-11-01	14/11/01	Genbank	Bombali	SL3.2.5
EBOV_J0138_KP759686_SLE_WesternUrban_2014-11-01	14/11/01	Genbank	WAU	SL3.2.5
EBOV_J0130_KP759679_SLE_PortLoko_2014-11-02	14/11/02	Genbank	Port Loko	SL3.2.5
EBOV_20143648_KR653253_SLE_Koinadugu_2014-11-03	14/11/03	Genbank	Koinadugu	SL3.2.5
EBOV_20143659_KR653256_SLE_Koinadugu_2014-11-03	14/11/03	Genbank	Koinadugu	SL3.2.5
EBOV_20143918_KR653240_SLE_WesternRural_2014-11-06	14/11/06	Genbank	WAR	SL3.2.5
EBOV_J0145_KP759693_SLE_WesternRural_2014-11-06	14/11/06	Genbank	WAR	SL3.2.5
EBOV_20143938_KR653264_SLE_Koinadugu_2014-11-07	14/11/07	Genbank	Koinadugu	SL3.2.5
EBOV_J0155_KP759697_SLE_WesternArea_2014-11-07	14/11/07	Genbank	Western Area	SL3.2.5
EBOV_J0158_KP759700_SLE_WesternRural_2014-11-08	14/11/08	Genbank	WAR	SL3.2.5
EBOV_J0161_KP759701_SLE_WesternUrban_2014-11-08	14/11/08	Genbank	WAU	SL3.2.5
EBOV_J0166_KP759704_SLE_WesternUrban_2014-11-08	14/11/08	Genbank	WAU	SL3.2.5
EBOV_J0175_KP759709_SLE_WesternArea_2014-11-11	14/11/11	Genbank	Western	SL3.2.5

			Area	
EBOV_20144819_KR653299_SLE_Moyamba_2014-11-15	14/11/15	Genbank	Moyamba	SL3.2.5
EBOV_20144820_KR653243_SLE_Moyamba_2014-11-15	14/11/15	Genbank	Moyamba	SL3.2.5
NICD3304-swab_SLE_WUR_2014-11-15	14/11/15	NICD	WAU	SL3.2.5
NICD3341_SLE_WUR_2014-11-16	14/11/16	NICD	WAU	SL3.2.5
NICD3420_SLE_WUR_2014-11-17	14/11/17	NICD	WAU	SL3.2.5
EBOV_Makona-Italy-INMI1_KP701371_SLE_WesternArea_2014-11-25	14/11/25	Genbank	Western Area	SL3.2.5
NICD4030-Vero_SLE_WR_2014-11-29	14/11/29	NICD	WAR	SL3.2.5
NICD4072-swab_SLE_WUR_2014-11-30	14/11/30	NICD	WAU	SL3.2.5
NICD4113_SLE_WUR_2014-12-01	14/12/01	NICD	WAU	SL3.2.5
NICD4329_SLE_WUR_2014-12-05	14/12/05	NICD	WAU	SL3.2.5
NICD4356_SLE_WUR_2014-12-06	14/12/06	NICD	WAU	SL3.2.5
EBOV_MK0020_KU296551_SLE_Bombali_2014-12-08	14/12/08	Genbank	Bombali	SL3.2.5
NICD5291_SLE_WUR_2014-12-25	14/12/25	NICD	WAU	SL3.2.5
NICD5293_SLE_WUR_2014-12-25	14/12/25	NICD	WAU	SL3.2.5
EBOV_20146578_KR653281_SLE_Koinadugu_2014-12-26	14/12/26	Genbank	Koinadugu	SL3.2.5
NICD5311_SLE_WUR_2014-12-26	14/12/26	NICD	WAU	SL3.2.5
EBOV_MK0592_KU296814_SLE_Bombali_2014-12-28	14/12/28	Genbank	Bombali	SL3.2.5
EBOV_MK0574_KU296332_SLE_UNKNOWN_2014-12-28	14/12/28	Genbank	UNKNOWN	SL3.2.5
NICD5370_SLE_WR_2014-12-28	14/12/28	NICD	WAR	SL3.2.5
NICD5421_SLE_WUR_2014-12-30	14/12/30	NICD	WAU	SL3.2.5
EBOV_MK0704_KU296663_SLE_Bombali_2014-12-31	14/12/31	Genbank	Bombali	SL3.2.5
EBOV_MK0725_KU296629_SLE_Bombali_2015-01-01	15/01/01	Genbank	Bombali	SL3.2.5
EBOV_77585_EMLK_KU296734_SLE_WesternUrban_2015-01-04	15/01/04	Genbank	WAU	SL3.2.5
EBOV_MK0879_KU296410_SLE_PortLoko_2015-01-05	15/01/05	Genbank	Port Loko	SL3.2.5
EBOV_MK0864_KU296334_SLE_UNKNOWN_2015-01-05	15/01/05	Genbank	UNKNOWN	SL3.2.5
EBOV_MK0894_KU296356_SLE_Bombali_2015-01-06	15/01/06	Genbank	Bombali	SL3.2.5
EBOV_MK1156_KU296605_SLE_Bombali_2015-01-15	15/01/15	Genbank	Bombali	SL3.2.5
EBOV_75711_EMLH_KU296596_SLE_WesternUrban_2015-01-15	15/01/15	Genbank	WAU	SL3.2.5
EBOV_KT5226_KU296297_SLE_UNKNOWN_2015-02-21	15/02/21	Genbank	UNKNOWN	SL3.2.5
EBOV_KT5320_KU296809_SLE_WesternRural_2015-02-24	15/02/24	Genbank	WAR	SL3.2.5
EBOV_KT5388_KU296355_SLE_WesternRural_2015-02-25	15/02/25	Genbank	WAR	SL3.2.5
NICD6823-swab_SLE_WUR_2015-02-26	15/02/26	NICD	WAU	SL3.2.5

EBOV_MK2656_KU296577_SLE_Koinadugu_2015-03-03	15/03/03	Genbank	Koinadugu	SL3.2.5
EBOV_KT5738_KU296394_SLE_UNKNOWN_2015-03-06	15/03/06	Genbank	UNKNOWN	SL3.2.5
EBOV_KT5734_KU296595_SLE_WesternRural_2015-03-06	15/03/06	Genbank	WAR	SL3.2.5
EBOV_MK2918_KU296300_SLE_Koinadugu_2015-03-10	15/03/10	Genbank	Koinadugu	SL3.2.5
EBOV_MK3050_KU296445_SLE_Koinadugu_2015-03-14	15/03/14	Genbank	Koinadugu	SL3.2.5
EBOV_12615_EMLH_KU296521_SLE_WesternRural_2015-03-22	15/03/22	Genbank	WAR	SL3.2.5
EBOV_DML12458_KT357823_SLE_WesternUrban_2015-03-28	15/03/28	Genbank	WAU	SL3.2.5
EBOV_12705_EMLH_KU296490_SLE_WesternUrban_2015-03-29	15/03/29	Genbank	WAU	SL3.2.5
EBOV_MK13013_KU296454_SLE_Bombali_2015-09-12	15/09/12	Genbank	Bombali	SL3.2.5

Table S3. Required members for each sub-lineage

SL3.1.1
020380-EMLK_KU296775_SLE_Kambia_UNKNOWN_UNKNOWN_2015-08-28
020484-EMLK_KU296462_SLE_Kambia_UNKNOWN_UNKNOWN_2015-09-04
14743-EML_KU296298_SLE_Kambia_UNKNOWN_UNKNOWN_2015-04-19
14846-EMLK_KU296831_SLE_Kambia_UNKNOWN_UNKNOWN_2015-04-29
15000-EMLK_KU296504_SLE_Kambia_UNKNOWN_UNKNOWN_2015-05-13
15207R-EMLK_KU296488_SLE_Kambia_UNKNOWN_UNKNOWN_2015-05-29
15274-EMLK_KU296337_SLE_Kambia_UNKNOWN_UNKNOWN_2015-06-04
15314-EMLK_KU296600_SLE_Kambia_UNKNOWN_UNKNOWN_2015-06-06
15323-EMLK_KU296668_SLE_Kambia_UNKNOWN_UNKNOWN_2015-06-07
15327-EMLK_KU296649_SLE_Kambia_UNKNOWN_UNKNOWN_2015-06-07
15338-EMLK_KU296414_SLE_Kambia_UNKNOWN_UNKNOWN_2015-06-08
15349-EMLK_KU296442_SLE_Kambia_UNKNOWN_UNKNOWN_2015-06-09
15360-EMLK_KU296399_SLE_Kambia_UNKNOWN_UNKNOWN_2015-06-08
15373-EMLK_KU296802_SLE_Kambia_UNKNOWN_UNKNOWN_2015-06-10
15421-EMLK_KU296796_SLE_Kambia_UNKNOWN_UNKNOWN_2015-06-13
15438R-EMLK_KU296353_SLE_Kambia_UNKNOWN_UNKNOWN_2015-06-14
15470-EMLK_KU296543_SLE_Kambia_UNKNOWN_UNKNOWN_2015-06-16
15543-EMLK_KU296666_SLE_Kambia_UNKNOWN_UNKNOWN_2015-06-22
15661-EMLK_KU296822_SLE_Kambia_UNKNOWN_UNKNOWN_2015-06-28
15674-EMLK_KU296843_SLE_Kambia_UNKNOWN_UNKNOWN_2015-06-28
15686-EMLK_KU296555_SLE_Kambia_UNKNOWN_UNKNOWN_2015-06-30
19521-EMLK_KU296306_SLE_Kambia_UNKNOWN_UNKNOWN_2015-07-05
19560R-EMLK_KU296580_SLE_Kambia_UNKNOWN_UNKNOWN_2015-07-07
20142407_KR653249_SLE_Tonkolili_UNKNOWN_UNKNOWN_2014-10-16
20142417_KR653254_SLE_Bo_UNKNOWN_UNKNOWN_2014-10-19
20143964_KR653242_SLE_Bo_UNKNOWN_UNKNOWN_2014-11-07
20524-EMLK_KU296424_SLE_Kambia_UNKNOWN_UNKNOWN_2015-09-06
20525-EMLK_KU296487_SLE_Kambia_UNKNOWN_UNKNOWN_2015-09-06
20547-EMLK_KU296455_SLE_Kambia_UNKNOWN_UNKNOWN_2015-09-07
77577-EMLK_KU296533_SLE_WesternUrban_UNKNOWN_UNKNOWN_2015-01-03
77578-EMLK_KU296806_SLE_WesternUrban_UNKNOWN_UNKNOWN_2015-01-03
77588-EMLK_KU296801_SLE_WesternUrban_UNKNOWN_UNKNOWN_2015-01-04
DML24708_KT357845_SLE_Kono_UNKNOWN_UNKNOWN_2015-01-28
J0051_KP759633_SLE_WesternUrban_UNKNOWN_UNKNOWN_2014-10-07
J0053_KP759721_SLE_WesternRural_UNKNOWN_UNKNOWN_2014-10-06
J0054_KP759722_SLE_WesternRural_UNKNOWN_UNKNOWN_2014-10-06
J0092_KP759650_SLE_WesternRural_UNKNOWN_UNKNOWN_2014-10-25
J0099_KP759764_SLE_Kambia_UNKNOWN_UNKNOWN_2014-10-28
J0137_KP759685_SLE_WesternRural_UNKNOWN_UNKNOWN_2014-11-01
KT5022_KU296519_SLE_WesternRural_UNKNOWN_UNKNOWN_2015-02-13
KT7095_KU296690_SLE_Kambia_UNKNOWN_UNKNOWN_2015-07-05
NICD0143_XXXX_SLE_WesternUrban_Lumley_WestIII_2014-08-31

NICD1275_XXXX_SLE_WesternRural_UNKNOWN_UNKNOWN_2014-09-25
NICD2763-Vero_XXXX_SLE_PortLoko_UNKNOWN_UNKNOWN_2014-11-06
NICD3577_XXXX_SLE_WesternRural_UNKNOWN_UNKNOWN_2014-11-20
NICD3926_XXXX_SLE_WesternUrban_FoulahTown_EastII_2014-11-27
NICD5584_XXXX_SLE_WesternUrban_Wilberforce_WestIII_2015-01-01
PL4226_KU296615_SLE_Kambia_UNKNOWN_UNKNOWN_2015-03-02
PL4483_KU296389_SLE_Kambia_UNKNOWN_UNKNOWN_2015-03-08
PL4736_KU296724_SLE_Kambia_UNKNOWN_UNKNOWN_2015-03-13
PL4763_KU296503_SLE_Kambia_UNKNOWN_UNKNOWN_2015-03-14
PL4864_KU296437_SLE_Kambia_UNKNOWN_UNKNOWN_2015-03-16
PL4865_KU296633_SLE_Kambia_UNKNOWN_UNKNOWN_2015-03-16
PL4866_KU296390_SLE_Kambia_UNKNOWN_UNKNOWN_2015-03-16
PL5025_KU296412_SLE_Kambia_UNKNOWN_UNKNOWN_2015-03-20
PL5157_KU296421_SLE_PortLoko_UNKNOWN_UNKNOWN_2015-03-22
PL6049_KU296743_SLE_Kambia_UNKNOWN_UNKNOWN_2015-04-11
PL6656_KU296523_SLE_PortLoko_UNKNOWN_UNKNOWN_2015-05-01
PL7053_KU296757_SLE_PortLoko_UNKNOWN_UNKNOWN_2015-05-15
PL7055_KU296484_SLE_PortLoko_UNKNOWN_UNKNOWN_2015-05-15
PL7136b_KU296732_SLE_PortLoko_UNKNOWN_UNKNOWN_2015-05-18
PL7375_KU296498_SLE_PortLoko_UNKNOWN_UNKNOWN_2015-05-27
PL7376_KU296740_SLE_PortLoko_UNKNOWN_UNKNOWN_2015-05-27
PL7401_KU296819_SLE_PortLoko_UNKNOWN_UNKNOWN_2015-05-27
PL7429_KU296617_SLE_PortLoko_UNKNOWN_UNKNOWN_2015-05-29
PL7451_KU296837_SLE_PortLoko_UNKNOWN_UNKNOWN_2015-05-29
PL7496_KU296846_SLE_PortLoko_UNKNOWN_UNKNOWN_2015-05-31
PL7511_KU296685_SLE_PortLoko_UNKNOWN_UNKNOWN_2015-06-01
PL7551_KU296322_SLE_PortLoko_UNKNOWN_UNKNOWN_2015-06-02
PL7577_KU296385_SLE_PortLoko_UNKNOWN_UNKNOWN_2015-06-03
PL7604_KU296592_SLE_PortLoko_UNKNOWN_UNKNOWN_2015-06-04
PL7605_KU296486_SLE_PortLoko_UNKNOWN_UNKNOWN_2015-06-04
PL7626_KU296753_SLE_PortLoko_UNKNOWN_UNKNOWN_2015-06-04
PL7678_KU296625_SLE_PortLoko_UNKNOWN_UNKNOWN_2015-06-06
PL7685_KU296556_SLE_PortLoko_UNKNOWN_UNKNOWN_2015-06-06
PL7689_KU296712_SLE_PortLoko_UNKNOWN_UNKNOWN_2015-06-06
PL7709_KU296426_SLE_PortLoko_UNKNOWN_UNKNOWN_2015-06-06
PL7792_KU296793_SLE_PortLoko_UNKNOWN_UNKNOWN_2015-06-09
PL7801_KU296327_SLE_PortLoko_UNKNOWN_UNKNOWN_2015-06-09
PL7820_KU296804_SLE_PortLoko_UNKNOWN_UNKNOWN_2015-06-09
PL7834_KU296326_SLE_PortLoko_UNKNOWN_UNKNOWN_2015-06-10
PL7946_KU296667_SLE_PortLoko_UNKNOWN_UNKNOWN_2015-06-12
PL7947_KU296571_SLE_PortLoko_UNKNOWN_UNKNOWN_2015-06-12
PL8128_KU296340_SLE_PortLoko_UNKNOWN_UNKNOWN_2015-06-16
PL8172_KU296428_SLE_PortLoko_UNKNOWN_UNKNOWN_2015-06-18
PL8310_KU296618_SLE_PortLoko_UNKNOWN_UNKNOWN_2015-06-21
PL8429_KU296440_SLE_PortLoko_UNKNOWN_UNKNOWN_2015-06-24

PL8630_KU296325_SLE_PortLoko_UNKNOWN_UNKNOWN_2015-06-29
PL8780_KU296404_SLE_PortLoko_UNKNOWN_UNKNOWN_2015-07-03
PL8896_KU296342_SLE_PortLoko_UNKNOWN_UNKNOWN_2015-07-06
PL9150_KU296522_SLE_PortLoko_UNKNOWN_UNKNOWN_2015-07-12
PL9192_KU296401_SLE_PortLoko_UNKNOWN_UNKNOWN_2015-07-13
PL9199_KU296371_SLE_PortLoko_UNKNOWN_UNKNOWN_2015-07-13
**SL3.1.2**
20140174_KR653294_SLE_WesternUrban_UNKNOWN_UNKNOWN_2014-08-27
20140872_KR653297_SLE_WesternUrban_UNKNOWN_UNKNOWN_2014-09-15
20141352_KR653284_SLE_Kambia_UNKNOWN_UNKNOWN_2014-09-26
20141397_KR653288_SLE_Moyamba_UNKNOWN_UNKNOWN_2014-09-28
20141997_KR653293_SLE_Kono_UNKNOWN_UNKNOWN_2014-10-10
20143317_KR653282_SLE_Bombali_UNKNOWN_UNKNOWN_2014-10-29
20143458_KR653245_SLE_Tonkolili_UNKNOWN_UNKNOWN_2014-11-01
20144521_KR653271_SLE_Kono_UNKNOWN_UNKNOWN_2014-11-12
20146001_KR653237_SLE_Kono_UNKNOWN_UNKNOWN_2014-11-24
361-EMLH_KU296420_SLE_WesternUrban_UNKNOWN_UNKNOWN_2015-01-21
508-EMLH_KU296304_SLE_WesternUrban_UNKNOWN_UNKNOWN_2015-01-31
539-EMLH_KU296823_SLE_WesternUrban_UNKNOWN_UNKNOWN_2015-02-02
75674-EMLH_KU296350_SLE_WesternUrban_UNKNOWN_UNKNOWN_2015-01-13
75731-EMLH_KU296564_SLE_WesternUrban_UNKNOWN_UNKNOWN_2015-01-16
77601-EMLK_KU296826_SLE_WesternUrban_UNKNOWN_UNKNOWN_2015-01-05
DML24502_KT357825_SLE_Kono_UNKNOWN_UNKNOWN_2015-01-13
DML24504_KT357826_SLE_Kono_UNKNOWN_UNKNOWN_2015-01-13
DML24506_KT357827_SLE_Kono_UNKNOWN_UNKNOWN_2015-01-14
DML24511_KT357828_SLE_Kono_UNKNOWN_UNKNOWN_2015-01-14
DML24552_KT357829_SLE_Kono_UNKNOWN_UNKNOWN_2015-01-17
DML24553_KT357830_SLE_Kono_UNKNOWN_UNKNOWN_2015-01-17
DML24573_KT357831_SLE_Kono_UNKNOWN_UNKNOWN_2015-01-18
DML24581_KT357832_SLE_Kono_UNKNOWN_UNKNOWN_2015-01-19
DML24592_KT357833_SLE_Kono_UNKNOWN_UNKNOWN_2015-01-20
DML24601_KT357834_SLE_Kono_UNKNOWN_UNKNOWN_2015-01-20
DML24604_KT357835_SLE_Kono_UNKNOWN_UNKNOWN_2015-01-20
DML24605_KT357836_SLE_Kono_UNKNOWN_UNKNOWN_2015-01-20
DML24606_KT357837_SLE_Kono_UNKNOWN_UNKNOWN_2015-01-20
DML24608_KT357838_SLE_Kono_UNKNOWN_UNKNOWN_2015-01-20
DML24611_KT357839_SLE_Kono_UNKNOWN_UNKNOWN_2015-01-21
DML24620_KT357840_SLE_Kono_UNKNOWN_UNKNOWN_2015-01-21
DML24669_KT357841_SLE_Kono_UNKNOWN_UNKNOWN_2015-01-25
DML24677_KT357842_SLE_Kono_UNKNOWN_UNKNOWN_2015-01-25
DML24683_KT357843_SLE_Kono_UNKNOWN_UNKNOWN_2015-01-26
DML24706_KT357844_SLE_Kono_UNKNOWN_UNKNOWN_2015-01-28
DML24720_KT357846_SLE_Kono_UNKNOWN_UNKNOWN_2015-01-29
DML24758_KT357847_SLE_Kono_UNKNOWN_UNKNOWN_2015-01-30

DML24818_KT357848_SLE_Kono_UNKNOWN_UNKNOWN_2015-02-03
DML24825_KT357849_SLE_Tonkolili_UNKNOWN_UNKNOWN_2015-02-04
DML24853_KT357850_SLE_Kono_UNKNOWN_UNKNOWN_2015-02-06
DML24854_KT357851_SLE_Kono_UNKNOWN_UNKNOWN_2015-02-06
DML25083_KT357852_SLE_Kono_UNKNOWN_UNKNOWN_2015-02-18
DML25103_KT357853_SLE_Kono_UNKNOWN_UNKNOWN_2015-02-19
DML25123_KT357854_SLE_Kenema_UNKNOWN_UNKNOWN_2015-02-18
G5723_KR105319_SLE_Bonthe_UNKNOWN_UNKNOWN_2014-09-15
G6069_KR105344_SLE_Kambia_UNKNOWN_UNKNOWN_2014-09-25
G6103_KR105348_SLE_Moyamba_UNKNOWN_UNKNOWN_2014-09-28
G6104_KR105349_SLE_Moyamba_UNKNOWN_UNKNOWN_2014-09-28
J0001_KP759636_SLE_WesternRural_UNKNOWN_UNKNOWN_2014-09-27
J0002_KP759640_SLE_WesternUrban_UNKNOWN_UNKNOWN_2014-09-27
J0005_KP759628_SLE_WesternRural_UNKNOWN_UNKNOWN_2014-09-26
J0007_KP759631_SLE_WesternRural_UNKNOWN_UNKNOWN_2014-09-27
J0008_KP759718_SLE_WesternUrban_UNKNOWN_UNKNOWN_2014-09-29
J0016_KP759742_SLE_WesternRural_UNKNOWN_UNKNOWN_2014-09-30
J0024_KP759663_SLE_WesternRural_UNKNOWN_UNKNOWN_2014-09-29
J0028_KP759683_SLE_Bombali_UNKNOWN_UNKNOWN_2014-10-02
J0039_KP759620_SLE_PortLoko_UNKNOWN_UNKNOWN_2014-10-03
J0042_KP759711_SLE_WesternRural_UNKNOWN_UNKNOWN_2014-10-04
J0050_KP759719_SLE_WesternUrban_UNKNOWN_UNKNOWN_2014-10-05
J0113_KP759662_SLE_WesternUrban_UNKNOWN_UNKNOWN_2014-10-30
J0121_KP759669_SLE_WesternUrban_UNKNOWN_UNKNOWN_2014-10-29
J0134_KP759682_SLE_WesternRural_UNKNOWN_UNKNOWN_2014-10-31
J0135_KP759602_SLE_WesternRural_UNKNOWN_UNKNOWN_2014-11-03
J0143_KP759604_SLE_Kambia_UNKNOWN_UNKNOWN_2014-11-04
J0144_KP759605_SLE_Kambia_UNKNOWN_UNKNOWN_2014-11-04
J0146_KP759610_SLE_WesternUrban_UNKNOWN_UNKNOWN_2014-11-07
J0147_KP759611_SLE_WesternUrban_UNKNOWN_UNKNOWN_2014-11-07
J0149_KP759695_SLE_WesternUrban_UNKNOWN_UNKNOWN_2014-11-07
J0150_KP759696_SLE_WesternUrban_UNKNOWN_UNKNOWN_2014-11-06
J0157_KP759699_SLE_WesternUrban_UNKNOWN_UNKNOWN_2014-11-06
J0159_KP759619_SLE_WesternRural_UNKNOWN_UNKNOWN_2014-11-05
J0171_KP759707_SLE_WesternRural_UNKNOWN_UNKNOWN_2014-11-10
J0173_KP759627_SLE_WesternRural_UNKNOWN_UNKNOWN_2014-11-11
MK0384_KU296628_SLE_Bombali_UNKNOWN_UNKNOWN_2014-12-22
MK0885_KU296758_SLE_Bombali_UNKNOWN_UNKNOWN_2015-01-06
MK1040_KU296528_SLE_Bombali_UNKNOWN_UNKNOWN_2015-01-11
MK1079_KU296815_SLE_Bombali_UNKNOWN_UNKNOWN_2015-01-11
NICD0022_XXXX_SLE_WesternUrban_Lumley_WestIII_2014-08-26
NICD0031_XXXX_SLE_WesternUrban_Lumley_WestIII_2014-08-26
NICD0034_XXXX_SLE_WesternUrban_Lumley_WestIII_2014-08-26
NICD0393-Vero_XXXX_SLE_WesternUrban_Wellington_EastIII_2014-09-09
NICD0821_XXXX_SLE_WesternRural_UNKNOWN_UNKNOWN_2014-09-20

NICD0983_XXXX_SLE_WesternUrban_Tengbehtown_WestIII_2014-09-22
NICD0986-Vero_XXXX_SLE_WesternUrban_Wilberforce_WestIII_2014-09-22
NICD1113_XXXX_SLE_Bombali_UNKNOWN_UNKNOWN_2014-09-19
NICD1209-Vero_XXXX_SLE_WesternUrban_NewEngland_WestII_2014-09-23
NICD1458_XXXX_SLE_WesternUrban_FoulahTown_EastII_2014-09-29
NICD2031-Vero_XXXX_SLE_WesternUrban_Wilberforce_WestIII_2014-10-11
NICD2032-Vero_XXXX_SLE_WesternUrban_Tengbehtown_WestIII_2014-10-11
NICD2538_XXXX_SLE_WesternUrban_Kortright_EastII_2014-10-22
NICD2541_XXXX_SLE_WesternUrban_NewEngland_WestII_2014-10-22
NICD2748_XXXX_SLE_Kambia_UNKNOWN_UNKNOWN_2014-11-06
NICD3031_XXXX_SLE_PortLoko_UNKNOWN_UNKNOWN_2014-11-11
NICD3087_XXXX_SLE_WesternUrban_CongoTown_WestI_2014-11-13
NICD3089_XXXX_SLE_PortLoko_UNKNOWN_UNKNOWN_2014-11-12
NICD3335_XXXX_SLE_WesternRural_UNKNOWN_UNKNOWN_2014-11-16
NICD3483-Vero_XXXX_SLE_WesternUrban_CalabaTown_EastIII_2014-11-18
NICD3596_XXXX_SLE_WesternUrban_Wellington_EastIII_2014-11-21
NICD3707_XXXX_SLE_WesternUrban_Kissy_EastIII_2014-11-24
NICD3820_XXXX_SLE_WesternUrban_Magazine_CentralI_2014-11-25
NICD3823_XXXX_SLE_WesternRural_UNKNOWN_UNKNOWN_2014-11-25
NICD3925_XXXX_SLE_WesternUrban_Magazine_CentralI_2014-11-27
NICD3928_XXXX_SLE_WesternUrban_AllenTown_EastIII_2014-11-26
NICD3932_XXXX_SLE_WesternUrban_AllenTown_EastIII_2014-11-26
NICD3937_XXXX_SLE_WesternUrban_AllenTown_EastIII_2014-11-26
NICD4025-Vero_XXXX_SLE_WesternUrban_Wellington_EastIII_2014-11-29
NICD4055_XXXX_SLE_WesternUrban_AllenTown_EastIII_2014-11-28
NICD4064-swab_XXXX_SLE_WesternUrban_Freetown_WestII_2014-11-29
NICD4279-Vero_XXXX_SLE_WesternUrban_Kissy_EastIII_2014-12-05
NICD4682_XXXX_SLE_WesternUrban_AllenTown_EastIII_2014-12-13
NICD4830_XXXX_SLE_WesternUrban_AllenTown_EastIII_2014-12-15
NICD5200_XXXX_SLE_WesternUrban_Wellington_EastIII_2014-12-24
NICD5260_XXXX_SLE_WesternUrban_Wellington_EastIII_2014-12-24
NICD5266_XXXX_SLE_WesternUrban_AllenTown_EastIII_2014-12-24
NICD5314_XXXX_SLE_WesternUrban_Wellington_EastIII_2014-12-26
NICD5354_XXXX_SLE_WesternUrban_AllenTown_EastIII_2014-12-27
NICD5360_XXXX_SLE_WesternUrban_AllenTown_EastIII_2014-12-27
NICD5725_XXXX_SLE_WesternUrban_Wellington_EastIII_2015-01-07
NICD6218_XXXX_SLE_WesternUrban_Magazine_CentralI_2015-01-24
NICD6274_XXXX_SLE_WesternUrban_Magazine_CentralI_2015-01-26
PL1387_KU296434_SLE_PortLoko_UNKNOWN_UNKNOWN_2015-01-04
PL4119_KU296645_SLE_PortLoko_UNKNOWN_UNKNOWN_2015-02-28
PL4197_KU296299_SLE_PortLoko_UNKNOWN_UNKNOWN_2015-03-02
PL4203_KU296311_SLE_PortLoko_UNKNOWN_UNKNOWN_2015-03-02
PL4248_KU296658_SLE_PortLoko_UNKNOWN_UNKNOWN_2015-03-03
PL4249_KU296349_SLE_PortLoko_UNKNOWN_UNKNOWN_2015-03-03
PL4283_KU296840_SLE_PortLoko_UNKNOWN_UNKNOWN_2015-03-03

PL4284_KU296829_SLE_PortLoko_UNKNOWN_UNKNOWN_2015-03-03
PL4443_KU296418_SLE_PortLoko_UNKNOWN_UNKNOWN_2015-03-07
PL4785_KU296688_SLE_PortLoko_UNKNOWN_UNKNOWN_2015-03-14
PL4804_KU296766_SLE_PortLoko_UNKNOWN_UNKNOWN_2015-03-15
PL4813_KU296798_SLE_PortLoko_UNKNOWN_UNKNOWN_2015-03-14
PL4844_KU296527_SLE_PortLoko_UNKNOWN_UNKNOWN_2015-03-16
PL4886_KU296461_SLE_PortLoko_UNKNOWN_UNKNOWN_2015-03-16
PL4932_KU296547_SLE_PortLoko_UNKNOWN_UNKNOWN_2015-03-18
PL5133_KU296489_SLE_PortLoko_UNKNOWN_UNKNOWN_2015-03-21
PL5275_KU296439_SLE_PortLoko_UNKNOWN_UNKNOWN_2015-03-24
PL5534_KU296784_SLE_PortLoko_UNKNOWN_UNKNOWN_2015-03-29
PL5674_KU296699_SLE_PortLoko_UNKNOWN_UNKNOWN_2015-03-31
PL5950_KU296559_SLE_PortLoko_UNKNOWN_UNKNOWN_2015-04-08
KT2449_KU296465_SLE_WesternUrban_UNKNOWN_UNKNOWN_2014-12-05
NICD6123_XXXX_SLE_WesternRural_UNKNOWN_UNKNOWN_2015-01-20
NICD6141_XXXX_SLE_WesternRural_UNKNOWN_UNKNOWN_2015-01-21
NICD6365_XXXX_SLE_WesternRural_UNKNOWN_UNKNOWN_2015-01-29
**SL3.2.1**
20140134_KR653227_SLE_Bombali_UNKNOWN_UNKNOWN_2014-08-26
20141232_KR653266_SLE_Tonkolili_UNKNOWN_UNKNOWN_2014-09-25
20141497_KR653273_SLE_Bombali_UNKNOWN_UNKNOWN_2014-10-01
20141960_KR653283_SLE_Tonkolili_UNKNOWN_UNKNOWN_2014-10-09
20142127_KR653234_SLE_Bombali_UNKNOWN_UNKNOWN_2014-10-12
20142260_KR653262_SLE_Bombali_UNKNOWN_UNKNOWN_2014-10-14
20142843_KR653301_SLE_Bombali_UNKNOWN_UNKNOWN_2014-10-23
20142856_KR653302_SLE_Tonkolili_UNKNOWN_UNKNOWN_2014-10-24
20143107_KR653247_SLE_Bombali_UNKNOWN_UNKNOWN_2014-10-25
20144192_KR653238_SLE_Bombali_UNKNOWN_UNKNOWN_2014-11-08
20144837_KR653259_SLE_Bombali_UNKNOWN_UNKNOWN_2014-11-14
20145835_KR653295_SLE_Tonkolili_UNKNOWN_UNKNOWN_2014-11-25
20146553_KR653224_SLE_Koinadugu_UNKNOWN_UNKNOWN_2014-12-22
466-EMLH_KU296433_SLE_WesternUrban_UNKNOWN_UNKNOWN_2015-01-28
J0015_KP759741_SLE_Bombali_UNKNOWN_UNKNOWN_2014-09-26
J0017_KP759747_SLE_Bombali_UNKNOWN_UNKNOWN_2014-09-26
J0019_KP759754_SLE_Bombali_UNKNOWN_UNKNOWN_2014-09-25
J0020_KP759755_SLE_Bombali_UNKNOWN_UNKNOWN_2014-09-25
J0022_KP759757_SLE_Bombali_UNKNOWN_UNKNOWN_2014-09-25
J0096_KP759654_SLE_WesternUrban_UNKNOWN_UNKNOWN_2014-10-27
J0123_KP759672_SLE_WesternRural_UNKNOWN_UNKNOWN_2014-11-01
J0152_KP759614_SLE_WesternRural_UNKNOWN_UNKNOWN_2014-11-07
J0160_KP759621_SLE_WesternRural_UNKNOWN_UNKNOWN_2014-11-07
MK0024_KU296705_SLE_Bombali_UNKNOWN_UNKNOWN_2014-12-08
MK0045_KU296776_SLE_Bombali_UNKNOWN_UNKNOWN_2014-12-09
MK0085_KU296738_SLE_Bombali_UNKNOWN_UNKNOWN_2014-12-10

MK0364_KU296453_SLE_Bombali_UNKNOWN_UNKNOWN_2014-12-21
MK0365_KU296406_SLE_Bombali_UNKNOWN_UNKNOWN_2014-12-21
MK0366_KU296320_SLE_Bombali_UNKNOWN_UNKNOWN_2014-12-21
MK0367_KU296770_SLE_Bombali_UNKNOWN_UNKNOWN_2014-12-21
MK0368_KU296718_SLE_Bombali_UNKNOWN_UNKNOWN_2014-12-21
MK0369_KU296413_SLE_Bombali_UNKNOWN_UNKNOWN_2014-12-21
MK0375_KU296606_SLE_Bombali_UNKNOWN_UNKNOWN_2014-12-22
MK0379_KU296480_SLE_Bombali_UNKNOWN_UNKNOWN_2014-12-22
MK0388_KU296630_SLE_Bombali_UNKNOWN_UNKNOWN_2014-12-22
MK0390_KU296398_SLE_Bombali_UNKNOWN_UNKNOWN_2014-12-22
MK0392_KU296691_SLE_Bombali_UNKNOWN_UNKNOWN_2014-12-22
MK0425_KU296379_SLE_Bombali_UNKNOWN_UNKNOWN_2014-12-23
MK0431_KU296621_SLE_Bombali_UNKNOWN_UNKNOWN_2014-12-23
MK0433_KU296845_SLE_Bombali_UNKNOWN_UNKNOWN_2014-12-23
MK0434_KU296554_SLE_Bombali_UNKNOWN_UNKNOWN_2014-12-23
MK0491_KU296510_SLE_Bombali_UNKNOWN_UNKNOWN_2014-12-23
MK0531_KU296448_SLE_Bombali_UNKNOWN_UNKNOWN_2014-12-26
MK0549_KU296825_SLE_Bombali_UNKNOWN_UNKNOWN_2014-12-27
MK0584_KU296339_SLE_Bombali_UNKNOWN_UNKNOWN_2014-12-28
MK0709_KU296373_SLE_Tonkolili_UNKNOWN_UNKNOWN_2014-12-31
MK0869_KU296534_SLE_Bombali_UNKNOWN_UNKNOWN_2015-01-05
MK0966_KU296507_SLE_Bombali_UNKNOWN_UNKNOWN_2015-01-08
MK1000_KU296384_SLE_Bombali_UNKNOWN_UNKNOWN_2015-01-10
MK1014_KU296316_SLE_Bombali_UNKNOWN_UNKNOWN_2015-01-10
MK1036_KU296834_SLE_Bombali_UNKNOWN_UNKNOWN_2015-01-11
MK1197_KU296301_SLE_Bombali_UNKNOWN_UNKNOWN_2015-01-17
MK1202_KU296581_SLE_Bombali_UNKNOWN_UNKNOWN_2015-01-17
MK1320_KU296354_SLE_Bombali_UNKNOWN_UNKNOWN_2015-01-20
MK4165_KU296575_SLE_Koinadugu_UNKNOWN_UNKNOWN_2015-04-16
NICD0689_XXXX_SLE_Tonkolili_UNKNOWN_UNKNOWN_2014-09-17
NICD0787_XXXX_SLE_Bombali_UNKNOWN_UNKNOWN_2014-09-17
NICD0905_XXXX_SLE_Tonkolili_UNKNOWN_UNKNOWN_2014-09-20
NICD0908_XXXX_SLE_Tonkolili_UNKNOWN_UNKNOWN_2014-09-20
NICD0911_XXXX_SLE_Tonkolili_UNKNOWN_UNKNOWN_2014-09-20
NICD0964_XXXX_SLE_Tonkolili_UNKNOWN_UNKNOWN_2014-09-21
NICD1081_XXXX_SLE_Bombali_UNKNOWN_UNKNOWN_2014-09-20
NICD1092_XXXX_SLE_Bombali_UNKNOWN_UNKNOWN_2014-09-20
NICD1105_XXXX_SLE_Bombali_UNKNOWN_UNKNOWN_2014-09-21
NICD1109_XXXX_SLE_Bombali_UNKNOWN_UNKNOWN_2014-09-19
NICD1136_XXXX_SLE_WesternRural_UNKNOWN_UNKNOWN_2014-09-23
NICD1142_XXXX_SLE_PortLoko_UNKNOWN_UNKNOWN_2014-09-22
NICD1143_XXXX_SLE_PortLoko_UNKNOWN_UNKNOWN_2014-09-23
NICD1227_XXXX_SLE_Bombali_UNKNOWN_UNKNOWN_2014-09-22
NICD1256-Vero_XXXX_SLE_Bombali_UNKNOWN_UNKNOWN_2014-09-22
NICD1261_XXXX_SLE_Bombali_UNKNOWN_UNKNOWN_2014-09-22

NICD1272-Vero_XXXX_SLE_Bombali_UNKNOWN_UNKNOWN_2014-09-22
NICD1329-Vero_XXXX_SLE_Bombali_UNKNOWN_UNKNOWN_2014-09-24
NICD3208-Vero_XXXX_SLE_WesternRural_UNKNOWN_UNKNOWN_2014-11-14
NICD3294_XXXX_SLE_WesternRural_UNKNOWN_UNKNOWN_2014-11-14
NICD3608-Vero_XXXX_SLE_WesternRural_UNKNOWN_UNKNOWN_2014-11-21
NICD3693-Vero_XXXX_SLE_WesternRural_UNKNOWN_UNKNOWN_2014-11-23
NICD4513-Vero_XXXX_SLE_WesternUrban_NewEngland_WestII_2014-12-09
NICD4670_XXXX_SLE_WesternUrban_TowerHill_CentralII_2014-12-12
NICD4672_XXXX_SLE_WesternUrban_Kortright_EastII_2014-12-12
NICD4691_XXXX_SLE_WesternUrban_NewEngland_WestII_2014-12-11
NICD4692_XXXX_SLE_WesternUrban_MountAureol_EastII_2014-12-13
PL2921_KU296506_SLE_Kambia_UNKNOWN_UNKNOWN_2015-02-03
**SL3.2.2**
NICD1049_XXXX_SLE_PortLoko_UNKNOWN_UNKNOWN_2014-09-21
J0073_KP759738_SLE_PortLoko_UNKNOWN_UNKNOWN_2014-10-12
J0142_KP759690_SLE_WesternUrban_UNKNOWN_UNKNOWN_2014-11-03
NICD0900_XXXX_SLE_PortLoko_UNKNOWN_UNKNOWN_2014-09-20
J0115_KP759597_SLE_PortLoko_UNKNOWN_UNKNOWN_2014-10-29
J0114_KP759596_SLE_PortLoko_UNKNOWN_UNKNOWN_2014-10-29
KT2315_KU296812_SLE_PortLoko_UNKNOWN_UNKNOWN_2014-12-02
KT2316_KU296774_SLE_PortLoko_UNKNOWN_UNKNOWN_2014-12-02
NICD0894_XXXX_SLE_PortLoko_UNKNOWN_UNKNOWN_2014-09-20
NICD0720-Vero_XXXX_SLE_PortLoko_UNKNOWN_UNKNOWN_2014-09-16
J0061_KP759637_SLE_PortLoko_UNKNOWN_UNKNOWN_2014-10-10
NICD0721_XXXX_SLE_PortLoko_UNKNOWN_UNKNOWN_2014-09-16
NICD0899_XXXX_SLE_PortLoko_UNKNOWN_UNKNOWN_2014-09-20
J0018_KP759644_SLE_WesternRural_UNKNOWN_UNKNOWN_2014-09-30
J0164_KP759623_SLE_WesternRural_UNKNOWN_UNKNOWN_2014-11-09
J0029_KP759688_SLE_PortLoko_UNKNOWN_UNKNOWN_2014-09-29
75750-EMLH_KU296464_SLE_WesternUrban_UNKNOWN_UNKNOWN_2015-01-17
J0030_KP759606_SLE_PortLoko_UNKNOWN_UNKNOWN_2014-10-03
J0011_KP759641_SLE_PortLoko_UNKNOWN_UNKNOWN_2014-09-29
NICD1396_XXXX_SLE_PortLoko_UNKNOWN_UNKNOWN_2014-09-25
20141288_KR653232_SLE_PortLoko_UNKNOWN_UNKNOWN_2014-09-23
NICD0878_XXXX_SLE_PortLoko_UNKNOWN_UNKNOWN_2014-09-20
NICD4795-swab_XXXX_SLE_WesternUrban_Freetown_WestII_2014-12-14
77581-EMLK_KU296540_SLE_WesternUrban_UNKNOWN_UNKNOWN_2015-01-03
77582-EMLK_KU296816_SLE_WesternUrban_UNKNOWN_UNKNOWN_2015-01-03
NICD5367-swab_XXXX_SLE_WesternUrban_Tengbehtown_WestIII_2014-12-27
77594-EMLK_KU296343_SLE_WesternUrban_UNKNOWN_UNKNOWN_2015-01-04
77706-EMLK_KU296572_SLE_WesternUrban_UNKNOWN_UNKNOWN_2015-01-10
J0140_KP759603_SLE_WesternRural_UNKNOWN_UNKNOWN_2014-10-30
20143036_KR653285_SLE_PortLoko_UNKNOWN_UNKNOWN_2014-10-24
J0012_KP759642_SLE_PortLoko_UNKNOWN_UNKNOWN_2014-09-29

J0014_KP759740_SLE_PortLoko_UNKNOWN_UNKNOWN_2014-09-29
J0013_KP759643_SLE_PortLoko_UNKNOWN_UNKNOWN_2014-09-28
NICD0338_XXXX_SLE_PortLoko_UNKNOWN_UNKNOWN_2014-09-08
NICD0424_XXXX_SLE_Tonkolili_UNKNOWN_UNKNOWN_2014-09-10
NICD3097_XXXX_SLE_PortLoko_UNKNOWN_UNKNOWN_2014-11-12
NICD2974-swab_XXXX_SLE_PortLoko_UNKNOWN_UNKNOWN_2014-11-08
75688-EMLH_KU296672_SLE_WesternRural_UNKNOWN_UNKNOWN_2015-01-14
362-EMLH_KU296505_SLE_WesternUrban_UNKNOWN_UNKNOWN_2015-01-21
NICD6079_XXXX_SLE_WesternUrban_Kortright_EastII_2015-01-17
J0163_KP759702_SLE_PortLoko_UNKNOWN_UNKNOWN_2014-11-08
PL4256_KU296509_SLE_PortLoko_UNKNOWN_UNKNOWN_2015-03-03
PL4127_KU296529_SLE_PortLoko_UNKNOWN_UNKNOWN_2015-02-28
PL5201_KU296612_SLE_PortLoko_UNKNOWN_UNKNOWN_2015-03-23
PL4028_KU296552_SLE_PortLoko_UNKNOWN_UNKNOWN_2015-02-26
PL4123_KU296752_SLE_PortLoko_UNKNOWN_UNKNOWN_2015-02-28
PL4322_KU296677_SLE_PortLoko_UNKNOWN_UNKNOWN_2015-03-05
PL4784_KU296660_SLE_PortLoko_UNKNOWN_UNKNOWN_2015-03-14
PL4125_KU296471_SLE_PortLoko_UNKNOWN_UNKNOWN_2015-02-28
PL4561_KU296725_SLE_PortLoko_UNKNOWN_UNKNOWN_2015-03-10
PL4330_KU296624_SLE_PortLoko_UNKNOWN_UNKNOWN_2015-03-05
PL4169_KU296828_SLE_PortLoko_UNKNOWN_UNKNOWN_2015-03-01
PL4126_KU296478_SLE_PortLoko_UNKNOWN_UNKNOWN_2015-02-28
PL4121_KU296305_SLE_PortLoko_UNKNOWN_UNKNOWN_2015-02-28
J0049_KP759717_SLE_PortLoko_UNKNOWN_UNKNOWN_2014-10-07
NICD0108_XXXX_SLE_PortLoko_UNKNOWN_UNKNOWN_2014-08-30
NICD0104_XXXX_SLE_WesternRural_UNKNOWN_UNKNOWN_2014-08-30
20140433_KR653246_SLE_Tonkolili_UNKNOWN_UNKNOWN_2014-09-03
G4956_KR105282_SLE_Tonkolili_UNKNOWN_UNKNOWN_2014-08-13
G4972_KR105285_SLE_Kenema_UNKNOWN_UNKNOWN_2014-08-14
**SL3.2.3**
G4999_KR105291_SLE_Kenema_UNKNOWN_UNKNOWN_2014-08-15
20140436_KR653287_SLE_Moyamba_UNKNOWN_UNKNOWN_2014-09-03
G5691_KR105318_SLE_Kenema_UNKNOWN_UNKNOWN_2014-09-14
G5520_KR105308_SLE_Kenema_UNKNOWN_UNKNOWN_2014-09-05
G5617_KR105312_SLE_Kenema_UNKNOWN_UNKNOWN_2014-09-10
20141491_KR653289_SLE_Kambia_UNKNOWN_UNKNOWN_2014-10-01
J0048_KP759716_SLE_WesternRural_UNKNOWN_UNKNOWN_2014-10-05
NICD0013_XXXX_SLE_WesternRural_UNKNOWN_UNKNOWN_2014-08-26
G4725_KR105267_SLE_Moyamba_UNKNOWN_UNKNOWN_2014-08-04
G5064_KR105297_SLE_Moyamba_UNKNOWN_UNKNOWN_2014-08-17
J0088_KP759648_SLE_PortLoko_UNKNOWN_UNKNOWN_2014-10-20
NICD1051_XXXX_SLE_PortLoko_UNKNOWN_UNKNOWN_2014-09-21
J0032_KP759691_SLE_PortLoko_UNKNOWN_UNKNOWN_2014-09-30
J0141_KP759689_SLE_WesternRural_UNKNOWN_UNKNOWN_2014-11-04

J0074_KP759739_SLE_WesternRural_UNKNOWN_UNKNOWN_2014-10-13
NICD3670_XXXX_SLE_WesternRural_UNKNOWN_UNKNOWN_2014-11-22
J0003_KP759651_SLE_PortLoko_UNKNOWN_UNKNOWN_2014-09-28
J0079_KP759748_SLE_WesternRural_UNKNOWN_UNKNOWN_2014-10-16
J0165_KP759703_SLE_WesternUrban_UNKNOWN_UNKNOWN_2014-11-08
NICD2521-swab_XXXX_SLE_WesternUrban_CalabaTown_EastIII_2014-10-23
NICD0856-Vero_XXXX_SLE_WesternRural_UNKNOWN_UNKNOWN_2014-09-20
J0118_KP759598_SLE_WesternRural_UNKNOWN_UNKNOWN_2014-10-31
20140091_KR653239_SLE_WesternRural_UNKNOWN_UNKNOWN_2014-08-22
NICD1043_XXXX_SLE_PortLoko_UNKNOWN_UNKNOWN_2014-09-22
20141282_KR653229_SLE_Kambia_UNKNOWN_UNKNOWN_2014-09-23
J0105_KP759766_SLE_PortLoko_UNKNOWN_UNKNOWN_2014-10-29
J0103_KP759658_SLE_PortLoko_UNKNOWN_UNKNOWN_2014-10-29
J0102_KP759657_SLE_PortLoko_UNKNOWN_UNKNOWN_2014-10-27
J0104_KP759765_SLE_PortLoko_UNKNOWN_UNKNOWN_2014-10-29
J0038_KP759618_SLE_PortLoko_UNKNOWN_UNKNOWN_2014-09-30
NICD0032_XXXX_SLE_Moyamba_UNKNOWN_UNKNOWN_2014-08-27
G5853_KR105329_SLE_Moyamba_UNKNOWN_UNKNOWN_2014-09-21
G5516_KR105307_SLE_Moyamba_UNKNOWN_UNKNOWN_2014-09-04
G5738_KR105322_SLE_Moyamba_UNKNOWN_UNKNOWN_2014-09-15
J0085_KP759645_SLE_PortLoko_UNKNOWN_UNKNOWN_2014-10-17
NICD0110_XXXX_SLE_PortLoko_UNKNOWN_UNKNOWN_2014-08-30
NICD0298_XXXX_SLE_PortLoko_UNKNOWN_UNKNOWN_2014-09-05
NICD2965-Vero_XXXX_SLE_PortLoko_UNKNOWN_UNKNOWN_2014-11-10
J0101_KP759656_SLE_PortLoko_UNKNOWN_UNKNOWN_2014-10-29
20144865_KR653231_SLE_PortLoko_UNKNOWN_UNKNOWN_2014-11-13
**SL3.2.4**
10402-EMLK_KU296635_SLE_WesternUrban_UNKNOWN_UNKNOWN_2015-02-10
10415-EMLK_KU296741_SLE_WesternUrban_UNKNOWN_UNKNOWN_2015-02-10
10454-EMLK_KU296679_SLE_WesternUrban_UNKNOWN_UNKNOWN_2015-02-12
10455-EMLK_KU296388_SLE_WesternUrban_UNKNOWN_UNKNOWN_2015-02-12
12004-EMLH_KU296302_SLE_WesternRural_UNKNOWN_UNKNOWN_2015-02-14
12178-EMLH_KU296525_SLE_WesternUrban_UNKNOWN_UNKNOWN_2015-02-22
12179-EMLH_KU296531_SLE_WesternUrban_UNKNOWN_UNKNOWN_2015-02-22
12180-EMLH_KU296310_SLE_WesternUrban_UNKNOWN_UNKNOWN_2015-02-22
12181-EMLH_KU296783_SLE_WesternUrban_UNKNOWN_UNKNOWN_2015-02-23
12189-EMLH_KU296369_SLE_WesternUrban_UNKNOWN_UNKNOWN_2015-02-23
12192-EMLH_KU296511_SLE_WesternUrban_UNKNOWN_UNKNOWN_2015-02-23
12193-EMLH_KU296616_SLE_WesternUrban_UNKNOWN_UNKNOWN_2015-02-23
12229-EMLH_KU296643_SLE_WesternRural_UNKNOWN_UNKNOWN_2015-02-27
12242-EMLH_KU296604_SLE_WesternUrban_UNKNOWN_UNKNOWN_2015-02-28
12257-EMLH_KU296702_SLE_PortLoko_UNKNOWN_UNKNOWN_2015-03-01
12275-EMLH_KU296538_SLE_WesternUrban_UNKNOWN_UNKNOWN_2015-03-02
12578-EMLH_KU296307_SLE_WesternRural_UNKNOWN_UNKNOWN_2015-03-20

12579-EMLH_KU296548_SLE_WesternRural_UNKNOWN_UNKNOWN_2015-03-20
12580-EMLH_KU296574_SLE_WesternRural_UNKNOWN_UNKNOWN_2015-03-20
12600-EMLH_KU296449_SLE_WesternUrban_UNKNOWN_UNKNOWN_2015-03-21
12628-EMLH_KU296692_SLE_WesternRural_UNKNOWN_UNKNOWN_2015-03-23
12634-EMLH_KU296653_SLE_WesternUrban_UNKNOWN_UNKNOWN_2015-03-24
12666-EMLH_KU296803_SLE_WesternRural_UNKNOWN_UNKNOWN_2015-03-26
12757-EMLH_KU296468_SLE_WesternUrban_UNKNOWN_UNKNOWN_2015-04-03
12767-EMLH_KU296768_SLE_WesternUrban_UNKNOWN_UNKNOWN_2015-04-04
12781-EMLH_KU296520_SLE_WesternUrban_UNKNOWN_UNKNOWN_2015-04-06
12851-EMLH_KU296671_SLE_WesternUrban_UNKNOWN_UNKNOWN_2015-04-15
12854-EMLH_KU296729_SLE_WesternUrban_UNKNOWN_UNKNOWN_2015-04-15
12855-EMLH_KU296723_SLE_WesternUrban_UNKNOWN_UNKNOWN_2015-04-15
12872-EMLH_KU296560_SLE_WesternUrban_UNKNOWN_UNKNOWN_2015-04-18
12916-EMLH_KU296318_SLE_WesternUrban_UNKNOWN_UNKNOWN_2015-04-29
12934-EMLH_KU296790_SLE_WesternUrban_UNKNOWN_UNKNOWN_2015-05-04
12936-EMLH_KU296561_SLE_WesternUrban_UNKNOWN_UNKNOWN_2015-05-04
13031-EMLH_KU296665_SLE_WesternUrban_UNKNOWN_UNKNOWN_2015-05-18
13204-EMLH_KU296544_SLE_WesternUrban_UNKNOWN_UNKNOWN_2015-06-16
13275-EMLH_KU296430_SLE_WesternUrban_UNKNOWN_UNKNOWN_2015-06-24
14521-EMLK_KU296755_SLE_WesternUrban_UNKNOWN_UNKNOWN_2015-02-13
14687-EMLK_KU296443_SLE_WesternUrban_UNKNOWN_UNKNOWN_2015-02-21
14782-EMLK_KU296639_SLE_Kambia_UNKNOWN_UNKNOWN_2015-04-23
14830-EMLK_KU296345_SLE_Kambia_UNKNOWN_UNKNOWN_2015-04-27
14888-EMLK_KU296423_SLE_Kambia_UNKNOWN_UNKNOWN_2015-05-03
15070R-DML__SLE_WesternUrban_UNKNOWN_UNKNOWN_2015-08-06
18596-EMLH_KU296715_SLE_WesternUrban_UNKNOWN_UNKNOWN_2015-07-05
18638R-EMLH_KU296348_SLE_WesternUrban_UNKNOWN_UNKNOWN_2015-07-08
18642R-EMLH_KU296357_SLE_WesternUrban_UNKNOWN_UNKNOWN_2015-07-08
18647-EMLH_KU296566_SLE_WesternUrban_UNKNOWN_UNKNOWN_2015-07-09
18648-EMLH_KU296701_SLE_WesternUrban_UNKNOWN_UNKNOWN_2015-07-09
18649-EMLH_KU296456_SLE_WesternUrban_UNKNOWN_UNKNOWN_2015-07-10
18650-EMLH_KU296370_SLE_WesternUrban_UNKNOWN_UNKNOWN_2015-07-10
18659R-EMLH_KU296585_SLE_WesternUrban_UNKNOWN_UNKNOWN_2015-07-10
18660-EMLH_KU296838_SLE_WesternUrban_UNKNOWN_UNKNOWN_2015-07-10
18687R-EMLH_KU296650_SLE_WesternUrban_UNKNOWN_UNKNOWN_2015-07-12
20140395_KR653279_SLE_Kono_UNKNOWN_UNKNOWN_2014-09-02
20141123_KR653291_SLE_Bo_UNKNOWN_UNKNOWN_2014-09-22
20141280_KR653276_SLE_Kambia_UNKNOWN_UNKNOWN_2014-09-23
20141582_KR653230_SLE_Bo_UNKNOWN_UNKNOWN_2014-10-04
20141650_KR653225_SLE_Kenema_UNKNOWN_UNKNOWN_2014-10-05
20143031_KR653275_SLE_WesternUrban_UNKNOWN_UNKNOWN_2014-10-25

20143187_KR653298_SLE_Pujehun_UNKNOWN_UNKNOWN_2014-10-28
20143716_KR653292_SLE_Moyamba_UNKNOWN_UNKNOWN_2014-11-04
20143753_KR653236_SLE_Tonkolili_UNKNOWN_UNKNOWN_2014-11-05
20143796_KR653257_SLE_Moyamba_UNKNOWN_UNKNOWN_2014-11-05
20144610_KR653270_SLE_Tonkolili_UNKNOWN_UNKNOWN_2014-11-12
20145853_KR653290_SLE_Pujehun_UNKNOWN_UNKNOWN_2014-11-26
389-EMLH_KU296593_SLE_WesternRural_UNKNOWN_UNKNOWN_2015-01-23
402-EMLH_KU296495_SLE_WesternRural_UNKNOWN_UNKNOWN_2015-01-23
443-EMLH_KU296539_SLE_WesternUrban_UNKNOWN_UNKNOWN_2015-01-26
489-EMLH_KU296324_SLE_WesternRural_UNKNOWN_UNKNOWN_2015-01-30
511-EMLH_KU296646_SLE_WesternUrban_UNKNOWN_UNKNOWN_2015-02-01
614-EMLH_KU296597_SLE_WesternRural_UNKNOWN_UNKNOWN_2015-02-05
632-EMLH_KU296693_SLE_WesternUrban_UNKNOWN_UNKNOWN_2015-02-06
634-EMLH_KU296799_SLE_WesternUrban_UNKNOWN_UNKNOWN_2015-02-06
731-EMLH_KU296765_SLE_WesternUrban_UNKNOWN_UNKNOWN_2015-02-11
741-EMLH_KU296295_SLE_WesternUrban_UNKNOWN_UNKNOWN_2015-02-11
75722-EMLH_KU296791_SLE_WesternRural_UNKNOWN_UNKNOWN_2015-01-15
75779-EMLH_KU296407_SLE_WesternUrban_UNKNOWN_UNKNOWN_2015-01-19
77573-EMLK_KU296516_SLE_WesternUrban_UNKNOWN_UNKNOWN_2015-01-03
77580-EMLK_KU296431_SLE_WesternUrban_UNKNOWN_UNKNOWN_2015-01-03
77589-EMLK_KU296403_SLE_WesternUrban_UNKNOWN_UNKNOWN_2015-01-04
77612-EMLK_KU296636_SLE_WesternUrban_UNKNOWN_UNKNOWN_2015-01-05
77613-EMLK_KU296579_SLE_WesternUrban_UNKNOWN_UNKNOWN_2015-01-05
77615-EMLK_KU296706_SLE_WesternUrban_UNKNOWN_UNKNOWN_2015-01-05
77630-EMLK_KU296779_SLE_WesternUrban_UNKNOWN_UNKNOWN_2015-01-06
77632-EMLK_KU296710_SLE_WesternUrban_UNKNOWN_UNKNOWN_2015-01-06
77643-EMLK_KU296395_SLE_WesternUrban_UNKNOWN_UNKNOWN_2015-01-07
77644-EMLK_KU296312_SLE_WesternUrban_UNKNOWN_UNKNOWN_2015-01-07
77648-EMLK_KU296620_SLE_WesternUrban_UNKNOWN_UNKNOWN_2015-01-07
77649-EMLK_KU296380_SLE_WesternUrban_UNKNOWN_UNKNOWN_2015-01-07
77710-EMLK_KU296696_SLE_WesternUrban_UNKNOWN_UNKNOWN_2015-01-10
77711-EMLK_KU296644_SLE_WesternUrban_UNKNOWN_UNKNOWN_2015-01-10
77712-EMLK_KU296400_SLE_WesternUrban_UNKNOWN_UNKNOWN_2015-01-10
77716-EMLK_KU296569_SLE_WesternUrban_UNKNOWN_UNKNOWN_2015-01-10
DML12033_KT357813_SLE_WesternUrban_UNKNOWN_UNKNOWN_2015-02-19
DML12051_KT357814_SLE_WesternUrban_UNKNOWN_UNKNOWN_2015-02-21
DML12116_KT357815_SLE_WesternUrban_UNKNOWN_UNKNOWN_2015-02-26
DML12117_KT357816_SLE_WesternUrban_UNKNOWN_UNKNOWN_2015-02-26
DML12120_KT357817_SLE_WesternUrban_UNKNOWN_UNKNOWN_2015-02-27
DML12137_KT357819_SLE_WesternUrban_UNKNOWN_UNKNOWN_2015-02-28
DML12194_KT357818_SLE_WesternUrban_UNKNOWN_UNKNOWN_2015-03-04
DML12239_KT357820_SLE_WesternUrban_UNKNOWN_UNKNOWN_2015-03-09
DML12260_KT357821_SLE_WesternUrban_UNKNOWN_UNKNOWN_2015-03-09
DML12268_KT357822_SLE_WesternUrban_UNKNOWN_UNKNOWN_2015-03-10
DML12485_KT357824_SLE_WesternUrban_UNKNOWN_UNKNOWN_2015-03-31

DML14077_KT357860_SLE_WesternUrban_UNKNOWN_UNKNOWN_2015-06-30
DML14163_KT357858_SLE_WesternUrban_UNKNOWN_UNKNOWN_2015-07-03
DML14366_KT357859_SLE_WesternUrban_UNKNOWN_UNKNOWN_2015-07-11
DML25180_KT357855_SLE_Kono_UNKNOWN_UNKNOWN_2015-02-23
DML25344_KT357856_SLE_Kono_UNKNOWN_UNKNOWN_2015-03-06
G5112_KR105298_SLE_Kenema_UNKNOWN_UNKNOWN_2014-08-19
G5114_KR105299_SLE_Pujehun_UNKNOWN_UNKNOWN_2014-08-18
G5119_KR105300_SLE_Kenema_UNKNOWN_UNKNOWN_2014-08-24
G5529_KR105309_SLE_Kono_UNKNOWN_UNKNOWN_2014-09-05
G5684_KR105316_SLE_Kenema_UNKNOWN_UNKNOWN_2014-09-13
G5685_KR105317_SLE_Kenema_UNKNOWN_UNKNOWN_2014-09-13
G5743_KR105323_SLE_Kono_UNKNOWN_UNKNOWN_2014-09-17
G5898_KR105331_SLE_Bo_UNKNOWN_UNKNOWN_2014-09-22
Goderich1_KT345616_SLE_WesternUrban_UNKNOWN_UNKNOWN_2015-02-19
J0004_KP759668_SLE_WesternRural_UNKNOWN_UNKNOWN_2014-09-28
J0006_KP759630_SLE_WesternRural_UNKNOWN_UNKNOWN_2014-09-28
J0026_KP759670_SLE_Bombali_UNKNOWN_UNKNOWN_2014-10-02
J0044_KP759713_SLE_WesternRural_UNKNOWN_UNKNOWN_2014-10-05
J0045_KP759714_SLE_WesternUrban_UNKNOWN_UNKNOWN_2014-10-05
J0047_KP759632_SLE_WesternUrban_UNKNOWN_UNKNOWN_2014-10-05
J0052_KP759720_SLE_WesternUrban_UNKNOWN_UNKNOWN_2014-10-08
J0059_KP759725_SLE_WesternRural_UNKNOWN_UNKNOWN_2014-10-08
J0060_KP759726_SLE_WesternUrban_UNKNOWN_UNKNOWN_2014-10-06
J0064_KP759728_SLE_WesternUrban_UNKNOWN_UNKNOWN_2014-10-09
J0065_KP759729_SLE_WesternRural_UNKNOWN_UNKNOWN_2014-10-09
J0068_KP759732_SLE_WesternRural_UNKNOWN_UNKNOWN_2014-10-09
J0069_KP759733_SLE_WesternRural_UNKNOWN_UNKNOWN_2014-10-09
J0075_KP759743_SLE_WesternRural_UNKNOWN_UNKNOWN_2014-10-17
J0077_KP759745_SLE_WesternRural_UNKNOWN_UNKNOWN_2014-10-17
J0078_KP759746_SLE_WesternUrban_UNKNOWN_UNKNOWN_2014-10-18
J0080_KP759749_SLE_PortLoko_UNKNOWN_UNKNOWN_2014-10-18
J0081_KP759750_SLE_WesternRural_UNKNOWN_UNKNOWN_2014-10-16
J0086_KP759646_SLE_WesternRural_UNKNOWN_UNKNOWN_2014-10-23
J0087_KP759647_SLE_PortLoko_UNKNOWN_UNKNOWN_2014-10-23
J0090_KP759649_SLE_WesternUrban_UNKNOWN_UNKNOWN_2014-10-23
J0093_KP759760_SLE_WesternRural_UNKNOWN_UNKNOWN_2014-10-24
J0094_KP759761_SLE_WesternRural_UNKNOWN_UNKNOWN_2014-10-27
J0098_KP759763_SLE_WesternRural_UNKNOWN_UNKNOWN_2014-10-28
J0100_KP759655_SLE_Kambia_UNKNOWN_UNKNOWN_2014-10-26
J0106_KP759767_SLE_WesternRural_UNKNOWN_UNKNOWN_2014-10-28
J0107_KP759768_SLE_WesternUrban_UNKNOWN_UNKNOWN_2014-10-29
J0108_KP759659_SLE_WesternRural_UNKNOWN_UNKNOWN_2014-10-28
J0116_KP759664_SLE_WesternRural_UNKNOWN_UNKNOWN_2014-10-30
J0117_KP759665_SLE_WesternRural_UNKNOWN_UNKNOWN_2014-10-31
J0129_KP759600_SLE_WesternUrban_UNKNOWN_UNKNOWN_2014-11-02

J0132_KP759601_SLE_WesternUrban_UNKNOWN_UNKNOWN_2014-11-02
J0133_KP759681_SLE_WesternRural_UNKNOWN_UNKNOWN_2014-10-31
J0136_KP759684_SLE_WesternRural_UNKNOWN_UNKNOWN_2014-11-01
J0148_KP759612_SLE_WesternRural_UNKNOWN_UNKNOWN_2014-11-06
J0151_KP759613_SLE_WesternRural_UNKNOWN_UNKNOWN_2014-11-07
J0153_KP759616_SLE_WesternRural_UNKNOWN_UNKNOWN_2014-11-07
J0167_KP759624_SLE_WesternUrban_UNKNOWN_UNKNOWN_2014-11-08
J0169_KP759706_SLE_WesternUrban_UNKNOWN_UNKNOWN_2014-11-09
J0170_KP759625_SLE_WesternUrban_UNKNOWN_UNKNOWN_2014-11-10
J0172_KP759626_SLE_WesternRural_UNKNOWN_UNKNOWN_2014-11-11
KT2317_KU296735_SLE_PortLoko_UNKNOWN_UNKNOWN_2014-12-02
KT2321_KU296762_SLE_PortLoko_UNKNOWN_UNKNOWN_2014-12-02
KT4631_KU296607_SLE_WesternUrban_UNKNOWN_UNKNOWN_2015-02-02
KT4677_KU296608_SLE_WesternRural_UNKNOWN_UNKNOWN_2015-02-03
KT4695_KU296549_SLE_WesternRural_UNKNOWN_UNKNOWN_2015-02-04
KT4935_KU296467_SLE_WesternRural_UNKNOWN_UNKNOWN_2015-02-11
KT5233_KU296750_SLE_WesternRural_UNKNOWN_UNKNOWN_2015-02-21
KT5249_KU296601_SLE_WesternUrban_UNKNOWN_UNKNOWN_2015-02-22
KT5382_KU296657_SLE_WesternRural_UNKNOWN_UNKNOWN_2015-02-25
KT5787_KU296711_SLE_WesternUrban_UNKNOWN_UNKNOWN_2015-03-07
KT5789_KU296713_SLE_WesternUrban_UNKNOWN_UNKNOWN_2015-03-07
MK10128_KU296502_SLE_Tonkolili_UNKNOWN_UNKNOWN_2015-07-31
MK10173_KU296313_SLE_Tonkolili_UNKNOWN_UNKNOWN_2015-08-01
MK2008_KU296526_SLE_WesternUrban_UNKNOWN_UNKNOWN_2015-02-12
MK2255_KU296648_SLE_Bombali_UNKNOWN_UNKNOWN_2015-02-21
MK2282_KU296788_SLE_Bombali_UNKNOWN_UNKNOWN_2015-02-22
MK2283_KU296833_SLE_Bombali_UNKNOWN_UNKNOWN_2015-02-22
MK2334_KU296366_SLE_Bombali_UNKNOWN_UNKNOWN_2015-02-23
MK2341_KU296494_SLE_Bombali_UNKNOWN_UNKNOWN_2015-02-24
MK2342_KU296347_SLE_Bombali_UNKNOWN_UNKNOWN_2015-02-24
MK2343_KU296640_SLE_Bombali_UNKNOWN_UNKNOWN_2015-02-24
MK2344_KU296586_SLE_Bombali_UNKNOWN_UNKNOWN_2015-02-24
MK2362_KU296842_SLE_Bombali_UNKNOWN_UNKNOWN_2015-02-24
MK2364_KU296719_SLE_Bombali_UNKNOWN_UNKNOWN_2015-02-24
MK2365_KU296363_SLE_Bombali_UNKNOWN_UNKNOWN_2015-02-25
MK2366_KU296810_SLE_Bombali_UNKNOWN_UNKNOWN_2015-02-25
MK2367_KU296652_SLE_Bombali_UNKNOWN_UNKNOWN_2015-02-25
MK2369_KU296832_SLE_Bombali_UNKNOWN_UNKNOWN_2015-02-25
MK2370_KU296760_SLE_Bombali_UNKNOWN_UNKNOWN_2015-02-25
MK2371_KU296397_SLE_Bombali_UNKNOWN_UNKNOWN_2015-02-25
MK2382_KU296686_SLE_Bombali_UNKNOWN_UNKNOWN_2015-02-25
MK2383_KU296583_SLE_Bombali_UNKNOWN_UNKNOWN_2015-02-25
MK2395_KU296336_SLE_Bombali_UNKNOWN_UNKNOWN_2015-02-25
MK2397_KU296444_SLE_Bombali_UNKNOWN_UNKNOWN_2015-02-25
MK2398_KU296578_SLE_Bombali_UNKNOWN_UNKNOWN_2015-02-25

MK2405_KU296542_SLE_Bombali_UNKNOWN_UNKNOWN_2015-02-25
MK2427_KU296759_SLE_Bombali_UNKNOWN_UNKNOWN_2015-02-25
MK2431_KU296562_SLE_Bombali_UNKNOWN_UNKNOWN_2015-02-25
MK2436_KU296626_SLE_Bombali_UNKNOWN_UNKNOWN_2015-02-26
MK2437_KU296469_SLE_Bombali_UNKNOWN_UNKNOWN_2015-02-26
MK2447_KU296642_SLE_Bombali_UNKNOWN_UNKNOWN_2015-02-26
MK2448_KU296818_SLE_Bombali_UNKNOWN_UNKNOWN_2015-02-26
MK2449_KU296466_SLE_Bombali_UNKNOWN_UNKNOWN_2015-02-26
MK2506_KU296360_SLE_Bombali_UNKNOWN_UNKNOWN_2015-02-27
MK2570_KU296346_SLE_Bombali_UNKNOWN_UNKNOWN_2015-03-01
MK2673_KU296749_SLE_Bombali_UNKNOWN_UNKNOWN_2015-03-03
MK2710_KU296703_SLE_Bombali_UNKNOWN_UNKNOWN_2015-03-05
MK2717_KU296425_SLE_Bombali_UNKNOWN_UNKNOWN_2015-03-04
MK2724_KU296695_SLE_Bombali_UNKNOWN_UNKNOWN_2015-03-05
MK2788_KU296573_SLE_Bombali_UNKNOWN_UNKNOWN_2015-03-07
MK2789_KU296613_SLE_Bombali_UNKNOWN_UNKNOWN_2015-03-07
MK2790_KU296452_SLE_Bombali_UNKNOWN_UNKNOWN_2015-03-07
MK2825_KU296402_SLE_Bombali_UNKNOWN_UNKNOWN_2015-03-08
MK2826_KU296405_SLE_Bombali_UNKNOWN_UNKNOWN_2015-03-08
MK2850_KU296374_SLE_Bombali_UNKNOWN_UNKNOWN_2015-03-09
MK2900_KU296787_SLE_Bombali_UNKNOWN_UNKNOWN_2015-03-10
MK2938_KU296771_SLE_Bombali_UNKNOWN_UNKNOWN_2015-03-11
MK2983_KU296393_SLE_Bombali_UNKNOWN_UNKNOWN_2015-03-12
MK3043_KU296474_SLE_Bombali_UNKNOWN_UNKNOWN_2015-03-14
MK3183_KU296800_SLE_Bombali_UNKNOWN_UNKNOWN_2015-03-17
MK3462_KU296565_SLE_Bombali_UNKNOWN_UNKNOWN_2015-03-26
MK3479_KU296483_SLE_Bombali_UNKNOWN_UNKNOWN_2015-03-26
MK8878_KU296684_SLE_Tonkolili_UNKNOWN_UNKNOWN_2015-07-23
MK9396_KU296321_SLE_WesternUrban_UNKNOWN_UNKNOWN_2015-07-18
NICD0019_XXXX_SLE_WesternRural_UNKNOWN_UNKNOWN_2014-08-26
NICD1526_XXXX_SLE_WesternRural_UNKNOWN_UNKNOWN_2014-10-01
NICD1972-Vero_XXXX_SLE_WesternUrban_Kissy_EastIII_2014-10-09
NICD2118_XXXX_SLE_WesternUrban_Aberdeen_WestIII_2014-10-11
NICD2540-Vero_XXXX_SLE_WesternUrban_AllenTown_EastIII_2014-10-22
NICD2542_XXXX_SLE_WesternUrban_AllenTown_EastIII_2014-10-22
NICD2749_XXXX_SLE_Kambia_UNKNOWN_UNKNOWN_2014-11-06
NICD3465-swab_XXXX_SLE_WesternUrban_AllenTown_EastIII_2014-11-18
NICD3666_XXXX_SLE_WesternRural_UNKNOWN_UNKNOWN_2014-11-22
NICD3687-Vero_XXXX_SLE_WesternUrban_Magazine_CentralI_2014-11-23
NICD3752_XXXX_SLE_WesternRural_UNKNOWN_UNKNOWN_2014-11-24
NICD3753_XXXX_SLE_WesternRural_UNKNOWN_UNKNOWN_2014-11-24
NICD3824_XXXX_SLE_WesternRural_UNKNOWN_UNKNOWN_2014-11-25
NICD3944_XXXX_SLE_WesternUrban_Wellington_EastIII_2014-11-26
NICD3954_XXXX_SLE_WesternUrban_Wellington_EastIII_2014-11-26
NICD4024-Vero_XXXX_SLE_WesternUrban_Kortright_EastII_2014-11-29

NICD4027-Vero_XXXX_SLE_WesternUrban_Kortright_EastII_2014-11-29
NICD4029-Vero_XXXX_SLE_WesternUrban_Kortright_EastII_2014-11-29
NICD4041-Vero_XXXX_SLE_WesternUrban_Lumley_WestIII_2014-11-29
NICD4122_XXXX_SLE_WesternRural_UNKNOWN_UNKNOWN_2014-12-02
NICD4125_XXXX_SLE_WesternUrban_NewEngland_WestII_2014-12-02
NICD4167_XXXX_SLE_WesternUrban_Kortright_EastII_2014-12-02
NICD4325_XXXX_SLE_WesternRural_UNKNOWN_UNKNOWN_2014-12-05
NICD4361-Vero_XXXX_SLE_WesternRural_UNKNOWN_UNKNOWN_2014-12-06
NICD4430_XXXX_SLE_WesternUrban_Magazine_CentralI_2014-12-06
NICD4674_XXXX_SLE_WesternRural_UNKNOWN_UNKNOWN_2014-12-12
NICD4693_XXXX_SLE_WesternUrban_Wellington_EastIII_2014-12-10
NICD4735_XXXX_SLE_WesternUrban_Freetown_WestII_2014-12-13
NICD4823_XXXX_SLE_WesternUrban_Magazine_CentralI_2014-12-15
NICD4877_XXXX_SLE_WesternUrban_CongoTown_WestI_2014-12-16
NICD4932-Vero_XXXX_SLE_WesternUrban_FoulahTown_EastII_2014-12-17
NICD4975_XXXX_SLE_WesternUrban_Magazine_CentralI_2014-12-18
NICD5071_XXXX_SLE_WesternUrban_Magazine_CentralI_2014-12-20
NICD5386_XXXX_SLE_WesternUrban_Freetown_WestII_2014-12-29
NICD5388_XXXX_SLE_WesternUrban_NewEngland_WestII_2014-12-29
NICD5395_XXXX_SLE_WesternUrban_Kortright_EastII_2014-12-29
NICD5400_XXXX_SLE_WesternUrban_Kortright_EastII_2014-12-28
NICD5647-Vero_XXXX_SLE_WesternUrban_Wellington_EastIII_2015-01-03
NICD5747_XXXX_SLE_WesternUrban_TowerHill_CentralII_2015-01-08
NICD5819_XXXX_SLE_WesternUrban_Lumley_WestIII_2015-01-09
NICD6195_XXXX_SLE_WesternUrban_CalabaTown_EastIII_2015-01-23
NICD6345_XXXX_SLE_WesternUrban_MountAureol_EastII_2015-01-27
NICD6348_XXXX_SLE_WesternUrban_MountAureol_EastII_2015-01-28
NICD6392-Vero_XXXX_SLE_WesternUrban_MountAureol_EastII_2015-01-30
NICD6403_XXXX_SLE_WesternUrban_MountAureol_EastII_2015-01-29
NICD6404_XXXX_SLE_WesternUrban_MountAureol_EastII_2015-01-29
NICD6414_XXXX_SLE_WesternUrban_CongoTown_WestI_2015-01-30
NICD6564_XXXX_SLE_WesternUrban_Aberdeen_WestIII_2015-02-07
NICD6587_XXXX_SLE_WesternUrban_AllenTown_EastIII_2015-02-10
NICD6601_XXXX_SLE_WesternUrban_Freetown_WestII_2015-02-11
NICD6605_XXXX_SLE_WesternUrban_Aberdeen_WestIII_2015-02-11
NICD6640_XXXX_SLE_WesternUrban_Magazine_CentralI_2015-02-13
NICD6643_XXXX_SLE_WesternUrban_Freetown_WestII_2015-02-13
NICD6644_XXXX_SLE_WesternUrban_Freetown_WestII_2015-02-13
NICD6672_XXXX_SLE_WesternUrban_MountAureol_EastII_2015-02-14
NICD7034_XXXX_SLE_WesternUrban_Aberdeen_WestIII_2015-03-06
NICD7036_XXXX_SLE_WesternUrban_Aberdeen_WestIII_2015-03-07
PL2101_KU296714_SLE_PortLoko_UNKNOWN_UNKNOWN_2015-01-18
PL2668_KU296294_SLE_PortLoko_UNKNOWN_UNKNOWN_2015-01-29
PL3984_KU296330_SLE_PortLoko_UNKNOWN_UNKNOWN_2015-02-25
PL4059_KU296485_SLE_PortLoko_UNKNOWN_UNKNOWN_2015-02-26

PL4105_KU296694_SLE_PortLoko_UNKNOWN_UNKNOWN_2015-02-28
PL4187_KU296567_SLE_PortLoko_UNKNOWN_UNKNOWN_2015-03-01
PL4190_KU296351_SLE_PortLoko_UNKNOWN_UNKNOWN_2015-03-01
PL4194_KU296501_SLE_PortLoko_UNKNOWN_UNKNOWN_2015-03-02
PL4292_KU296827_SLE_Kambia_UNKNOWN_UNKNOWN_2015-03-03
PL4346_KU296641_SLE_Kambia_UNKNOWN_UNKNOWN_2015-03-04
PL4569_KU296598_SLE_PortLoko_UNKNOWN_UNKNOWN_2015-03-10
PL4570_KU296451_SLE_PortLoko_UNKNOWN_UNKNOWN_2015-03-10
PL4651_KU296841_SLE_PortLoko_UNKNOWN_UNKNOWN_2015-03-12
PL4671_KU296782_SLE_PortLoko_UNKNOWN_UNKNOWN_2015-03-12
PL4696_KU296513_SLE_PortLoko_UNKNOWN_UNKNOWN_2015-03-13
PL5001_KU296514_SLE_PortLoko_UNKNOWN_UNKNOWN_2015-03-19
PL5019_KU296627_SLE_PortLoko_UNKNOWN_UNKNOWN_2015-03-19
PL5099_KU296682_SLE_PortLoko_UNKNOWN_UNKNOWN_2015-03-19
PL5179_KU296638_SLE_PortLoko_UNKNOWN_UNKNOWN_2015-03-22
PL5202_KU296396_SLE_PortLoko_UNKNOWN_UNKNOWN_2015-03-23
PL5228_KU296535_SLE_PortLoko_UNKNOWN_UNKNOWN_2015-03-23
PL5868_KU296550_SLE_Kambia_UNKNOWN_UNKNOWN_2015-04-06
PL6086_KU296651_SLE_Kambia_UNKNOWN_UNKNOWN_2015-04-12
**SL3.2.5**
12615-EMLH_KU296521_SLE_WesternRural_UNKNOWN_UNKNOWN_2015-03-22
12705-EMLH_KU296490_SLE_WesternUrban_UNKNOWN_UNKNOWN_2015-03-29
20140024_KR653252_SLE_PortLoko_UNKNOWN_UNKNOWN_2014-08-20
20141271_KR653261_SLE_PortLoko_UNKNOWN_UNKNOWN_2014-09-24
20141429_KR653260_SLE_Kono_UNKNOWN_UNKNOWN_2014-09-28
20142551_KR653226_SLE_Koinadugu_UNKNOWN_UNKNOWN_2014-10-23
20142895_KR653268_SLE_Koinadugu_UNKNOWN_UNKNOWN_2014-10-24
20143018_KR653272_SLE_WesternUrban_UNKNOWN_UNKNOWN_2014-10-26
20143360_KR653258_SLE_Kenema_UNKNOWN_UNKNOWN_2014-10-30
20143415_KR653248_SLE_WesternRural_UNKNOWN_UNKNOWN_2014-10-31
20143550_KR653228_SLE_Bombali_UNKNOWN_UNKNOWN_2014-11-01
20143648_KR653253_SLE_Koinadugu_UNKNOWN_UNKNOWN_2014-11-03
20143659_KR653256_SLE_Koinadugu_UNKNOWN_UNKNOWN_2014-11-03
20143918_KR653240_SLE_WesternRural_UNKNOWN_UNKNOWN_2014-11-06
20143938_KR653264_SLE_Koinadugu_UNKNOWN_UNKNOWN_2014-11-07
20144819_KR653299_SLE_Moyamba_UNKNOWN_UNKNOWN_2014-11-15
20144820_KR653243_SLE_Moyamba_UNKNOWN_UNKNOWN_2014-11-15
20146578_KR653281_SLE_Koinadugu_UNKNOWN_UNKNOWN_2014-12-26
75711-EMLH_KU296596_SLE_WesternUrban_UNKNOWN_UNKNOWN_2015-01-15
77585-EMLK_KU296734_SLE_WesternUrban_UNKNOWN_UNKNOWN_2015-01-04
DML12458_KT357823_SLE_WesternUrban_UNKNOWN_UNKNOWN_2015-03-28
G5019_KR105294_SLE_Kenema_UNKNOWN_UNKNOWN_2014-08-16
G6089_KR105345_SLE_Tonkolili_UNKNOWN_UNKNOWN_2014-09-27
G6091_KR105346_SLE_Tonkolili_UNKNOWN_UNKNOWN_2014-09-27

G6095_KR105347_SLE_Tonkolili_UNKNOWN_UNKNOWN_2014-09-27
J0009_KP759734_SLE_PortLoko_UNKNOWN_UNKNOWN_2014-09-28
J0010_KP759639_SLE_PortLoko_UNKNOWN_UNKNOWN_2014-09-28
J0023_KP759652_SLE_WesternRural_UNKNOWN_UNKNOWN_2014-10-01
J0025_KP759666_SLE_WesternRural_UNKNOWN_UNKNOWN_2014-10-02
J0027_KP759678_SLE_Bombali_UNKNOWN_UNKNOWN_2014-10-02
J0031_KP759607_SLE_PortLoko_UNKNOWN_UNKNOWN_2014-10-03
J0033_KP759692_SLE_PortLoko_UNKNOWN_UNKNOWN_2014-10-03
J0034_KP759608_SLE_PortLoko_UNKNOWN_UNKNOWN_2014-09-30
J0035_KP759609_SLE_PortLoko_UNKNOWN_UNKNOWN_2014-10-01
J0036_KP759694_SLE_PortLoko_UNKNOWN_UNKNOWN_2014-10-03
J0037_KP759615_SLE_PortLoko_UNKNOWN_UNKNOWN_2014-10-03
J0046_KP759715_SLE_WesternRural_UNKNOWN_UNKNOWN_2014-10-07
J0055_KP759723_SLE_PortLoko_UNKNOWN_UNKNOWN_2014-10-06
J0056_KP759634_SLE_PortLoko_UNKNOWN_UNKNOWN_2014-10-08
J0057_KP759635_SLE_PortLoko_UNKNOWN_UNKNOWN_2014-10-05
J0058_KP759724_SLE_PortLoko_UNKNOWN_UNKNOWN_2014-10-08
J0062_KP759727_SLE_PortLoko_UNKNOWN_UNKNOWN_2014-10-10
J0063_KP759638_SLE_PortLoko_UNKNOWN_UNKNOWN_2014-10-10
J0067_KP759731_SLE_PortLoko_UNKNOWN_UNKNOWN_2014-10-09
J0070_KP759735_SLE_PortLoko_UNKNOWN_UNKNOWN_2014-10-09
J0071_KP759736_SLE_PortLoko_UNKNOWN_UNKNOWN_2014-10-09
J0072_KP759737_SLE_PortLoko_UNKNOWN_UNKNOWN_2014-10-09
J0076_KP759744_SLE_WesternRural_UNKNOWN_UNKNOWN_2014-10-17
J0082_KP759751_SLE_WesternRural_UNKNOWN_UNKNOWN_2014-10-16
J0083_KP759752_SLE_PortLoko_UNKNOWN_UNKNOWN_2014-10-18
J0084_KP759753_SLE_PortLoko_UNKNOWN_UNKNOWN_2014-10-16
J0095_KP759653_SLE_WesternUrban_UNKNOWN_UNKNOWN_2014-10-27
J0097_KP759762_SLE_WesternRural_UNKNOWN_UNKNOWN_2014-10-28
J0109_KP759594_SLE_WesternUrban_UNKNOWN_UNKNOWN_2014-10-31
J0110_KP759660_SLE_WesternUrban_UNKNOWN_UNKNOWN_2014-10-31
J0111_KP759595_SLE_WesternUrban_UNKNOWN_UNKNOWN_2014-10-30
J0119_KP759667_SLE_WesternUrban_UNKNOWN_UNKNOWN_2014-10-30
J0120_KP759599_SLE_WesternRural_UNKNOWN_UNKNOWN_2014-10-30
J0122_KP759671_SLE_WesternUrban_UNKNOWN_UNKNOWN_2014-10-29
J0125_KP759674_SLE_WesternRural_UNKNOWN_UNKNOWN_2014-10-31
J0126_KP759675_SLE_WesternRural_UNKNOWN_UNKNOWN_2014-10-31
J0127_KP759676_SLE_WesternUrban_UNKNOWN_UNKNOWN_2014-10-30
J0128_KP759677_SLE_WesternUrban_UNKNOWN_UNKNOWN_2014-10-31
J0130_KP759679_SLE_PortLoko_UNKNOWN_UNKNOWN_2014-11-02
J0131_KP759680_SLE_PortLoko_UNKNOWN_UNKNOWN_2014-10-30
J0138_KP759686_SLE_WesternUrban_UNKNOWN_UNKNOWN_2014-11-01
J0139_KP759687_SLE_WesternUrban_UNKNOWN_UNKNOWN_2014-10-30
J0145_KP759693_SLE_WesternRural_UNKNOWN_UNKNOWN_2014-11-06
J0158_KP759700_SLE_WesternRural_UNKNOWN_UNKNOWN_2014-11-08

J0161_KP759701_SLE_WesternUrban_UNKNOWN_UNKNOWN_2014-11-08
J0166_KP759704_SLE_WesternUrban_UNKNOWN_UNKNOWN_2014-11-08
KT5320_KU296809_SLE_WesternRural_UNKNOWN_UNKNOWN_2015-02-24
KT5388_KU296355_SLE_WesternRural_UNKNOWN_UNKNOWN_2015-02-25
KT5734_KU296595_SLE_WesternRural_UNKNOWN_UNKNOWN_2015-03-06
MK0020_KU296551_SLE_Bombali_UNKNOWN_UNKNOWN_2014-12-08
MK0592_KU296814_SLE_Bombali_UNKNOWN_UNKNOWN_2014-12-28
MK0704_KU296663_SLE_Bombali_UNKNOWN_UNKNOWN_2014-12-31
MK0725_KU296629_SLE_Bombali_UNKNOWN_UNKNOWN_2015-01-01
MK0879_KU296410_SLE_PortLoko_UNKNOWN_UNKNOWN_2015-01-05
MK0894_KU296356_SLE_Bombali_UNKNOWN_UNKNOWN_2015-01-06
MK1156_KU296605_SLE_Bombali_UNKNOWN_UNKNOWN_2015-01-15
MK13013_KU296454_SLE_Bombali_UNKNOWN_UNKNOWN_2015-09-12
MK2656_KU296577_SLE_Koinadugu_UNKNOWN_UNKNOWN_2015-03-03
MK2918_KU296300_SLE_Koinadugu_UNKNOWN_UNKNOWN_2015-03-10
MK3050_KU296445_SLE_Koinadugu_UNKNOWN_UNKNOWN_2015-03-14
NICD0086_XXXX_SLE_Bombali_UNKNOWN_UNKNOWN_2014-08-29
NICD0129_XXXX_SLE_Bombali_UNKNOWN_UNKNOWN_2014-08-31
NICD0188_XXXX_SLE_Bombali_UNKNOWN_UNKNOWN_2014-09-03
NICD0190_XXXX_SLE_Bombali_UNKNOWN_UNKNOWN_2014-09-03
NICD0193_XXXX_SLE_Bombali_UNKNOWN_UNKNOWN_2014-09-03
NICD0234_XXXX_SLE_PortLoko_UNKNOWN_UNKNOWN_2014-09-04
NICD0235_XXXX_SLE_PortLoko_UNKNOWN_UNKNOWN_2014-09-04
NICD0256_XXXX_SLE_Bombali_UNKNOWN_UNKNOWN_2014-09-04
NICD0257_XXXX_SLE_Bombali_UNKNOWN_UNKNOWN_2014-09-05
NICD0264_XXXX_SLE_Bombali_UNKNOWN_UNKNOWN_2014-09-05
NICD0299_XXXX_SLE_PortLoko_UNKNOWN_UNKNOWN_2014-09-05
NICD0369_XXXX_SLE_Bombali_UNKNOWN_UNKNOWN_2014-09-07
NICD0637_XXXX_SLE_Tonkolili_UNKNOWN_UNKNOWN_2014-09-16
NICD0650_XXXX_SLE_Bombali_UNKNOWN_UNKNOWN_2014-09-15
NICD0782_XXXX_SLE_Bombali_UNKNOWN_UNKNOWN_2014-09-17
NICD0919_XXXX_SLE_Tonkolili_UNKNOWN_UNKNOWN_2014-09-19
NICD1016_XXXX_SLE_PortLoko_UNKNOWN_UNKNOWN_2014-09-21
NICD1079_XXXX_SLE_Bombali_UNKNOWN_UNKNOWN_2014-09-20
NICD1089-Vero_XXXX_SLE_Bombali_UNKNOWN_UNKNOWN_2014-09-20
NICD1100_XXXX_SLE_Bombali_UNKNOWN_UNKNOWN_2014-09-21
NICD1147_XXXX_SLE_PortLoko_UNKNOWN_UNKNOWN_2014-09-22
NICD1148_XXXX_SLE_PortLoko_UNKNOWN_UNKNOWN_2014-09-22
NICD1156_XXXX_SLE_PortLoko_UNKNOWN_UNKNOWN_2014-09-22
NICD1167_XXXX_SLE_PortLoko_UNKNOWN_UNKNOWN_2014-09-22
NICD1201_XXXX_SLE_Tonkolili_UNKNOWN_UNKNOWN_2014-09-20
NICD1317_XXXX_SLE_Bombali_UNKNOWN_UNKNOWN_2014-09-24
NICD1422_XXXX_SLE_PortLoko_UNKNOWN_UNKNOWN_2014-09-26
NICD1430_XXXX_SLE_PortLoko_UNKNOWN_UNKNOWN_2014-09-26
NICD1514_XXXX_SLE_PortLoko_UNKNOWN_UNKNOWN_2014-09-28

NICD1662-Vero_XXXX_SLE_Bombali_UNKNOWN_UNKNOWN_2014-10-01
NICD2219_XXXX_SLE_WesternUrban_AllenTown_EastIII_2014-10-14
NICD2308_XXXX_SLE_WesternRural_UNKNOWN_UNKNOWN_2014-10-16
NICD2310_XXXX_SLE_WesternRural_UNKNOWN_UNKNOWN_2014-10-16
NICD2494_XXXX_SLE_WesternRural_UNKNOWN_UNKNOWN_2014-10-23
NICD2589_XXXX_SLE_WesternRural_UNKNOWN_UNKNOWN_2014-10-24
NICD3304-swab_XXXX_SLE_WesternUrban_Kortright_EastII_2014-11-15
NICD3341_XXXX_SLE_WesternUrban_Magazine_CentralI_2014-11-16
NICD3420_XXXX_SLE_WesternUrban_Kissy_EastIII_2014-11-17
NICD4030-Vero_XXXX_SLE_WesternRural_UNKNOWN_UNKNOWN_2014-11-29
NICD4072-swab_XXXX_SLE_WesternUrban_AllenTown_EastIII_2014-11-30
NICD4113_XXXX_SLE_WesternUrban_Kissy_EastIII_2014-12-01
NICD4329_XXXX_SLE_WesternUrban_FoulahTown_EastII_2014-12-05
NICD4356_XXXX_SLE_WesternUrban_Wilberforce_WestIII_2014-12-06
NICD5291_XXXX_SLE_WesternUrban_Kortright_EastII_2014-12-25
NICD5293_XXXX_SLE_WesternUrban_Magazine_CentralI_2014-12-25
NICD5311_XXXX_SLE_WesternUrban_Freetown_WestII_2014-12-26
NICD5370_XXXX_SLE_WesternRural_UNKNOWN_UNKNOWN_2014-12-28
NICD5421_XXXX_SLE_WesternUrban_Wellington_EastIII_2014-12-30
NICD6823-swab_XXXX_SLE_WesternUrban_AllenTown_EastIII_2015-02-26

Table S4. Summary of the geographical distribution of SLE EVD cases from which sequences were available in the public domain or generated in this study. The percentage coverage of sequences available in relation to total number of laboratory confirmed cases are also provided.

**Sierra Leone districts**	**Population size (as of December 2015)†**	**Total number of laboratory confirmed cases**	**Number of sequences available Dudas dataset (% of confirmed cases)**	**Number of sequences NICD data (% of confirmed cases)**	**New number of sequences available (% of confirmed cases)**
Bo	575478	358	13 (3.63%)	0	13 (3.63%)
Bombali	606544	1064	108 (10.15%)	29 (2.73%)	137 (12.88%)
Bonthe	200781	1	1 (100%)	0	1 (100%)
Kailahun	526379	658	101 (15.35%)	0	101 (15.35%)
Kambia	345474	277	74 (26.71%)	2 (0.72%)	76 (27.44%)
Kenema	609891	532	75 (14.1%)	0	75 (14.1%)
Koinadugu	409372	155	11 (7.1%)	0	11 (7.1%)
Kono	506100	450	38 (8.44%)	0	38 (8.44%)
Moyamba	318588	276	23 (8.33%)	1 (0.36%)	24 (8.7%)
Port Loko	615376	1609	149 (9.26%)	33 (2.05%)	182 (11.31%)
Pujehun	346461	54	9 (16.67%)	0	9 (16.67%)
Tonkolili	531435	505	19 (3.76%)	10 (1.98%)	29 (5.74%)
Western Area Rural	444270	1381	88 (6.37%)	34 (2.46%)	122 (8.83%)
Western Area Urban	1055964	2520	152 (6.03%)	109 (4.33%)	261 (10.36%)
**Total**	**7092113**	**9840**	**861* (8.75%)**	**218 (2.22%)**	**1079* (10.97%)**

† https://www.citypopulation.de/php/sierraleone-admin.php

* A total of 167 sequences from the Dudas dataset did not have complete information on district of origin.

Table S5. Temporal distribution of sequence numbers from the Western Area for the period August 2014 to March 2015

Month	Number of laboratory confirmed cases in Western Area Rural	Number of sequences available from Western Area Rural Dudas dataset (% of confirmed cases)	Number of sequences from Western Area Rural NICD data (% of confirmed cases)	New number of sequences available from Western Area Rural (% of confirmed cases)	Number of laboratory confirmed cases in Western Area Urban	Number of sequences available from Western Area Urban Dudas dataset (% of confirmed cases)	Number of sequences from Western Area Urban NICD data (% of confirmed cases)	New number of sequences available from Western Area Urban (% of confirmed cases)
August 2014	47	1 (2.13%)	3 (6.38%)	**4 (8.51%)**	61	1 (1.64%)	4 (6.56%)	**5 (8.20%)**
September 2014	165	8 (4.85%)	4 (2.42%)	**12 (7.27%)**	174	3 (1.72%)	5 (2.87%)	**8 (4.60%)**
October 2014	342	49 (14.33%)	5 (1.46%)	**54 (15.79%)**	342	38 (11.11%)	10 (2.92%)	**48 (14.04%)**
November 2014	434	28 (6.45%)	13 (2.99%)	**41 (9.45%)**	715	20 (2.80%)	25 (3.50%)	**45 (6.29%)**
December 2014	167	0	6 (3.59%)	**6 (3.59%)**	620	1 (0.16%)	39 (6.29%)	**40 (6.45%)**
January 2015	150	5 (3.33%)	3 (2.0%)	**8 (5.33%)**	334	36 (10.78%)	15 (4.49%)	**51 (15.27%)**
February 2015	35	12 (34.29%)	0	12 (34.29%)	134	30 (22.39%)	9 (6.72%)	**39 (29.10%)**
March 2015	23	7 (30.43%)	0	7 (30.43%)	71	12 (16.90%)	2 (2.82%)	**14 (19.72%)**

Table S6. The most recent common ancestors (TMRCA) for the 7 main sub-lineages and information on duration of circulation

Lineage	Common ancestor location (probability)	TMRCA Median [Lo,Hi]	Lineage length in years Median [Lo,Hi]	Lineage length in days Median [Lo,Hi]
SL 3.1.1	Kenema (72.5%)<br>WAR (20.7%)	2014/08/06<br>[2014.53,2014.63]	1.09 [1.05,1.15]	396.26<br>[382.37,421.33]
SL 3.1.2	WAR (69.9%)<br>WAU (27.7%)	2014/08/03<br>[2014.55,2014.62]	0.67 [0.65,0.71]	246.34<br>[235.66,260.19]
SL 3.2.1	Bombali (84.5%)<br>Kenema (27.7%)	2014/07/30<br>[2014.52,2014.62]	0.71 [0.67,0.76]	258.21<br>[244.04,278.98]
SL 3.2.2	Kenema (97.2%)	2014/07/23<br>[2014.50,2014.60]	0.66 [0.62,0.72]	240.83<br>[226.36,262.26]
SL 3.2.3	Kenema (98.6%)	2014/07/12<br>[2014.49,2014.57]	0.36 [0.33,0.40]	132.47<br>[118.63,147.16]
SL 3.2.4	Kenema (87.9%)<br>WAR (11.7%)	2014/07/16<br>[2014.48,2014.59]	1.05 [1.00,1.11]	383.27<br>[366.18,405.55]
SL 3.2.5	Kenema (98.9%)	2014/07/23<br>[2014.52,2014.60]	1.13 [1.09,1.18]	412.96<br>[398.29,431.02]

Table S7. Mutations defining the 7 sub-lineages of SL3

Lineage	Defining mutation(s)
SL 3.1.1	C18354T
SL 3.1.2	A7148G; A17445G; C18162T
SL 3.2.1	G18240T
SL 3.2.2	T16231C
SL 3.2.3	A6726G; C15801T; T18081C
SL 3.2.4	T5849C
SL 3.2.5	T3008C; T3011C

Table S7. Mutations defining the 7 sub-lineages of SL3

Lineage	Defining mutation(s)
SL 3.1.1	C18354T
SL 3.1.2	A7148G; A17445G; C18162T
SL 3.2.1	G18240T
SL 3.2.2	T16231C
SL 3.2.3	A6726G; C15801T; T18081C
SL 3.2.4	T5849C
SL 3.2.5	T3008C; T3011C

Table S6. The most recent common ancestors (TMRCA) for the 7 main sub-lineages and information on duration of circulation

Lineage	Common ancestor location (probability)	TMRCA Median [Lo,Hi]	Lineage length in years Median [Lo,Hi]	Lineage length in days Median [Lo,Hi]
SL 3.1.1	Kenema (72.5%)<br>WAR (20.7%)	2014/08/06<br>[2014.53,2014.63]	1.09 [1.05,1.15]	396.26<br>[382.37,421.33]
SL 3.1.2	WAR (69.9%)<br>WAU (27.7%)	2014/08/03<br>[2014.55,2014.62]	0.67 [0.65,0.71]	246.34<br>[235.66,260.19]
SL 3.2.1	Bombali (84.5%)<br>Kenema (27.7%)	2014/07/30<br>[2014.52,2014.62]	0.71 [0.67,0.76]	258.21<br>[244.04,278.98]
SL 3.2.2	Kenema (97.2%)	2014/07/23<br>[2014.50,2014.60]	0.66 [0.62,0.72]	240.83<br>[226.36,262.26]
SL 3.2.3	Kenema (98.6%)	2014/07/12<br>[2014.49,2014.57]	0.36 [0.33,0.40]	132.47<br>[118.63,147.16]
SL 3.2.4	Kenema (87.9%)<br>WAR (11.7%)	2014/07/16<br>[2014.48,2014.59]	1.05 [1.00,1.11]	383.27<br>[366.18,405.55]
SL 3.2.5	Kenema (98.9%)	2014/07/23<br>[2014.52,2014.60]	1.13 [1.09,1.18]	412.96<br>[398.29,431.02]

Table S7. Mutations defining the 7 sub-lineages of SL3

Lineage	Defining mutation(s)
SL 3.1.1	C18354T
SL 3.1.2	A7148G; A17445G; C18162T
SL 3.2.1	G18240T
SL 3.2.2	T16231C
SL 3.2.3	A6726G; C15801T; T18081C
SL 3.2.4	T5849C
SL 3.2.5	T3008C; T3011C
