# Supplementary figures and images for: Phylodynamic Analysis of Ebola Virus Disease Transmission in Sierra Leone
Source: Viruses. 2019 Jan 16;11(1):71. doi: 10.3390/v11010071 (PMC6356631; doi:10.3390/v11010071)

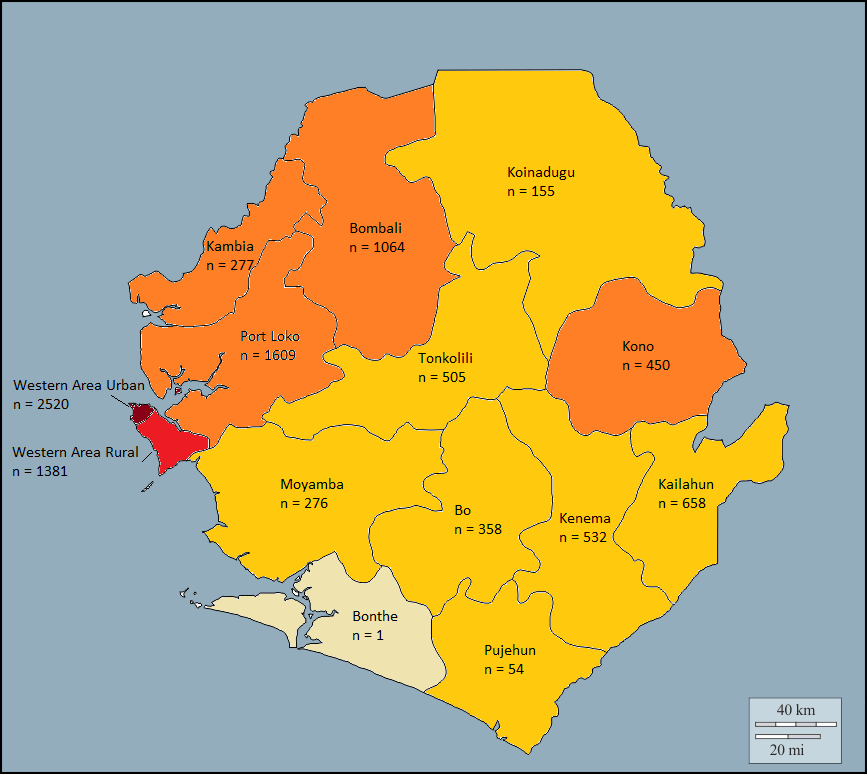

Supplement: Supplementary file 1 [file viruses-11-00071-s001.zip › supplementary/Figure S1.tif]

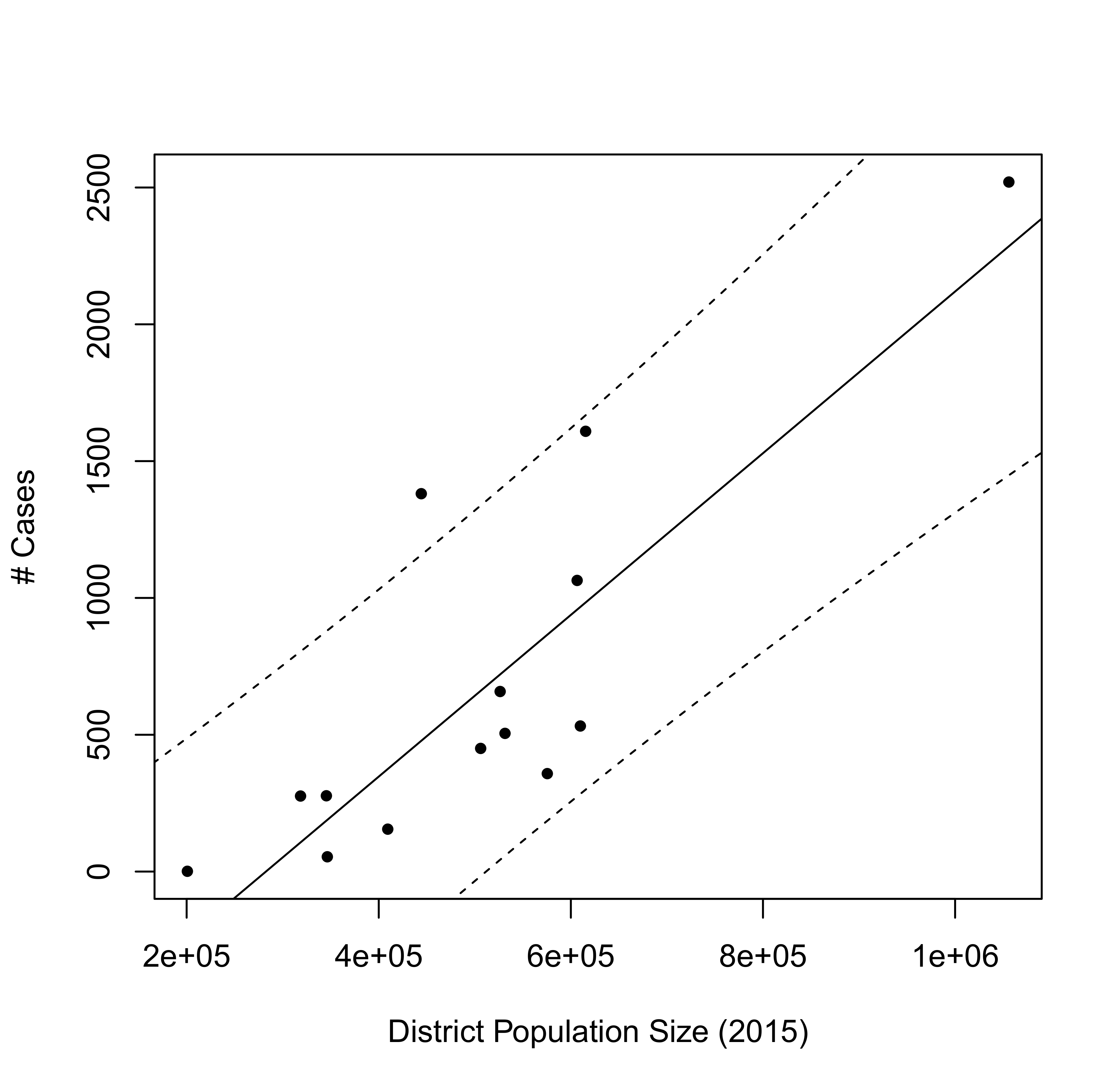

Supplement: Supplementary file 1 [file viruses-11-00071-s001.zip › supplementary/Figure S2.tiff]

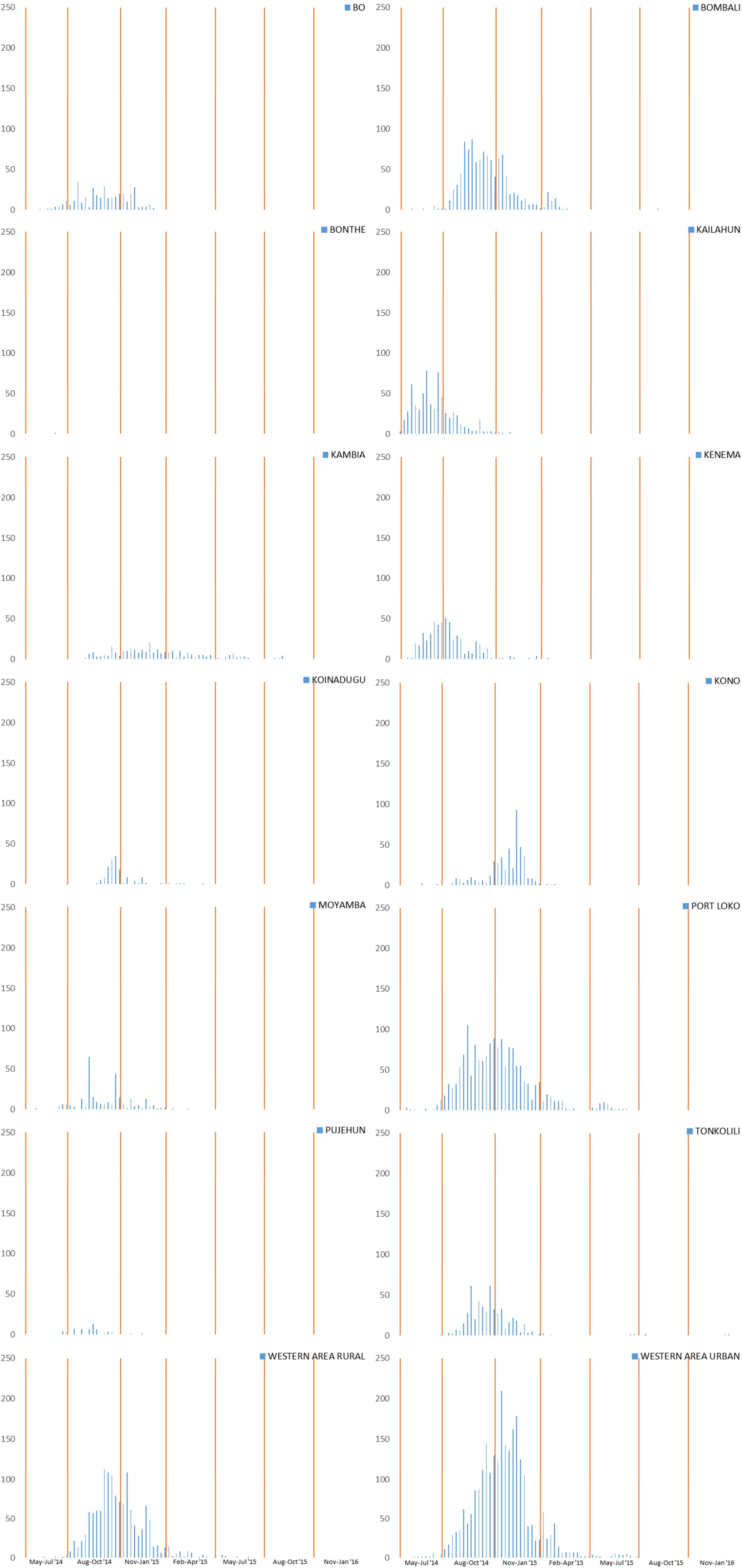

Supplement: Supplementary file 1 [file viruses-11-00071-s001.zip › supplementary/Figure S3.tiff]

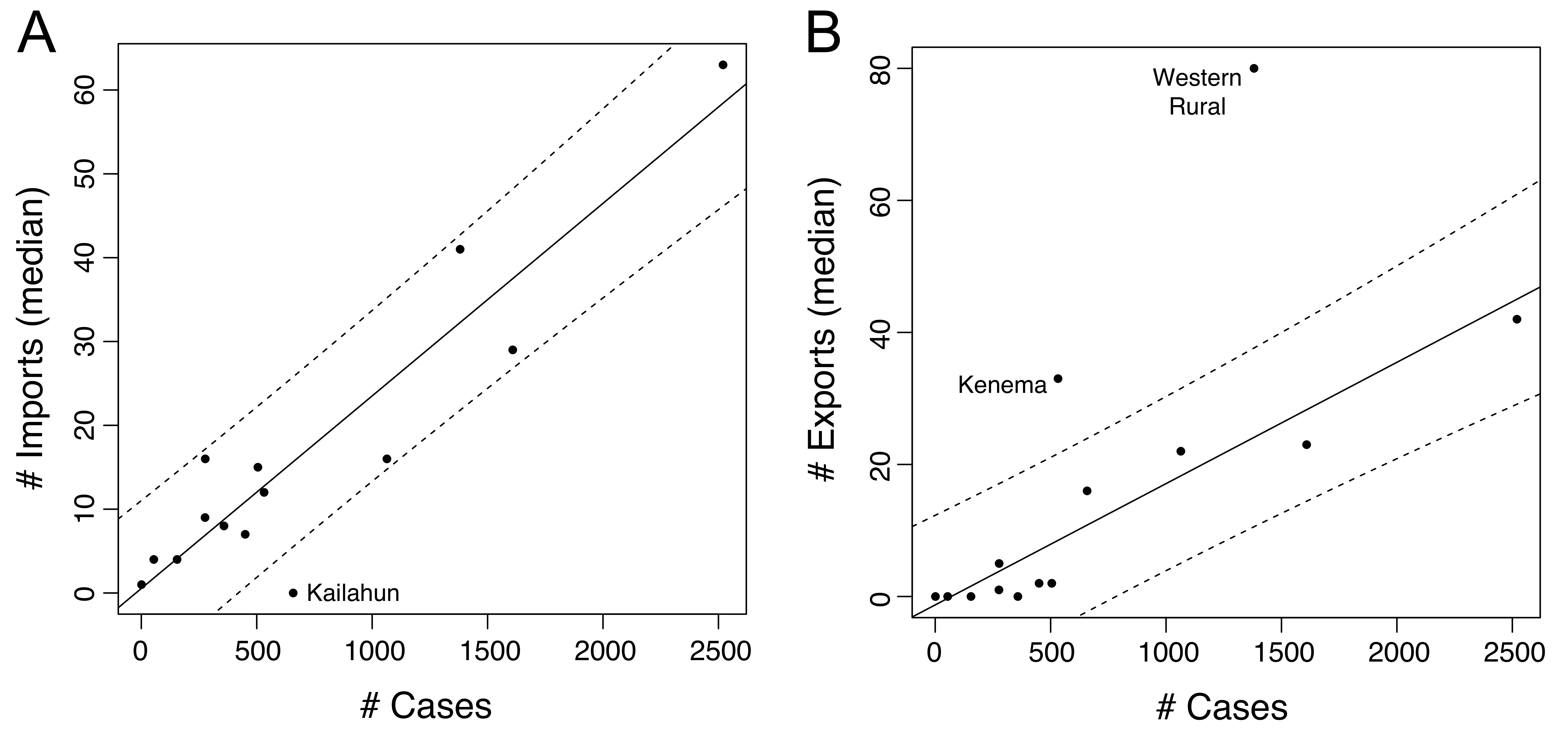

Supplement: Supplementary file 1 [file viruses-11-00071-s001.zip › supplementary/Figure S4a-b.tiff]

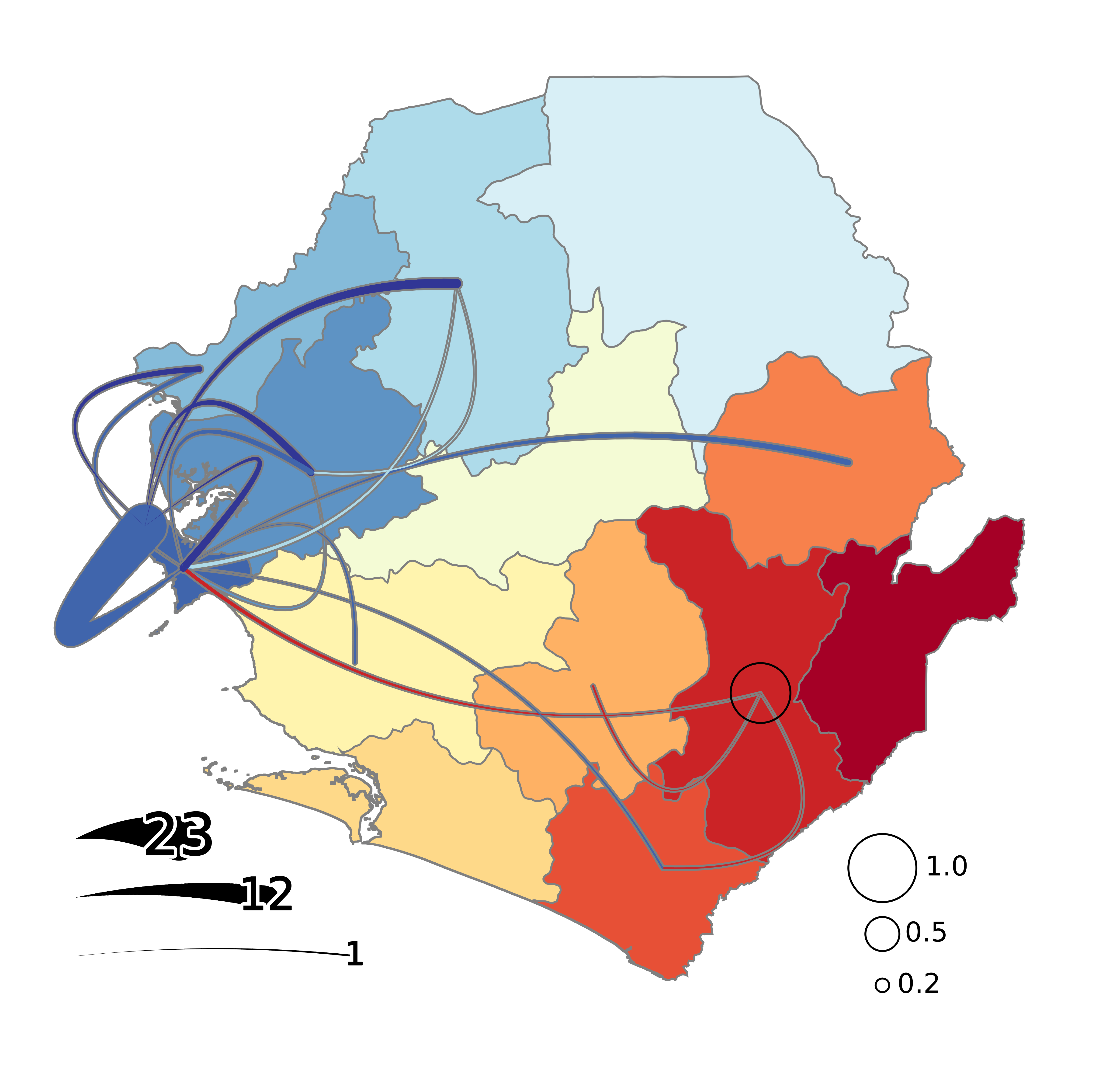

Supplement: Supplementary file 1 [file viruses-11-00071-s001.zip › supplementary/Figure S5.tiff]

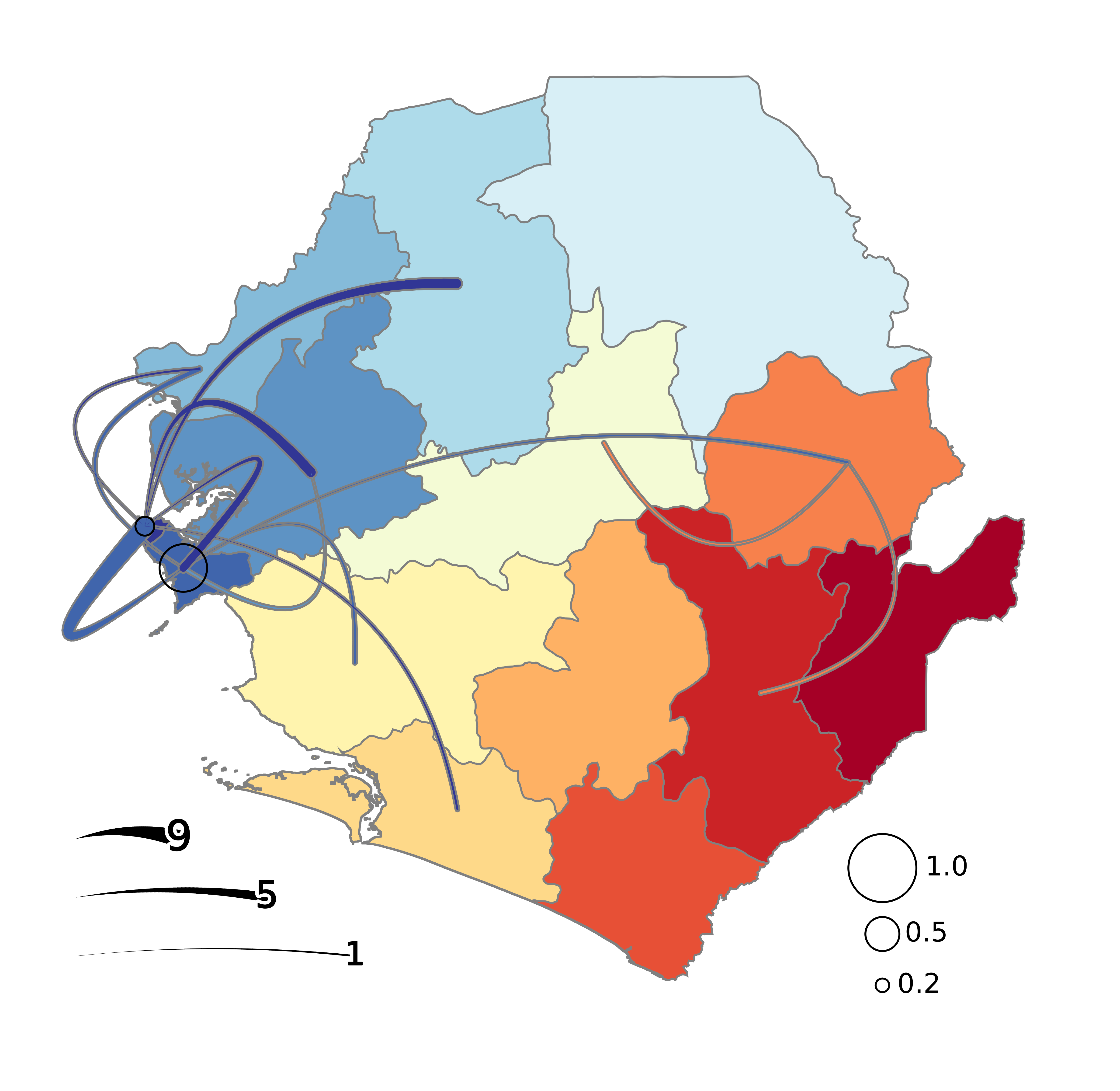

Supplement: Supplementary file 1 [file viruses-11-00071-s001.zip › supplementary/Figure S6.tiff]

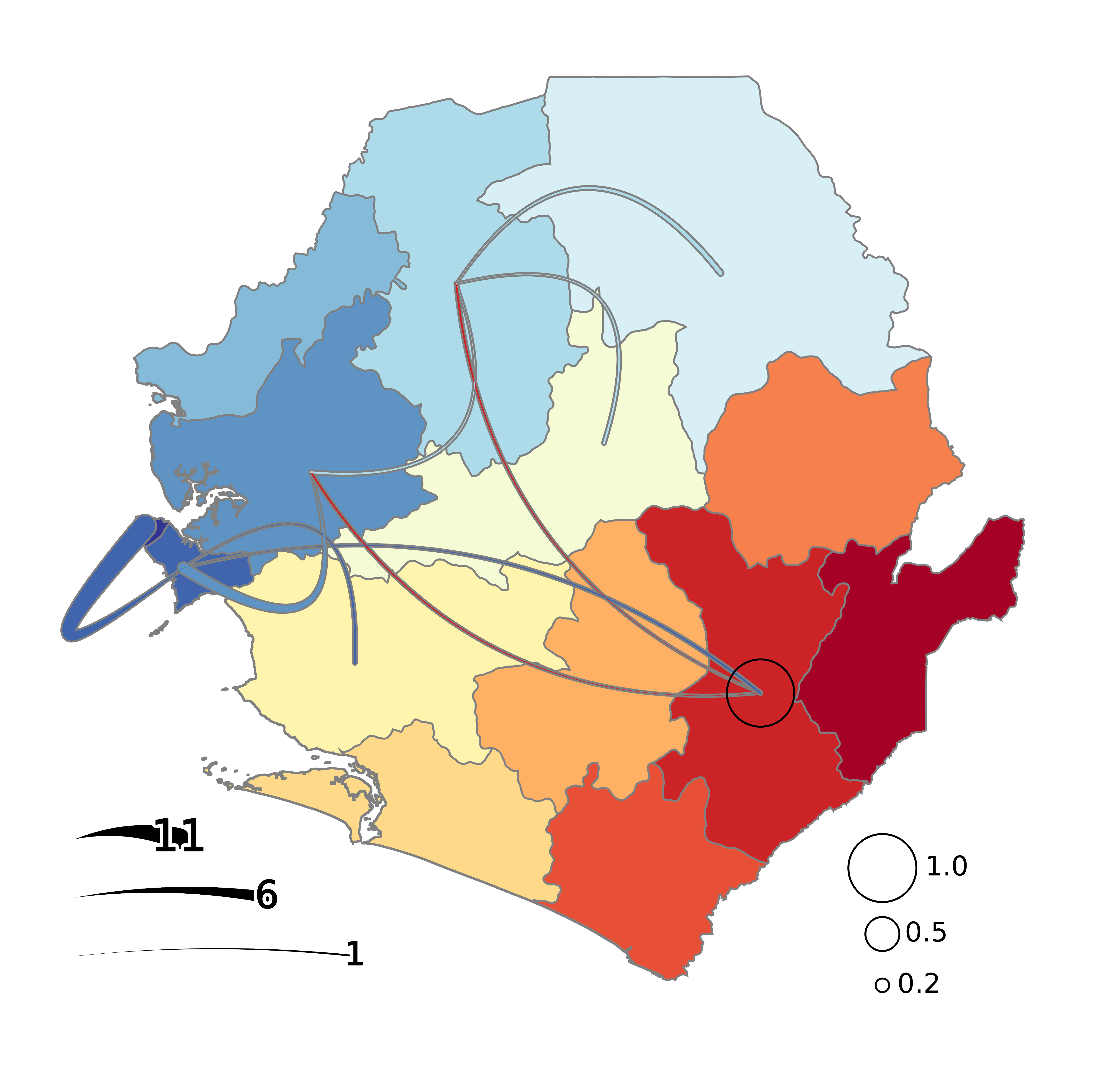

Supplement: Supplementary file 1 [file viruses-11-00071-s001.zip › supplementary/Figure S7.tiff]

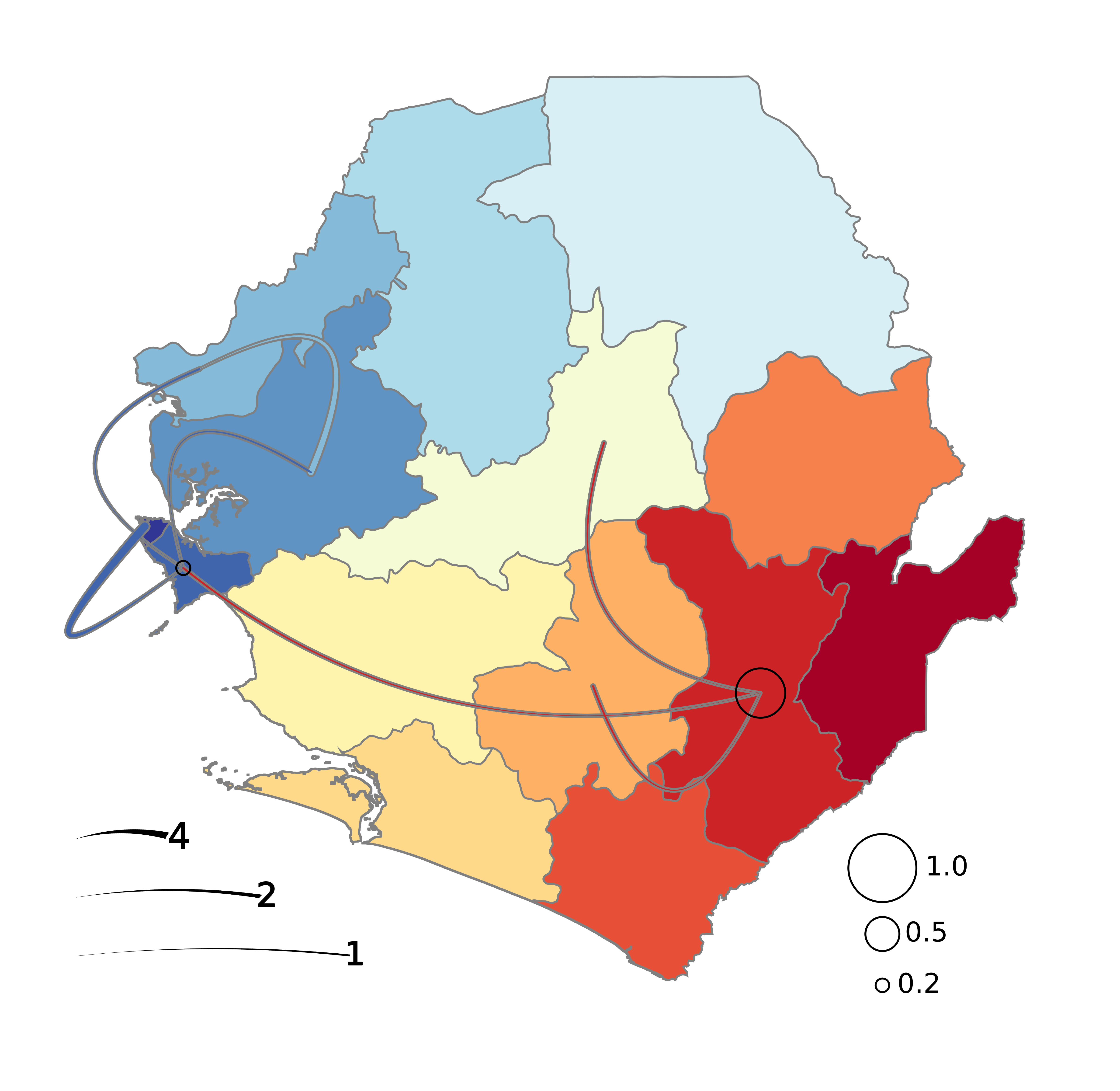

Supplement: Supplementary file 1 [file viruses-11-00071-s001.zip › supplementary/Figure S8a.tiff]

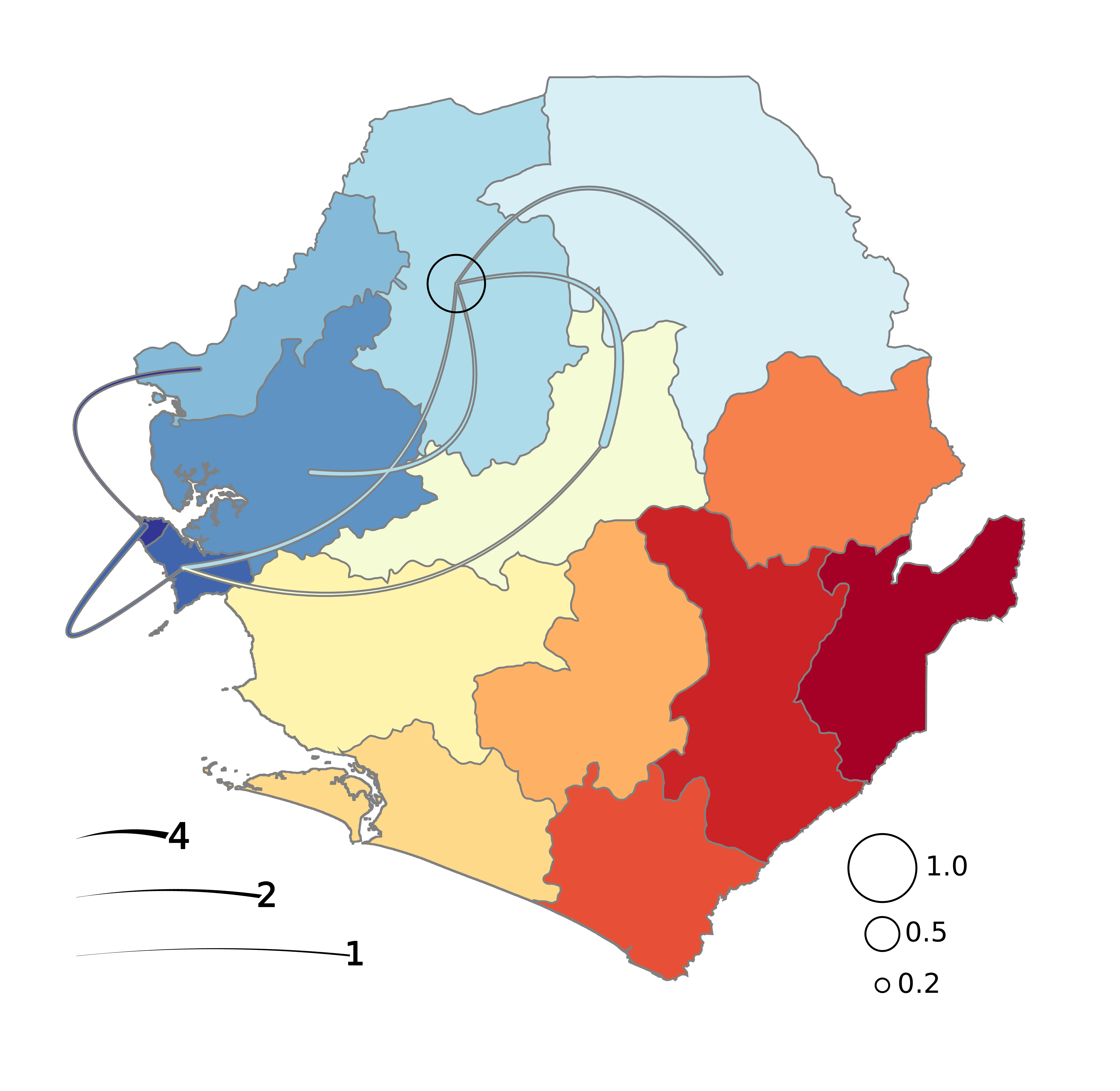

Supplement: Supplementary file 1 [file viruses-11-00071-s001.zip › supplementary/Figure S8b.tiff]

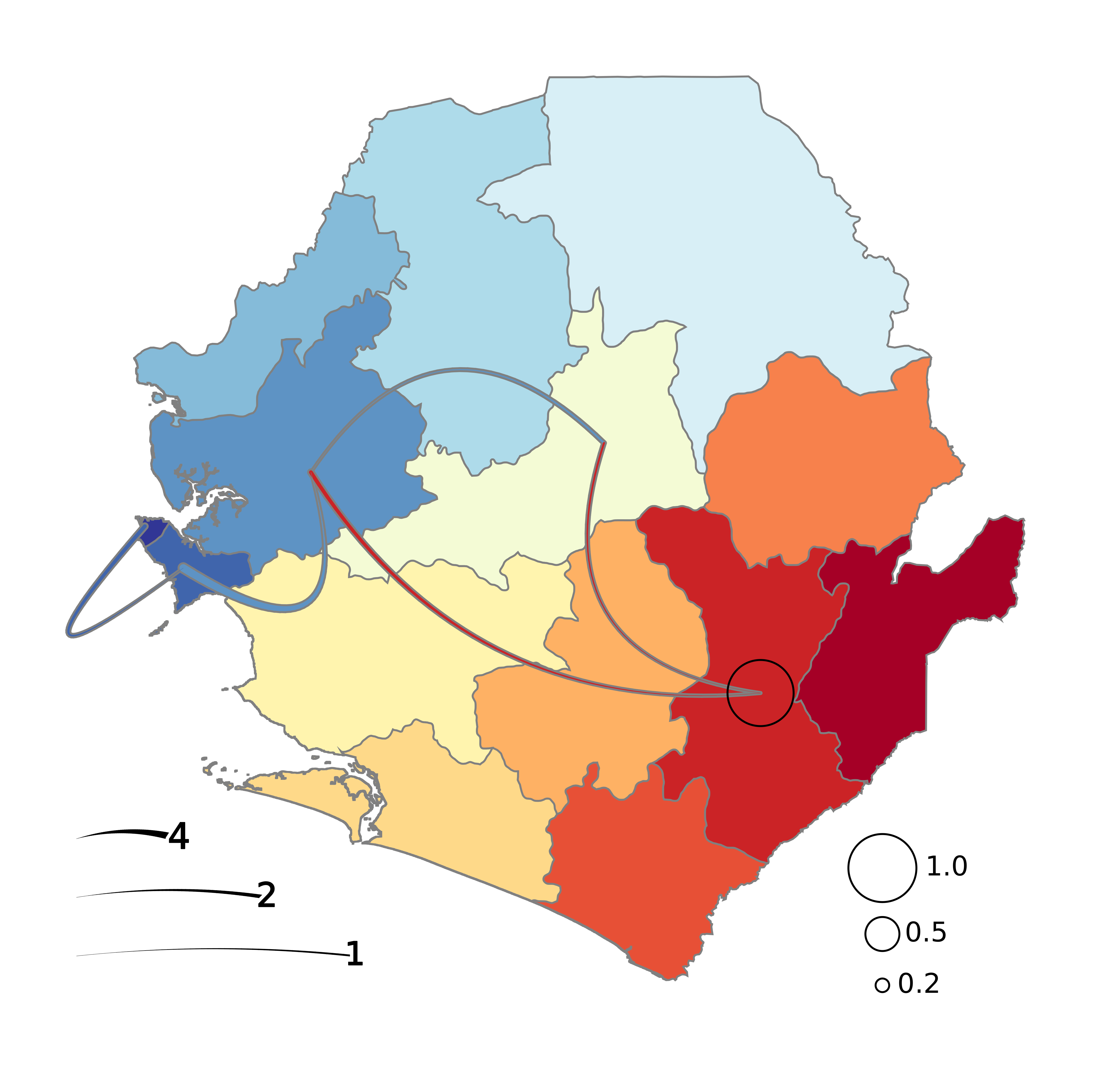

Supplement: Supplementary file 1 [file viruses-11-00071-s001.zip › supplementary/Figure S8c.tiff]

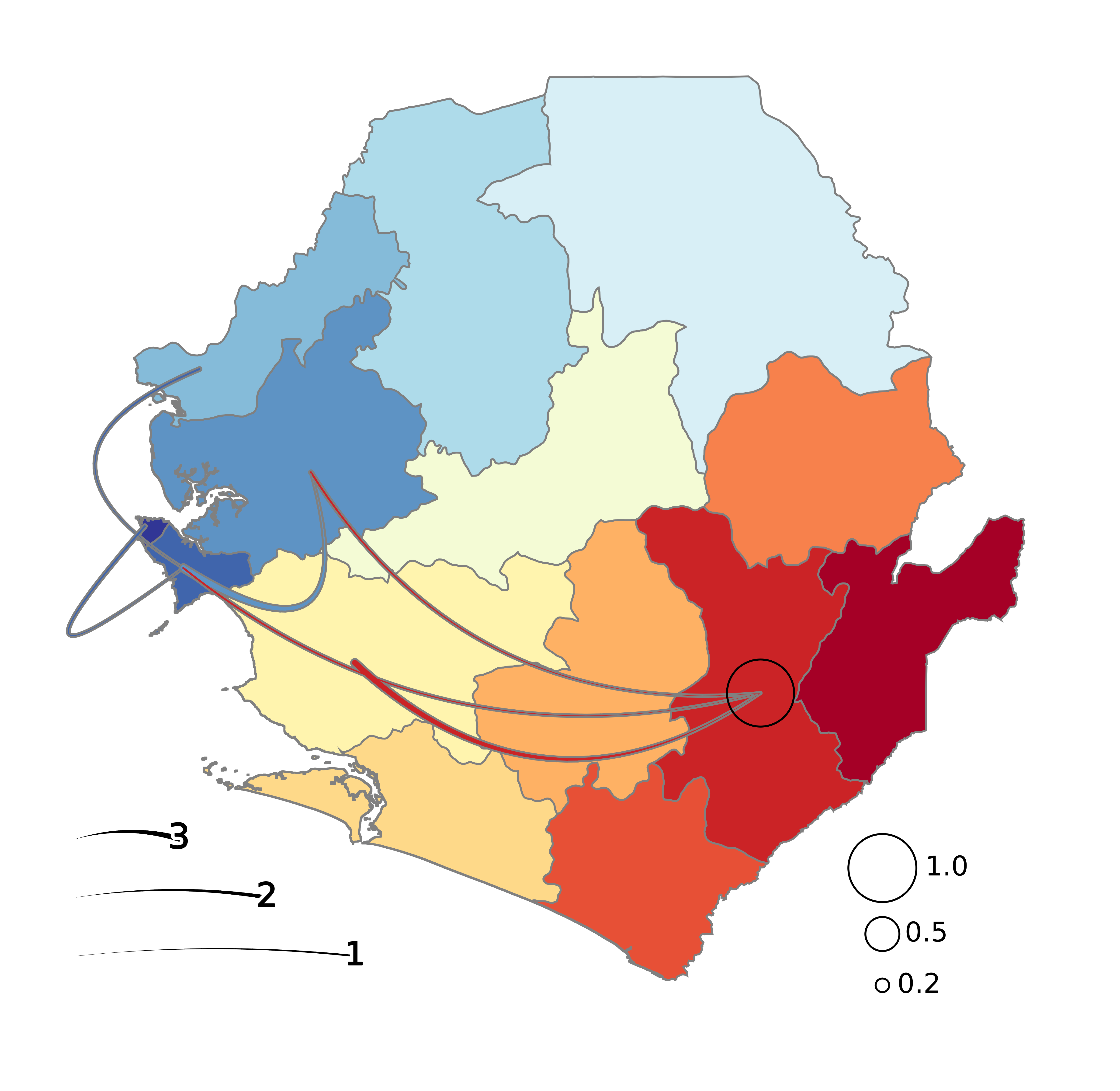

Supplement: Supplementary file 1 [file viruses-11-00071-s001.zip › supplementary/Figure S8d.tiff]
